# Molecular Basis of C-30 Product Regioselectivity of Legume Oxidases Involved in High-Value Triterpenoid Biosynthesis

**DOI:** 10.3389/fpls.2019.01520

**Published:** 2019-11-26

**Authors:** Much Zaenal Fanani, Ery Odette Fukushima, Satoru Sawai, Jianwei Tang, Masato Ishimori, Hiroshi Sudo, Kiyoshi Ohyama, Hikaru Seki, Kazuki Saito, Toshiya Muranaka

**Affiliations:** ^1^Department of Biotechnology, Graduate School of Engineering, Osaka University, Suita, Japan; ^2^Department of Biotechnology, Faculty of Life Sciences, Universidad Regional Amazónica IKIAM, Tena, Ecuador; ^3^RIKEN Center for Sustainable Resource Science, Yokohama, Japan; ^4^Graduate School of Pharmaceutical Sciences, Chiba University, Chiba, Japan; ^5^Tokiwa Phytochemical Co., Ltd., Sakura, Japan; ^6^Department of Chemistry and Materials Science, Tokyo Institute of Technology, Meguro, Japan

**Keywords:** chemodiversity, cytochrome P450 monooxygenase, legume, product regioselectivity, triterpene

## Abstract

The triterpenes are structurally diverse group of specialized metabolites with important roles in plant defense and human health. Glycyrrhizin, with a carboxyl group at C-30 of its aglycone moiety, is a valuable triterpene glycoside, the production of which is restricted to legume medicinal plants belonging to the *Glycyrrhiza* species. Cytochrome P450 monooxygenases (P450s) are important for generating triterpene chemodiversity by catalyzing site-specific oxidation of the triterpene scaffold. CYP72A154 was previously identified from the glycyrrhizin-producing plant *Glycyrrhiza uralensis* as a C-30 oxidase in glycyrrhizin biosynthesis, but its regioselectivity is rather low. In contrast, CYP72A63 from *Medicago truncatula* showed superior regioselectivity in C-30 oxidation, improving the production of glycyrrhizin aglycone in engineered yeast. The underlying molecular basis of C-30 product regioselectivity is not well understood. Here, we identified two amino acid residues that control C-30 product regioselectivity and contribute to the chemodiversity of triterpenes accumulated in legumes. Amino acid sequence comparison combined with structural analysis of the protein model identified Leu149 and Leu398 as important amino acid residues for C-30 product regioselectivity. These results were further confirmed by mutagenesis of CYP72A154 homologs from glycyrrhizin-producing species, functional phylogenomics analyses, and comparison of corresponding residues of C-30 oxidase homologs in other legumes. These findings could be combined with metabolic engineering to further enhance the production of high-value triterpene compounds.

## Introduction

The triterpenoids are a large group of plant specialized metabolites consisting of six isoprene units. Plants produce structurally diverse triterpenoids that often have important roles in plant defense (Osbourn, 1996; Kuzina et al., 2009; Liu et al., 2019). Moreover, some triterpenoids exhibit properties beneficial for human health (Ito et al., 1988; Kenarova et al., 1990; Zhao et al., 2006; Kojoma et al., 2010). Due to this structural diversity, triterpenoids are considered important sources for new drug leads (Geisler et al., 2013; Vo et al., 2017). However, harnessing the potential of the structural diversity of triterpenoids has been hampered by limited information regarding the molecular mechanisms underlying their structural diversity.

Decoration of the triterpene scaffold catalyzed by cytochrome P450 monooxygenases (P450s) is the second step of triterpene biosynthesis (Seki et al., 2015). Generally, P450s have the ability to stereo- and regioselectively oxidize non-activated carbon by introducing various functional groups, such as hydroxyl, carbonyl, carboxyl, and even epoxy moieties (Qi et al., 2006; Ghosh, 2017). Moreover, the introduction of a hydroxyl group into the triterpene scaffold allows to the generation of glycosylated and acylated triterpenes (Osbourn et al., 2011; Seki et al., 2015). Therefore, P450s are believed to play important roles in the diversity of triterpene structures (Ghosh, 2017; Miettinen et al., 2017).

Glycyrrhizin is a triterpene saponin that is the main active compound in legume medicinal *Glycyrrhiza* plants (Shibata, 2000). In addition to its sweet taste (150 times sweeter than sucrose; Kitagawa, 2002), glycyrrhizin also shows various pharmacological activities (Shibata, 2000), including anti-inflammatory (Kroes et al., 1997), hepatoprotective (Jeong et al., 2002), and antiviral effects (Ito et al., 1988). Among the *Glycyrrhiza* species, *Glycyrrhiza uralensis*, *Glycyrrhiza glabra*, and *Glycyrrhiza inflata* are known to produce glycyrrhizin (Hayashi et al., 2000). Large amounts of glycyrrhizin accumulate in their roots and stolons, accounting for an estimated 2%–8% of the dry weight (Shibata, 2000). Glycyrrhizin itself has been used as an ingredient in a number of commercial products, including foods, personal health care products, and medicines. However, the production of glycyrrhizin is dependent on natural resources that require an approximately 2–3-year growth period before harvesting (Chen et al., 2014). Due to the economic value and market demand for licorice, overexploitation of wild licorice has led to significant environmental issues (Marui et al., 2011). Therefore, a rapid and environmental friendly system for glycyrrhizin production is required.

Metabolic engineering has been studied extensively for production of plant specialized metabolites in engineered organisms. The biosynthesis of glycyrrhizin involves the initial cyclization of 2,3-oxidosqualene to the pentacyclic triterpene β-amyrin, followed by a series of oxidative reactions at positions C-11 and C-30 (Seki et al., 2008; Seki et al., 2011). Previously, we identified two P450s (CYP88D6 and CYP72A154) involved in glycyrrhizin biosynthesis (Seki et al., 2008; Seki et al., 2011). Functional characterization of CYP72A154 showed that this enzyme catalyzed oxidation at C-30, accompanied by the production of isomers as minor products (Seki et al., 2011). *Medicago truncatula* does not produce glycyrrhizin, and the homologous enzyme, CYP72A63, showed superior regioselective oxidization of the C-30 position only (Seki et al., 2011). Use of CYP72A63 for production of glycyrrhizin aglycone in engineered yeast further enhanced its yield (Zhu et al., 2018). Although improvement of glycyrrhizin aglycone production in yeast has been achieved, it still produces a number of byproducts, such as 11α,30-dihydroxy-β-amyrin and 30-hydroxy-β-amyrin (Zhu et al., 2018). Further improvement of glycyrrhizin production by combining metabolic engineering and protein engineering is hampered by limited knowledge regarding the structure–function relationship of P450s involved in glycyrrhizin biosynthesis.

A great deal of research effort has focused on discovering the P450s involved in decoration of the triterpene skeleton. Based on amino acid sequence identity, CYP72A154 and CYP72A63 are classified as members of the CYP72A subfamily. The CYP72A subfamily is known as P450 subfamily enzyme involved in generating triterpene chemodiversity, where they catalyze site-specific oxidation of the oleanane-type triterpenoid scaffolds, C-2β (Biazzi et al., 2015), C-21β (Yano et al., 2017; Leveau et al., 2019), C-22β (Ebizuka et al., 2011; Fukushima et al., 2013), C-23 (Fukushima et al., 2013; Biazzi et al., 2015; Liu et al., 2019), and C-30 (Seki et al., 2011). Prall et al. (2016)reported that the CYP72A subfamily in flowering plants showed high variability of amino acid residues among substrate recognition sites (SRSs). However, there have been no experimental studies investigating the roles of amino acid residues in the SRSs in this subfamily. Moreover, no CYP72A subfamily protein crystal structures have yet been reported, even for closely related P450 with more than 40% identity. Therefore, it is still difficult to apply rational protein engineering to the CYP72A subfamily to improve product specificity and regioselectivity. Some reports suggested that gene mining of publicly available genomic or transcriptome databases is a more practical method for obtaining candidate genes encoding CYP72A enzymes showing better catalytic activity (Moses et al., 2014; Suzuki et al., 2018; Zhu et al., 2018). However, the majority of natural enzymes still exhibit some properties unfavorable for application in metabolic engineering, such as low product specificity and regioselectivity (Jung et al., 2011; Goldsmith and Tawfik, 2017). Therefore, it would be useful and interesting to determine the molecular mechanism underlying product regioselectivity of C-30 oxidases involved in high-value triterpenoid biosynthesis.

In this study, we mined the CYP72A subfamily from *M. truncatula* and characterized its enzymatic activity against β-amyrin. Functional characterization of the CYP72A subfamily from *M. truncatula* showed that CYP72A63 is an enzyme with high C-30 product regioselectivity. Interestingly, CYP72A62v2 and CYP72A64v2, which share more than 90% amino acid sequence identity, showed completely different product regioselectivity. By comparing the SRS sequences of CYP72A63 and its homologs, and by protein homology modeling, we identified Leu149 and Leu398 as key amino acid residues responsible for C-30 regioselective oxidation activity in CYP72A63. Analysis of CYP72A154 variants from both glycyrrhizin-producing and non-glycyrrhizin-producing *Glycyrrhiza* species also indicated that amino acid residue #398 differentiated the product regioselectivity of CYP72A154 variants. The results of this study will provide opportunities to engineer P450s for manipulation of product regioselectivity by rational protein engineering, to achieve the production of valuable triterpenoids such as glycyrrhizin.

## Materials and Methods

### Plant Materials

The seed plants used in this experiment are listed in **Supplementary Table 1**. Seeds were germinated by mechanical scarification and imbibition in the dark at 23°C for 2 days. Germinated seeds were planted in soil and grown in a plant room with a controlled temperature of 23°C under a 16-h light/8-h dark photoperiod. Plant samples for RNA isolation were collected from 4-week-old seedlings, immediately frozen in liquid nitrogen, and then stored at -80°C until use. Underground parts of *Glycyrrhiza pallidiflora* were harvested from the Medicinal Plant Garden of Chiba University (Chiba, Japan); *Glycyrrhiza lepidota* and *Glycyrrhiza macedonica* were obtained from Osaka University of Pharmaceutical Science (Osaka, Japan), and *Glycyrrhiza glabra* was obtained from Health Sciences University of Hokkaido (Hokkaido, Japan).

### Authentic Standards

β-Amyrin was purchased from Extrasynthese (Lyon, France). Sophoradiol, 30-hydroxy-β-amyrin, and 11-deoxoglycyrrhetinic acid were synthesized as described in our previous report (Seki et al., 2008).

### Gene Mining for the CYP72A Subfamily

CYP72A subfamily candidates were identified by BLAST search using the amino acid sequences of CYP72A61v2, CYP72A63, and CYP72A67 as queries against the *M. truncatula* genome project Mt4.0v1 proteins (Tang et al., 2014). Hits showing >50% identity with unique sequential gene ID numbers were manually checked for surrounding 50 kb in genome JBrowser (Krishnakumar et al., 2015). Natural variants of gene cluster subgroup III were mined from *M. truncatula* Hapmap (Zhou et al., 2017) using the genomic sequences of *CYP72A62* (Medtr8g042060), *CYP72A63* (Medtr8g042040), *CYP72A64* (Medtr8g042020), and *CYP72A65* (Medtr8g042000) (obtained from *M. truncatula* genome database) as queries. Amino acid sequences were predicted according to the known coding sequences of gene references in *M. truncatula*. CYP72A63 homologs from other legumes were mined from publicly available genomic sequences in the 1KP database (Matasci et al., 2014), Clover GARDEN (www.clovergarden.jp/) (Hirakawa et al., 2016), Vigna Genome Server (viggs.dna.affrc.go.jp/) (Sakai et al., 2016), Cool Season Food Legume Genome Database (www.coolseasonfoodlegume.org/) (Main et al., 2013), and Legume Information System (legumeinfo.org/) (Dash et al., 2016).

### Cloning and Vector Construction

RNA preparation and cloning methods are explained briefly in the **Supplementary Methods**. All candidates were verified by sequencing and submitted to the P450 Committee for naming. Yeast expression clones, using pELC-CPR-GW (Seki et al., 2008), pYES2-DEST52 (Thermo Fisher Scientific, Waltham, MA), and a Gateway-compatible version of pESC-HIS (Seki et al., unpublished) as destination vectors, were constructed by LR reaction using LR clonase II Enzyme mix (Thermo Fisher Scientific).

### Accession Numbers

Sequence data from this experiment have been submitted to the DNA DataBank of Japan (DDBJ), the European Nucleotide Archive (ENA), and GenBank databases under the following accession numbers: MK534530 (CYP72A695), MK534531 (CYP72A696), MK534532 (*Gmax*CYP72A141), MK534533 (*Gg*CYP72A154), MK534534 (*Gp*CYP72A154), MK534535 (*Lc*CYP72A698), MK534536 (*Ps*CYP72A698), MK534537 (CYP72A302), MK534538 (CYP72A694), MK534539 (CYP72A697), MK534540 (CYP72A336v2), MK534541 (CYP72A70), MK534542 (*Gsoja*CYP72A141), MK534543 (CYP72A337v2), MK534544 (CYP72A557), MK534545 (CYP72A558), MK534546 (CYP72A559), MK534547 (CYP72A560), MK534548 (CYP72A64v2), MK534549 (CYP72A699), MK792941 (CYP72A66v2).

### *In Vivo* Enzymatic Assay in Yeast

*In vivo* enzymatic assay in yeast was performed by co-expression of *Lotus japonicus* cytochrome P450 reductase (CPR) and β-amyrin synthase (OSC1), with three yeast expression clones for each of the CYP72A subfamily genes. Each corresponding set of pELC-CPR-CYP72A, pYES2-DEST52-CYP72A, and pESC-HIS-CYP72A was transformed into *Saccharomyces cerevisiae* INVSc1 (*MATa his3D1 leu2 trp1-289 ura3-52*; Thermo Fisher Scientific) carrying pYES3-ADH-OSC1 (Seki et al., 2008) using Frozen-EZ Yeast Transformation II^™^ (Zymo Research, Orange, CA), and a yeast strain harboring the three empty vectors (pELC-CPR-GW, pYES2-DEST52, and pESC-HIS) was used as a control. All genes tested in this experiment are listed in **Supplementary Table 3**. *In vivo* enzymatic assay in yeast was performed as reported previously (Fukushima et al., 2011) with some modifications. Yeast strains were pre-cultured in appropriate synthetic defined medium (Clontech, Palo Alto, CA) containing 2% glucose, and incubated overnight at 30°C, 200 rpm. Aliquots of 50 µl of yeast pre-cultures were added into 5 ml of appropriate synthetic defined medium (Clontech) containing 2% glucose and incubated overnight at 30°C, 200 rpm. Yeast cells were collected by centrifugation, resuspended in 5 ml of appropriate synthetic defined medium (Clontech) containing 2% galactose and incubated at 30°C for 4 days at 200 rpm. Yeast metabolites were extracted using ethyl acetate after sonication, three times for 30 minutes each time, and portions of the extracts were analyzed by gas chromatography–mass spectrometry (GC-MS) after derivatization with *N*-methyl-*N*-(trimethylsilyl)trifluoroacetamide (Sigma-Aldrich, St. Louis. MO). GC-MS analysis was performed using a gas chromatograph (7890B; Agilent Technologies, Santa Clara, CA) connected to a mass spectrometer (5977A; Agilent Technologies) and HP-5MS capillary column (0.25 mm × 30 m, 0.25 µm) (Agilent Technologies). The initial oven temperature was 150°C with a hold time of 1 min, increasing from 150°C to 260°C at 30°C/min and 260°C to 300°C at 1°C/min. Samples were injected in splitless mode with an injection temperature of 250°C, with helium as the carrier gas at a flow rate of 1.0 ml/min. Comparison of retention times and mass fragmentation patterns of detectable compounds with those of authentic standards was performed to assign the peaks.

### Bioinformatics Analyses

The predicted amino acid sequences of identified CYP72A subfamily members were used for multiple sequence alignment using ClustalW in MEGA7 (Kumar et al., 2016). A neighbor-joining tree was generated using MEGA6 with the Jones–Taylor–Thomson substitution model and bootstrap analysis of 1,000 replicates. The SRSs of the *M. truncatula* CYP72A subfamily were predicted as described previously (Gotoh, 1992; Prall et al., 2016). A CYP72A63 structure model was constructed using 6C93.A as the template with about 27% identity in SWISS-MODEL (Arnold et al., 2006). Heme was docked with 40 × 40 × 40 grid points, spacing 0.375, and grid center -14.738; 21.827; 9.827 using Autodock4.0 (Morris et al., 2009). PyMol (Schrödinger, 2017) was used to visualize the amino acids in the active site of the enzyme.

### Site-directed Mutagenesis

Designed protein variants were generated using the site-specific nucleotides listed in **Supplementary Table 2**. Mutagenesis experiments were performed using a PrimeSTAR Mutagenesis Basal Kit (TaKaRa Bio, Kyoto, Japan) and the entry clone was used as a template.

### Elucidation of the Structure of Compound 2 (Peak 2)

A yeast strain carrying β-amyrin synthase co-expressed together with CPR and CYP72A63^L149V/L398V^ was cultured in appropriate synthetic defined medium with a total volume of 25.2 L (250 ml × 72, 150 ml × 48). Yeast metabolites were obtained by saponification prior to extraction with *n*-hexane three times. Yeast extracts were evaporated and the residues were applied to silica gel chromatography (60N, spherical, neutral) (Kanto Chemical, Tokyo, Japan). Hexane:ethyl acetate (1:9) was used as the mobile phase, and afforded about 8 mg of compound 2. Nuclear magnetic resonance (NMR) data were recorded on a Bruker Avance III 600 MHz spectrometer (Bruker Daltonic, Bremen, Germany) using CDCl_3_ as the solvent.

## Results

### The Chromosomal Localization of *M.truncatula* CYP72As Corresponds to the Phylogenetic Tree Topology

Using representatives of each subgroup in the CYP72A subfamily described previously (Seki et al., 2011), we identified 20 candidate genes encoding CYP72A subfamily enzymes in the *M. truncatula* genome. *CYP72A59-like6*, *CYP72A59-like7*, and *CYP72A68-like* showed shorter sequences and no transcripts in transcriptome data (**Supplementary Table 4**). We considered them to be pseudogenes and they were excluded from subsequent experiments. Among the 17 genes identified as encoding CYP72A subfamily enzymes from *M. truncatula* Mt4.0v1, only eight (*CYP72A59v2*, *CYP72A61*, *CYP72A62v2*, *CYP72A63*, *CYP72A65v2*, *CYP72A67*, *CYP72A68-430*, *CYP72A68-470*) were functionally characterized (Seki et al., 2011; Fukushima et al., 2013; Biazzi et al., 2015; Reed et al., 2017). In summary, nine genes (*CYP72A64v2*, *CYP72A66v2*, *CYP72A336v2*, *CYP72A337v2*, *CYP72A70*, *CYP72A557*, *CYP72A558*, *CYP72A559*, *CYP72A560*) were reported in this study.

**Figure 1:**
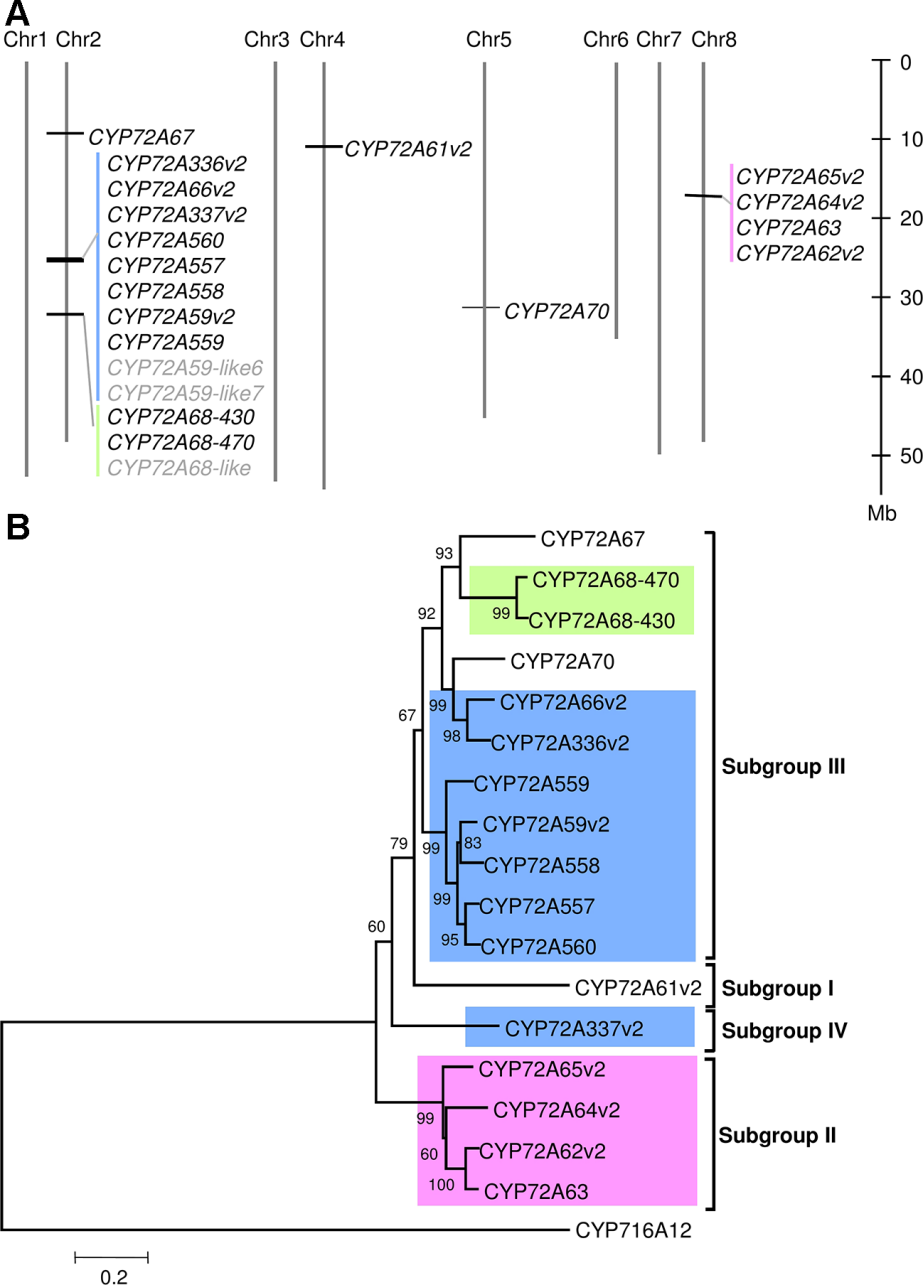
CYP72A subfamily genes in *M. truncatula*. **(A)** Chromosomal localization of CYP72A subfamily in *M. truncatula*. Chromosomal localization was determined according to the Mtr4.0 database JBrowser feature. Each cluster is indicated in a different color. P450s shown in gray indicate pseudogenes. **(B)** Phylogenetic tree of CYP72A subfamily. A neighbor-joining tree was constructed using amino acid sequences with bootstrap analysis of 1,000 replicates. Members of the CYP72A subfamily located in the same cluster are indicated by the same background color. The subgroup classification based on Seki et al. (2011)is indicated.

In the BlastP results, hits had unique sequential gene ID numbers (**Supplementary Table 5**). Based on this observation, we next analyzed the chromosomal localization of the CYP72A subfamily in the *Medicago truncatula* Mt4.01 genome database using JBrowser (Krishnakumar et al., 2015). The CYP72A subfamily genes were shown to be clustered in tandem arrays on chromosomes 2 and 8, with the exception of *CYP72A67*, *CYP72A61*, and *CYP72A70* (**Figure 1A**). Notably, the gene cluster on chromosome 2 contains half of the total number of CYP72A subfamily genes present in the *M. truncatula* genome. The CYP72A subfamily enzymes within the cluster showed 81%–94% identity except for CYP72A337 (**Supplementary Table 6**). The constructed phylogenetic tree showed that the CYP72A subfamily genes clustered on chromosomes 2 and 8 are also grouped into the same subgroups (IV and II, respectively; **Figure 1B**). Consistent with its amino acid sequence identity, CYP72A337v2 is located out of the clade within the gene cluster, and was further classified into a new subgroup IV. These results suggested that gene duplication likely occurred multiple times within the CYP72A subfamily in *M. truncatula*.

### CYP72A63 Regioselectively Oxidized β-Amyrin C-30

**Figure 2:**
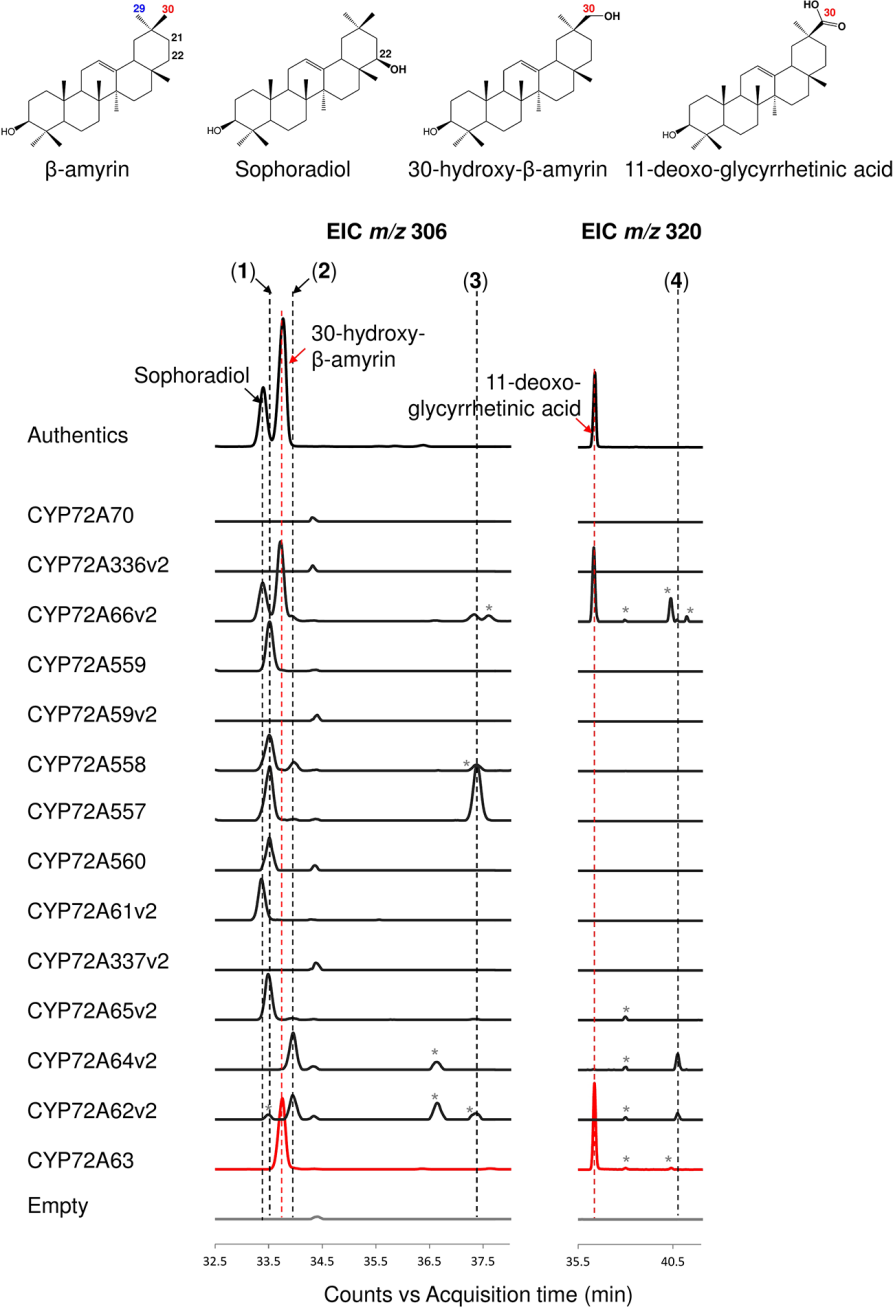
*In vivo* enzymatic activity of CYP72A subfamily enzymes from *M. truncatula* against β-amyrin as the substrate. Triterpene extract was analyzed by gas chromatography coupled with mass spectrometry (extracted ion chromatogram; EIC). Molecular ions with *m/z* 306 and 320 were selected as specific for hydroxylated and carboxylated β-amyrin, respectively. Peaks corresponding to authentic standards are labeled. Peaks likely corresponding to β-amyrin oxidized products are labeled 1–4. Peaks corresponding to unconfirmed β-amyrin oxidized products are indicated with asterisks. Red color indicates C-30 product regioselectivity-related product or enzymes.

The enzymatic activities of CYP72A subfamily enzymes against β-amyrin were characterized by co-expression of CYP72A subfamily genes together with CPR and β-amyrin synthase in *S. cerevisiae* INVSc1. The CYP72A subfamily enzymes within the cluster showed a range of regioselective oxidation activity (**Figure 2**, **Supplementary Figure 1A**). CYP72A61v2 of subgroup I showed oxidation activity against β-amyrin at position C-22β, producing sophoradiol. β-Amyrin C-22β oxidation activity was also detected in CYP72A66v2 (subgroup III), which showed oxidation activity at more than one site producing sophoradiol, 30-hydroxy-β-amyrin, compound **2** (peak 2 in **Figure 2**, which may correspond to a monohydroxylated β-amyrin product with a hydroxyl group on the D or E ring, based on the characteristics of retro Diels-Alder fragmentation at the C ring in the mass fragmentation pattern shown in **Supplementary Figure 1A**), 11-deoxoglycyrrhetinic acid, and some minor unknown compounds. CYP72A557 and CYP72A558 also showed oxidation activity against β-amyrin producing unknown compound 1 (peak 1 in **Figure 2**), compound **2** (peak 2 in **Figure 2**), and unknown compound 3 (peak 3 in **Figure 2**), which were predicted to be monohydroxylated β-amyrin products with a hydroxyl group on the D or E, based on the characteristics of retro Diels-Alder fragmentation at the C ring in the mass fragmentation pattern shown in **Supplementary Figure 1A**. Unlike other enzymes in the same cluster, CYP72A559 and CYP72A560 showed oxidation activity against β-amyrin at the D or E ring, to produce unknown compound **1** (peak 1 in **Figure 2**) alone.

CYP72A subfamily enzymes in tandem array subgroup II showed oxidation activity with different regioselectivities (**Figure 2**, **Supplementary Figure 1A**). CYP72A65v2 showed oxidation activity against β-amyrin, producing unknown compound **1** (peak 1 in **Figure 2**) as the major product and compound **2** (peak 2 in **Figure 2**) as a minor product. CYP72A62v2 and CYP72A64v2 showed oxidation activity against β-amyrin, producing compound **2** (peak 2 in **Figure 2**) and unknown compound 4 (peak 4 in **Figure 2**) as minor carboxylated form products. In addition, unknown compound **1** (peak 1 in **Figure 2**) was detected in trace amounts in the reaction product of CYP72A62v2. CYP72A63 showed oxidation activity against β-amyrin at the C-30 position producing 30-hyroxy-β-amyrin and 11-deoxoglycyrrhetinic acid. The *in vivo* enzymatic assay clearly showed that CYP72A63 was the only one CYP72A subfamily enzyme with high regioselectivity at β-amyrin C-30 from *M. truncatula*.

No oxidized β-amyrin product was detected on *in vivo* enzyme assay of CYP72A70, CYP72A336v2, CYP72A59v2, and CYP72A337v2 (**Figure 2**, **Supplementary Figure 1A**). The lack of detectable enzymatic activity of these four CYP72A enzymes may have different causes; current detection method may not be able to detect trace amounts of its oxidation products, enzymes may not be expressed in correct way, enzymes may have different substrate specificity or mutation in the signature region may cause loss-of-function. Possible mutations in the signature region of these four enzymes were investigated by multiple alignment of the putative oxygen activation region of the CYP72A subfamily (**Supplementary Figure 3**). An amino acid substitution in a conserved acidic amino acid (Glu) to basic amino acid (Lys) was found in CYP72A336v2. To examine whether this substitution of conserved amino acid residue Glu327 caused loss of function in CYP72A336v2, the mutant CYP72A336v2^K327E^ was generated. *In vivo* enzymatic assay of CYP72A336v2^K327E^ showed that substitution of Lys327 to Glu327 could recover enzyme activity of CYP72A336v2, producing sophoradiol, 30-hydroxy-β-amyrin, and compound **2** (peak 2 in **Supplementary Figure 3C**).

### Leu149 and Leu398 are Essential for Regioselective Oxidation at C-30

**Figure 3:**
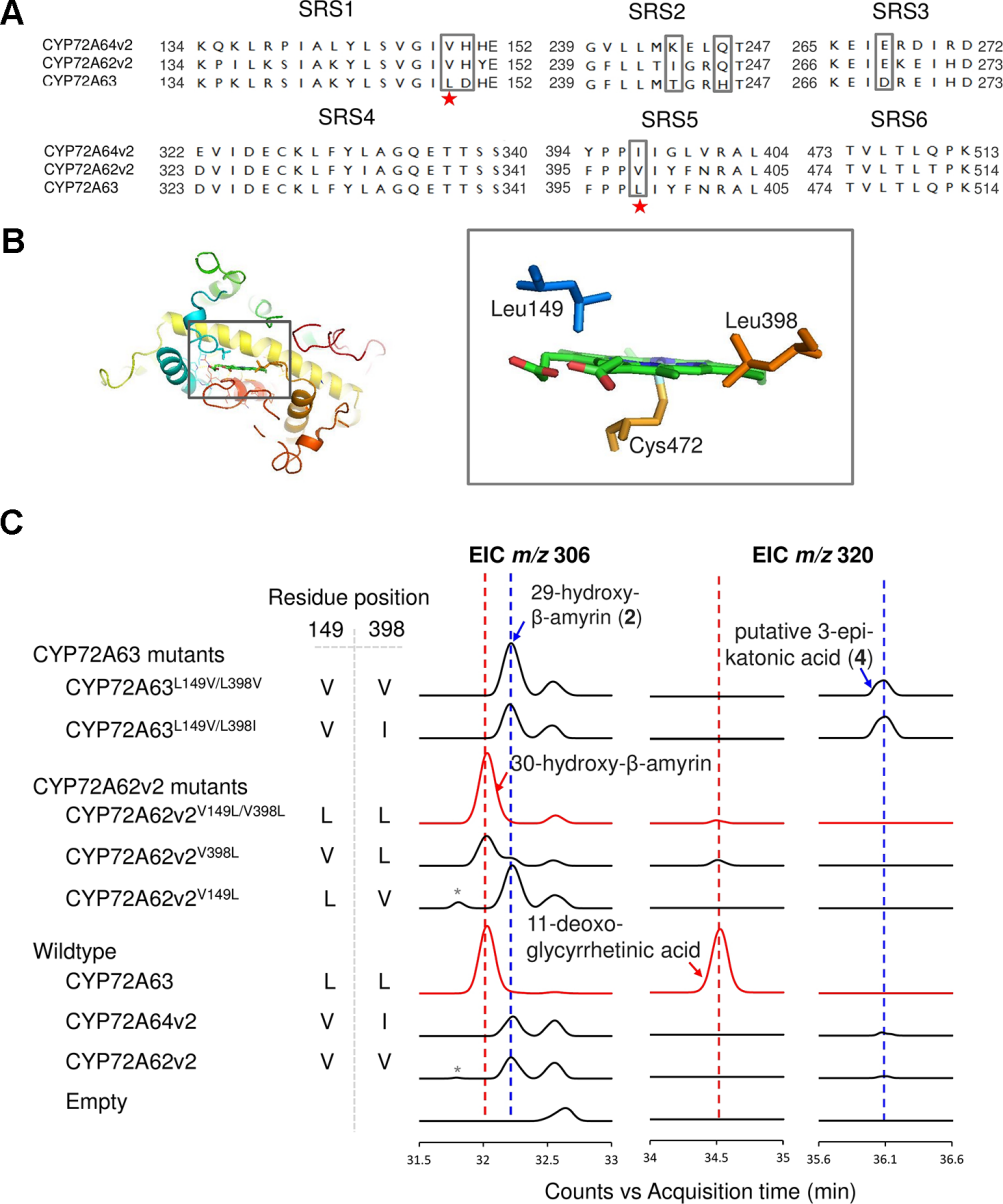
Identification of key amino acid residues controlling C-30 product regioselectivity. **(A)** Comparison of amino acid sequences in the substrate recognition sites (SRSs). Amino acid residue candidates for mutagenesis are indicated by gray boxes. Key residues are indicated by red stars. **(B)** Structural analysis of key amino acid residues in the CYP72A63 model. The gray boxes show enlargements of areas where key residues are located in the active site above heme. **(C)** Reciprocal mutagenesis studies to identify key amino acid residues. Molecular ions with *m/z* 306 and 320 were selected for EIC analysis of β-amyrin-oxidized products. C-30 product regioselectivity-related products or enzymes are indicated in red. Peaks corresponding to unconfirmed β-amyrin are indicated with asterisks.

To identify residues important for C-30 regioselectivity of CYP72A63, we examined amino acid residues in the predicted SRS (**Figure 3A**). A number of criteria were applied; the amino acid residue must be conserved in CYP72A62v2 and CYP72A64v2, but not in CYP72A63, or must not be conserved at all. Six positions (Val149, His150, Ile244, Gln246, Glu269, and Val398) were selected (marked in the box, **Figure 3A**). *In vivo* enzymatic assay of CYP72A62v2 mutants showed that CYP72A62v2^V398L^ was sufficient to alter the product regioselectivity by detecting compound **2** (peak 2) and 30-hydroxy-β-amyrin on GC-MS analysis (**Figure 3C**, **Supplementary Figure 1B**). In parallel, we also mapped the positions of key residues important for C-30 product regioselectivity by generating protein chimeras of CYP72A63 and CYP72A62v2 using the segment exchange approach (**Supplementary Figure 4**). *In vivo* enzymatic assay of chimeric proteins suggested that key amino acid residues for C-30 product regioselectivity are located between residues #133 and #409, and more than one amino acid residue is required (**Supplementary Figure 4**). These results suggested that Leu398 is not the sole residue responsible for C-30 product regioselectivity.

To identify the second important amino acid residue determining product regioselectivity, we next mapped the position of Leu398 in the three-dimensional homology model of CYP72A63 (**Figure 3B**). The homology model of CYP72A63 showed that Leu398 is located in the area surrounding the reaction center of P450, where the enzyme catalytic reaction takes place. Based on these findings, we hypothesized that the second important amino acid residue may be located close to Leu398. Therefore, we examined amino acid residues surrounding Leu398. Among five amino acid residues in this region, Leu149 (SRS1) is located in a relatively face-to-face position to Leu398 (SRS5) (**Figure 3B**). Thus, both amino acid residues may determine the regioselectivity of CYP72A63. To examine the role of Leu149 together with Leu398 in C-30 product regioselectivity, the mutant CYP72A62v2^V149L/V398L^ was generated. *In vivo* enzymatic assay of CYP72A62v2^V149L/V398L^ showed that CYP72A62v2^V149L/V398L^ produced only C-30 oxidized products, 30-hydroxy-β-amyrin and 11-deoxoglycyrrhetinic acid (**Figure 3C**). These results showed that Leu149 and Leu398 are important amino acid residues for regioselective oxidation of β-amyrin at C-30.

Substitution of amino acid residues #149 and #398 markedly altered the product regioselectivity of the CYP72A62v2 enzyme (**Figure 3C**, **Supplementary Figure 1C**). We also generated mutants of CYP72A63, designated as CYP72A63^L149V/L398V^ and CYP72A63^L149V/L398I^, which resembled CYP72A62v2 and CYP72A64v2, respectively. *In vivo* enzymatic assay showed that CYP72A63^L149V/L398V^ and CYP72A63^L149V/L398I^ had altered product regioselectivity resembling their counterparts, CYP72A62v2 and CYP72A64v2, respectively (**Figure 3C**, **Supplementary Figure 1C**). These results clearly showed that Leu149 and Leu398 are important amino acid residues for C-30 product regioselectivity of CYP72A63. To determine the regioselective oxidation activities of CYP72A62v2 and CYP72A64v2, we examined the structure of compound 2 (peak 2) by NMR spectroscopy. Complete^13^ C assignment of purified compound 2 (peak 2) was not achieved due to incomplete removal of impurities. However, the data indicated the presence of 29-hydroxy-β-amyrin (**Supplementary Data**).

**Figure 4:**
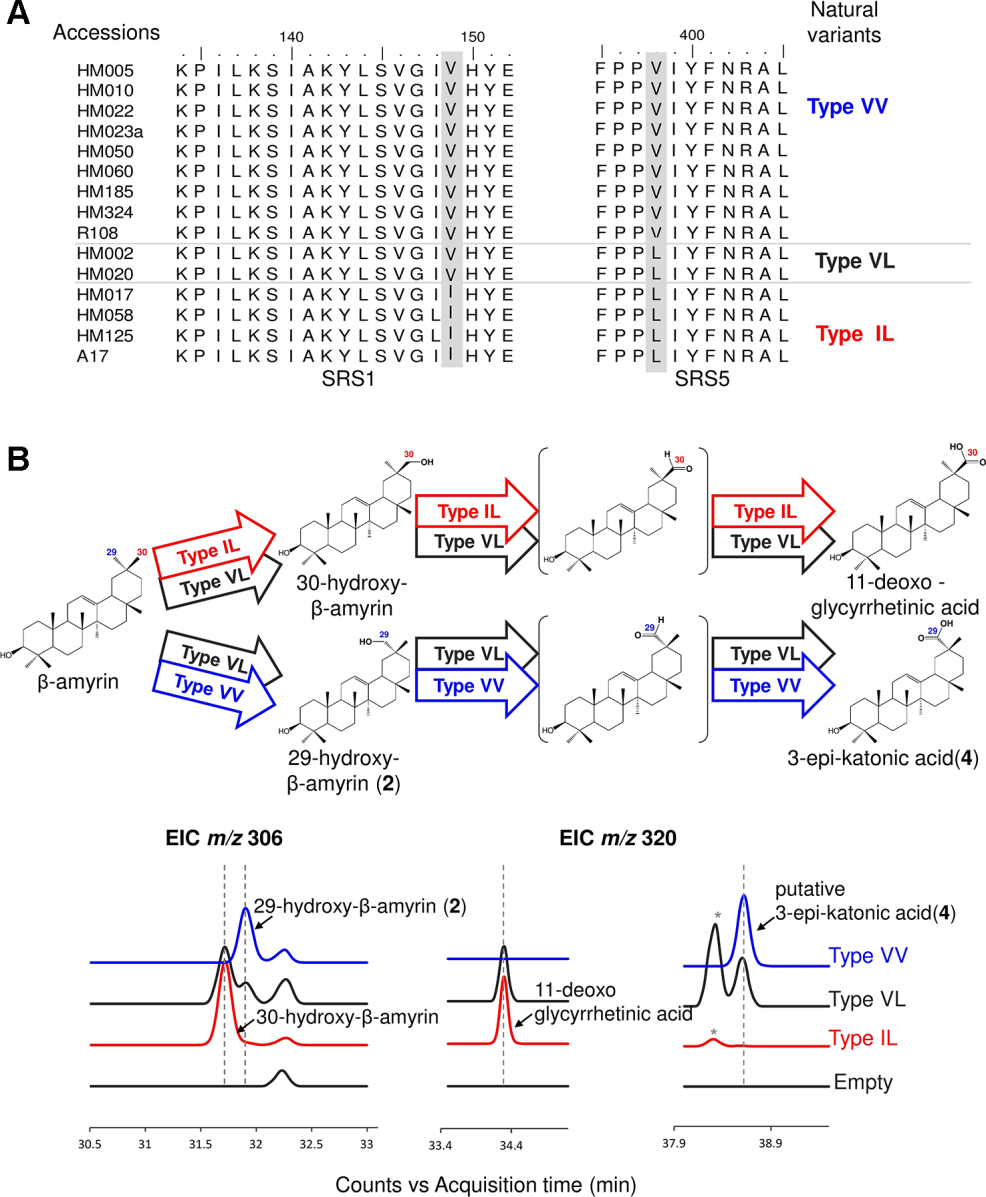
Divergent amino acid residues #149 and #398 showed different product regioselectivity. **(A)** Amino acid sequence alignment of CYP72A62v2 natural variants obtained by gene mining. Amino acids are shown in different colors to distinguish each variant. **(B)** *In vivo* enzymatic assay of CYP72A63 mutants mimicking CYP72A62 natural variants. Different colors indicate natural variant types. Identified products are labeled. The enzymatic activity of each variant is indicated by arrows with different colors. Peaks corresponding to unconfirmed β-amyrin are indicated with asterisks.

We used the *M. truncatula* Hapmap to further investigate the roles of residues #149 and #398 in product regioselectivity (Zhou et al., 2017). By focusing on amino acid residues #149 and #398, amino acid residue variants present in CYP72A62v2 were identified from *M. truncatula* accessions (Zhou et al., 2017) (**Figure 4A**). The variants were classified into three types based on differences in amino acid residues #149 and #398: Type VV (Val149, Val398), Type VL (Val149, Leu398), and Type IL (Ile149, Leu398). To evaluate the effects of divergent amino acid residues on product regioselectivity, we generated mutants of CYP72A63 mimicking each Types, VV, VL, and IL. *In vivo* enzymatic assay showed that these artificial mutant enzymes had differences in product regioselectivity (**Figure 4B**, **Supplementary Figure 1D**). Unexpectedly, Type IL oxidized at the C-30 position, producing 30-hydroxy-β-amyrin and 11-deoxoglycyrrhetinic acid, which resembled CYP72A63 rather than CYP72A62v2. These results clearly showed that amino acid residues #149 and #398 are essential for fine tuning of product regioselectivity.

### Residue #398 may be Involved in Generating Triterpene Chemodiversity in *Glycyrrhiza* Species

**Figure 5:**
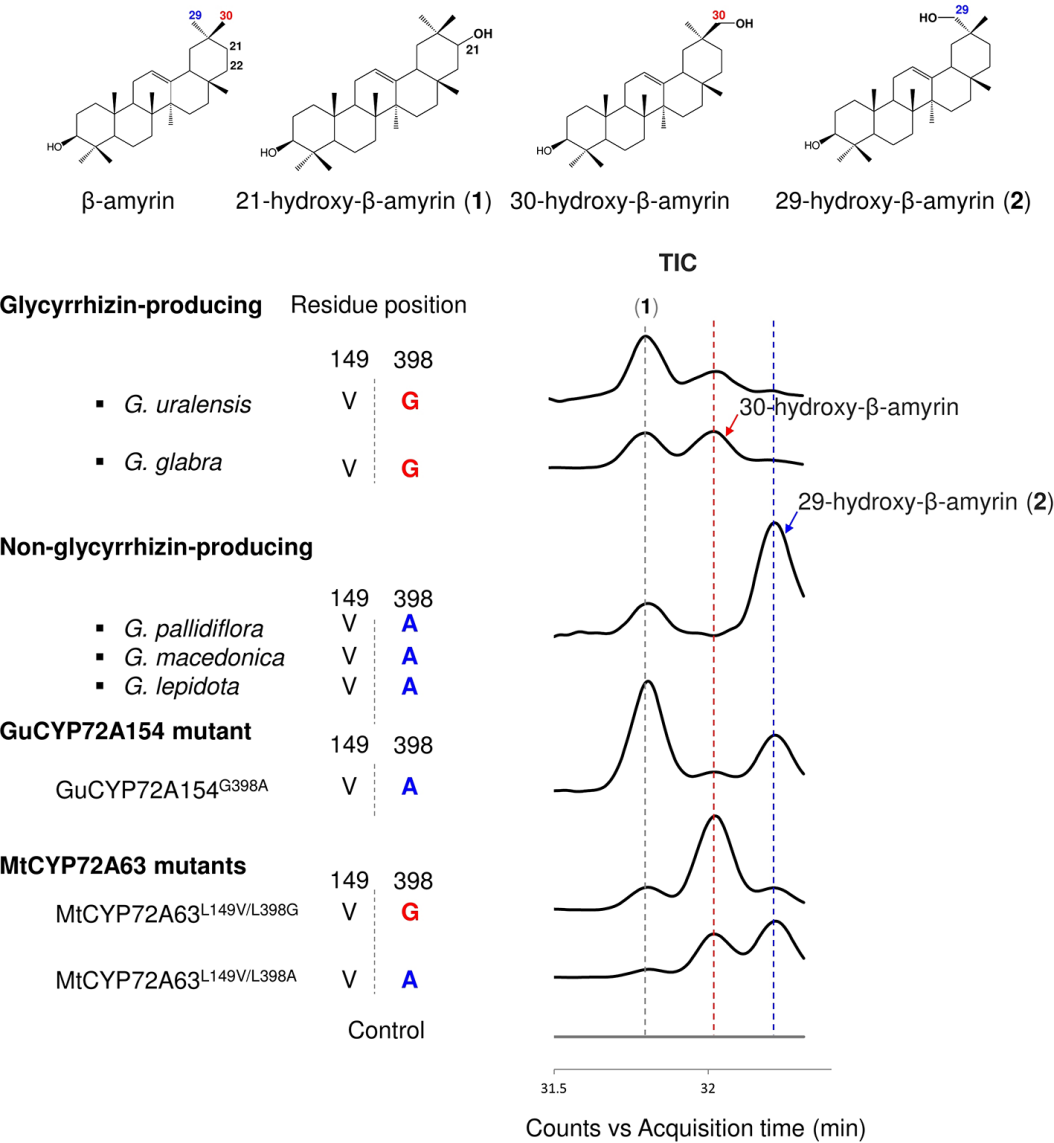
*In vivo* enzymatic assay of CYP72A154 variants and mutants from *Glycyrrhiza* species. Amino acid residues #149 and #398 are numbered based on the CYP72A63 amino acid sequence. Total ion current chromatograms are shown in enlargement mode. Identified peaks are indicated. Amino acid residue #398 and product regioselectivity are indicated in red and blue, for C-30 and C-29, respectively.

To investigate the roles of amino acid residues #149 and #398 in generating triterpene chemodiversity in *Glycyrrhiza* species, CYP72A154 variants from glycyrrhizin-producing species (*Glycyrrhiza uralensis*, *Gu*CYP72A154; *G. glabra*, *Gg*CYP72A154) and non-glycyrrhizin-producing species (*Glycyrrhiza pallidiflora*, *Gp*CYP72A154; *G. lepidota*, *Gl*CYP72A154; *G. macedonica*, *Gmac*CYP72A154) were investigated. As the full-length amino acid sequences of the three non-glycyrrhizin-producing species were identical, we selected *Gp*CYP72A154 as a representative species. Sequence alignment of CYP72A154 variants showed that amino acid residue #149 (numbering based on CYP72A63) is Val149 for both types, but amino acid residue #398 (numbering based on CYP72A63) differs between them, i.e., Gly398 for glycyrrhizin-producing species and Ala398 for non-glycyrrhizin-producing species (**Figure 5**). To characterize the role of divergent amino acid residue #398 in product regioselectivity in CYP72A154 variants, *in vivo* enzymatic assay was performed.*Gu*CYP72A154 and *Gg*CYP72A154 oxidized β-amyrin with less regioselectivity at the D or E ring, producing unknown compound 1 (peak 1) and 30-hydroxy-β-amyrin as the main products and 29-hydroxy-β-amyrin as a trace product (**Figure 5**, **Supplementary Figure 1E**). In contrast, the non-glycyrrhizin-producing species *Gp*CYP72A154 oxidized β-amyrin, producing unknown compound 1 (peak 1) and 29-hydroxy-β-amyrin as the main products and 30-hydroxy-β-amyrin as a trace product (**Figure 5**, **Supplementary Figure 1E**). These results suggested that the product regioselectivity of CYP72A154 variants differs between glycyrrhizin-producing species and non-glycyrrhizin-producing species.

To characterize the product regioselectivity of CYP72A154 variants, we also elucidated two unknown products in the residue remaining after purification of 30-hydroxy-11-oxo-β-amyrin from yeast co-expressing β-amyrin synthase, CYP88D6, *Gu*CYP72A154, and CPR (Peaks 4b and 4c in **Figure 2A** shown in Seki et al., 2011). NMR spectroscopy showed that the unknown compounds of 30-hydroxy-11-oxo-β-amyrin isomers were 29-hydroxy-11-oxo-β-amyrin and 21β-hydroxy-11-oxo-β-amyrin (**Supplementary Data**). These results showed that *Gu*CYP72A154 catalyzed oxidation at C-21β, C-29, and C-30.

To investigate whether the differences in product regioselectivity of CYP72A154 variants are associated with divergence of amino acid residue #398, we generated mutant *Gu*CYP72A154^G398A^ resembling *Gp*CYP72A154. *In vivo* enzymatic assay showed that product regioselectivity of *Gu*CYP72A154^G398A^ changed markedly, more closely resembling *Gp*CYP72A154 product regioselectivity and producing unknown compound 1 (peak 1, putative 21β-hydroxy-β-amyrin) and 29-hydroxy-β-amyrin as main products (**Figure 5**, **Supplementary Figure 1E**). In addition, we also generated mutants of CYP72A63, CYP72A63^L149V/L398G^ and CYP72A63^L149V/L398A^, carrying the amino acids at residues #149 and #398 in glycyrrhizin-producing species *Gu*CYP72A154 and non-glycyrrhizin-producing species *Gp*CYP72A154 (**Figure 5**, **Supplementary Figure 1E**). *In vivo* enzymatic assay of CYP72A63^L149V/L398G^ (mimicking glycyrrhizin-producing variants) showed oxidation activity mainly at the C-30 position, producing 30-hydroxy-β-amyrin as the main product, while CYP72A63^L149V/L398A^ (mimicking non-glycyrrhizin-producing variants) showed oxidation activity mainly at the C-29 position producing 29-hydroxy-β-amyrin as the main product. Mutagenesis of CYP72A63 mimicking CYP72A154 variants showed good agreement with the product regioselectivity of CYP72A154 variants from glycyrrhizin-producing and non-producing species. These results suggested that differences in amino acid residue #398 may be involved in generating triterpene chemodiversity in *Glycyrrhiza* species by conferring variable product regioselectivity.

### Divergent Amino Acid Residues #149 and #398 in Legume CYP72A63 Homologs

**Figure 6:**
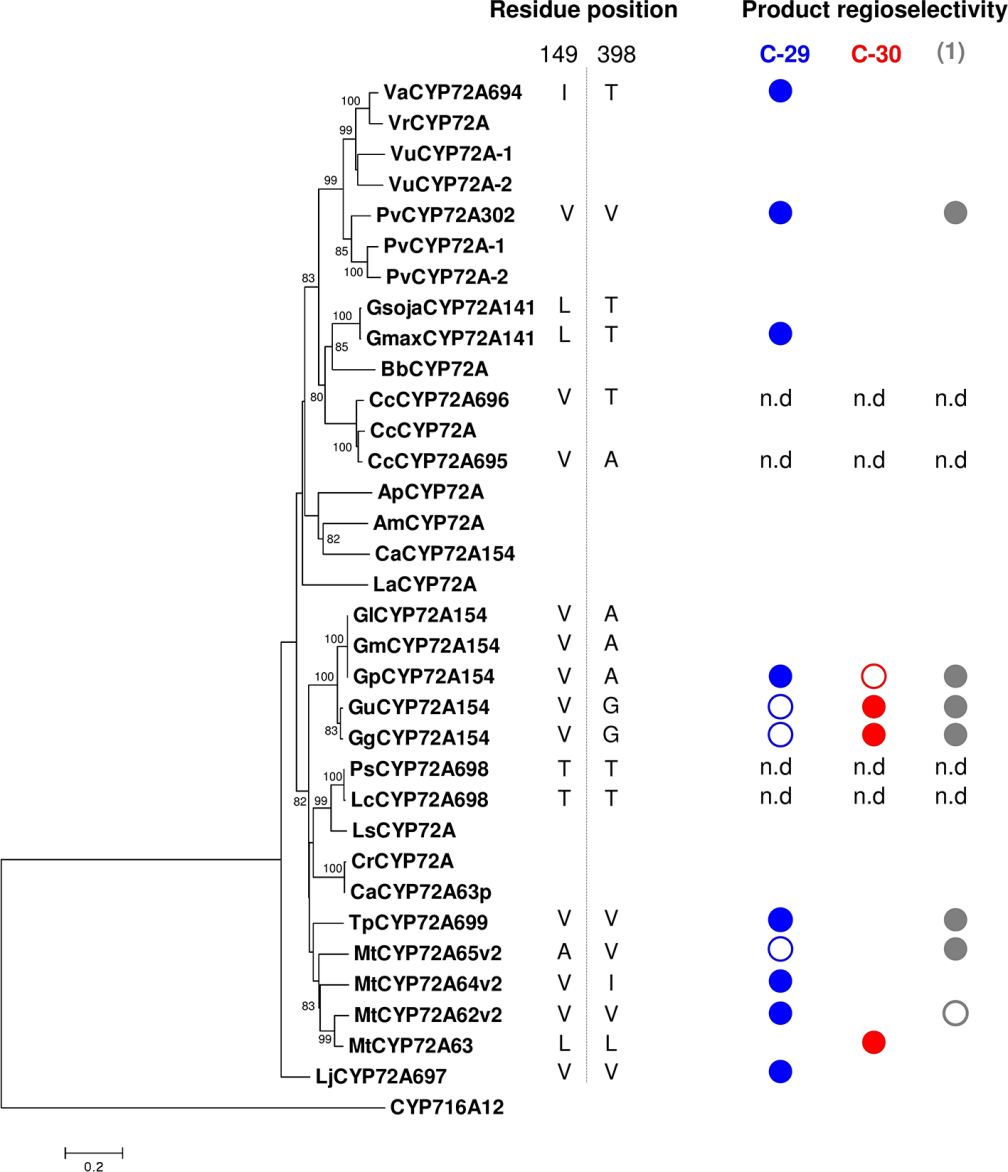
*In vivo* enzymatic activity of CYP72A63 homologs against β-amyrin as substrate. A phylogenetic tree was constructed using the amino acid sequence after removing membrane-bound sequences. Residues #149 and #398 are numbered according to the CYP72A63 amino acid sequence. Plant origins are abbreviated as follows: Va, *Vigna angularis*; Vr, *Vigna radiata*; Vu, *Vigna unguiculata*; Pv, *Phaseolus vulgaris*; Gmax, *Glycine max*; Gsoja, *Glycine soja*; Cc, *Cajanus cajan*; Gg, *Glycyrrhiza glabra*; Gu, *Glycyrrhiza uralensis*; Gl, *Glycyrrhiza lepidota*; Gp, *Glycyrrhiza pallidiflora*; Gm, *Glycyrrhiza macedonica*; Bb, *Bituminaria bituminosa*; Lc, *Lens culinaris*; Ps, *Pisum sativum*; Tp, *Trifolium pratense*; Mt, *Medicago truncatula*; Lj, *Lotus japonicus*; Ap, *Astragalus propinquus*; Am, *Astragalus membranaceus*; Ca, *Cicer arietinum*; La, *Lupinus angustifolius*; Ls, *Lathyrus sativus*. Only amino acid residues #149 and #398 from genes that were cloned and confirmed by DNA sequencing are shown. n.d., not detected (*in vivo* enzymatic assay in yeast did not show enzymatic activity). Full circles indicate the main product, while open circles indicate minor products. Regioselectivity on D or E ring [unknown compound **1** (peak 1), C-29, and C-30 are indicated in gray, blue, and red, respectively].

**Figure 7:**
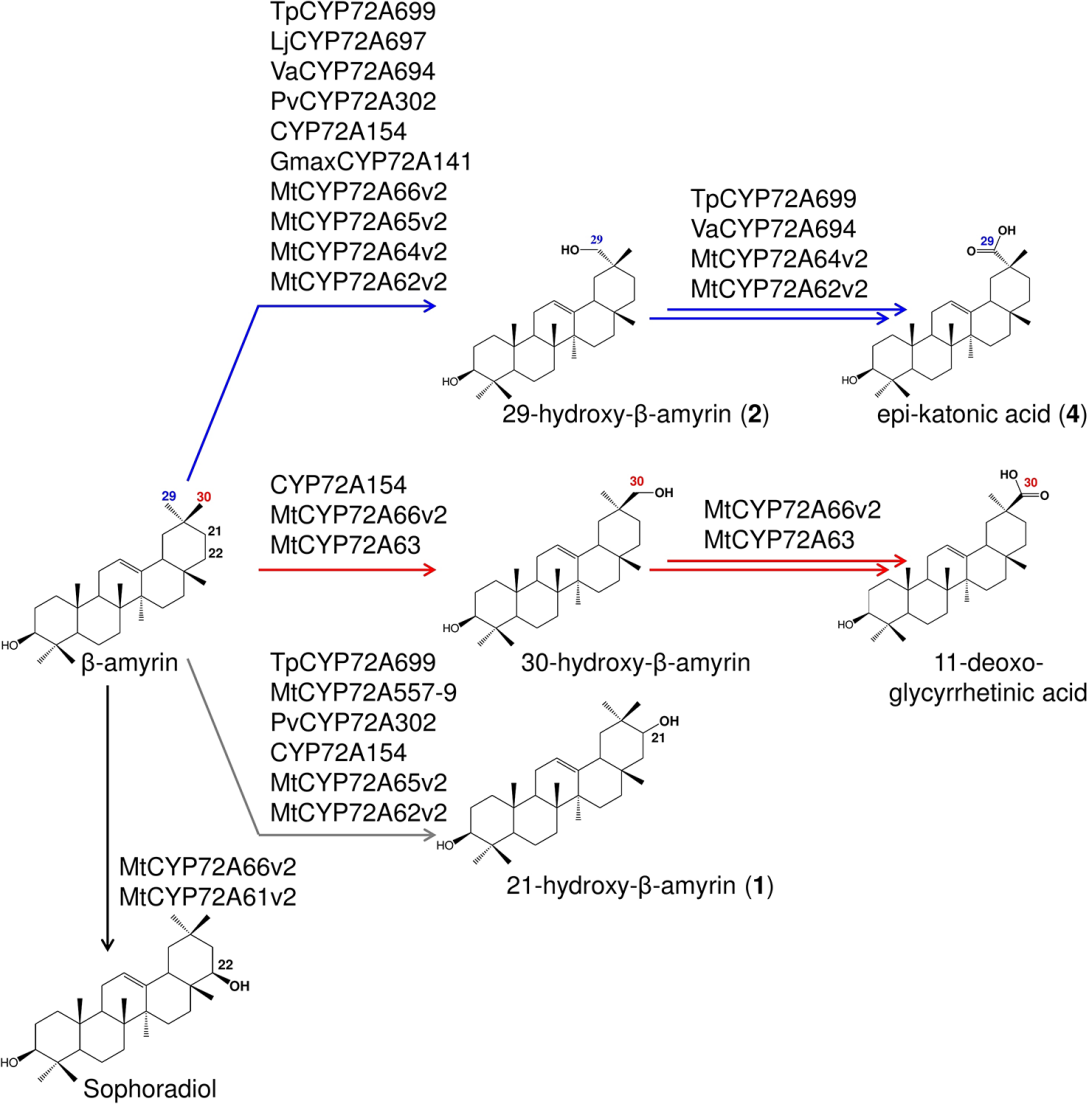
Summary of the regioselective oxidation activity of CYP72A subfamily enzymes reported in this study. Oxidation reactions on C-22β, putative C-21, C-29, and C-30 positions catalyzed by each P450s are indicated by arrows with different colors.

To further analyze the roles of amino acid residues #149 and #398 in CYP72A63 homologs from other legumes, we performed phylogenomic analyses of CYP72A63 homologs by constructing a phylogenetic tree, comparing amino acid residues #149 and #398, and performing *in vivo* enzymatic assays. CYP72A63 homologs were searched from publicly available genomic information and transcriptome databases of legume plants. Selected CYP72A63 homologs were cloned, confirmed by DNA sequencing, and submitted to the P450 Committee for naming (**Supplementary Table 3**). Phylogenetic analysis of CYP72A63 homologs showed that the legume species have a variable number of CYP72A63 homologs present in their genome (**Figure 6**); amino acid residues #149 and #398 varied among them. To investigate the relationships of amino acid residues #149 and #398 to product regioselectivity, *in vivo* enzymatic assays against β-amyrin were performed (**Figure 6**, **Supplementary Figure 1F**). None of the CYP72A63 homologs from these legumes showed C-30 oxidation activity, except CYP72A66v2 from *M. truncatula* and CYP72A154 from glycyrrhizin-producing *Glycyrrhiza* plants (**Figure 7**). The combinations of amino acid residues #149 and #398 differed among *Vigna angularis Va*CYP72A694 (Ile149, Thr398), *Glycine max Gmax* CYP72A141 (Leu149, Thr398), and *Lotus japonicus Lj*CYP72A697 (Val149, Val398), but *in vivo* enzymatic assays showed that they have regioselectivity in the C-29 position. *Trifolium pratense Tp*CYP72A699 and *Phaseolus vulgaris Pv*CYP72A302 have a combination of amino acid residues, Val149 and Val398, as seen in *Lotus japonicus Lj*CYP72A697, but *in vivo* enzymatic assay showed that they differed in product regioselectivity; however, regioselectivity in the C-29 position was common among them. These results support the important roles of amino acid residues #149 and #398 in determining regioselective oxidation activity.

## Discussion

In the absence of protein structures for P450s involved in triterpene biosynthesis, comprehensive analyses of the structure–function relationships could not be performed. Recent studies successfully demonstrated that comparative functional analyses of natural variants or evolutionarily related enzymes could be useful in narrowing down important amino acid residues, such as those involved in substrate and product specificity (Komori et al., 2013; Chen and Li, 2017; Xue et al., 2018). Elucidation of the molecular basis of product specificity and regioselectivity of the enzymes enables us to improve product specificity and produce desired compounds more efficiently. In this study, investigation of tandem duplicated CYP72A subfamily genes in *M. truncatula* identified two amino acid residues, Leu149 and Leu398, responsible for C-30 regioselective oxidation activity.

CYP72A subfamily genes were found in tandem arrays in the genome of *M. truncatula* (**Figure 1A**). The tandem array of CYP72A subfamily members is not a specific feature of *M. truncatula* because it was also found in other legumes (**Supplementary Figure 2**), and even in non-legumes, such as *Barbarea vulgaris* (Liu et al., 2019), *Oryza sativa*, and *Arabidopsis thaliana* (Saika et al., 2014). Recently, Liu et al. (2019)reported that *CYP72A552*, one of the CYP72A subfamily genes present in tandem arrays, is involved in hederagenin-based saponin biosynthesis in *Barbarea vulgaris*. Similarly, Saika et al. (2014)also reported that *CYP72A31*, one of the CYP72A subfamily genes present in tandem arrays, is involved in the mechanism of herbicide tolerance in rice. These observations indicated that genes present in tandem arrays may have different functions.

Gene duplication followed by subsequent mutations is a well-known mechanism by which genes gain new functions through neofunctionalization and escape from adaptive conflict (Panchy et al., 2016). In neofunctionalization, one copy of a duplicated gene maintains the original function, while the other copy gains a novel function by accumulation of mutations (Panchy et al., 2016). Although the reasons why members of the CYP72A subfamily are commonly found in tandem arrays are still unknown, these findings suggest a mechanism of functional diversification of CYP72A subfamily enzymes in plants. The existence of the CYP72A subfamily is not specific to legumes. However, its contribution to the synthesis of structurally diverse triterpenes has been reported almost exclusively in legumes, with the exception of the C-21 oxidase of *Avena* spp. (Leveau et al., 2019) and C-23 oxidase of *Kalopanax septemlobus* (Han et al., 2018) and *Barbarea vulgaris* (Liu et al., 2019).

CYP72A63 is located in the tandem array together with CYP72A65v2, CYP72A64v2, and CYP72A62v2 on chromosome 8. A previous report suggested that CYP72A65 also showed C-30 oxidation activity (Zhu et al., 2018). However, our *in vivo* enzymatic assay of CYP72A subfamily enzymes from *M. truncatula* clearly showed that CYP72A63 is the one enzyme that selectively oxidizes the C-30 position. Considering the chromosomal localization and phylogenetic tree topology, this tandem array likely evolved from a common ancestor with accumulation of mutations. Mutations can directly affect enzyme function, or can be silent. In the case of CYP72A64v2 and CYP72A62v2, substitution of Ile398 to Val398 did not alter the enzyme regioselectivity. However, substitution of Val149 to Leu149 and Ile/Val398 to Leu398 changed the product regioselectivity from C-29 toward the C-30 position. Thus, amino acid residues #149 and #398 determined the substrate orientation, which controlled its product regioselectivity.

The enzymes catalyzing β-amyrin at the C-29 position were identified for the first time in this study (**Figure 7**). Among the C-29 oxidases, *Va*CYP72A694 exhibited greater accumulation of the carboxylated product (putative C-29 carboxylated product; up to 70% product ratio; **Supplementary Figure 1F**). The C-29-derived saponins have been found in legume plants; adzukisaponins in *V. angularis* (Kitagawa et al., 1983) and macedonocides in *G. macedonica* (Hayashi et al., 2000), and some showed promise as high-value triterpenoids. Yoshikawa et al. (2002)identified albiziasaponin B, a triterpene saponin with a carboxyl group at C-29 of its aglycone moiety, from the Thai medicinal plant, *Albizia myriophylla* (Cha-em Thai), showing sweetness 600 times greater than sucrose. In Thai folk medicine, the stem of *A. myriophylla* has been used as a substitute for licorice due to its sweetness (Yoshikawa et al., 2002), and is one of the ingredients in traditional medicine used for treatment of diabetes (Neamsuvan et al., 2015). Thus, identification of C-29 oxidases provides a new genetic tool for production of high-value C-29-derived triterpenoids by synthetic biology.

*Glycyrrhiza* species were classified into two types according to the accumulation of glycyrrhizin, i.e., glycyrrhizin-producing and non-glycyrrhizin-producing species. Glycyrrhizin-producing species mainly show accumulation of C-30-derived saponins, while non-producing species accumulate C-29-derived saponins (Hayashi et al., 2000). The enzymatic activity of CYP72A154 variants showed good agreement with saponin accumulation in both types of *Glycyrrhiza* species. Comparison of amino acid residues #149 and #398 in CYP72A154 variants suggested that divergence in amino acid residue #398 may be involved in the generation triterpene chemodiversity in *Glycyrrhiza* species.

**Figure 8:**
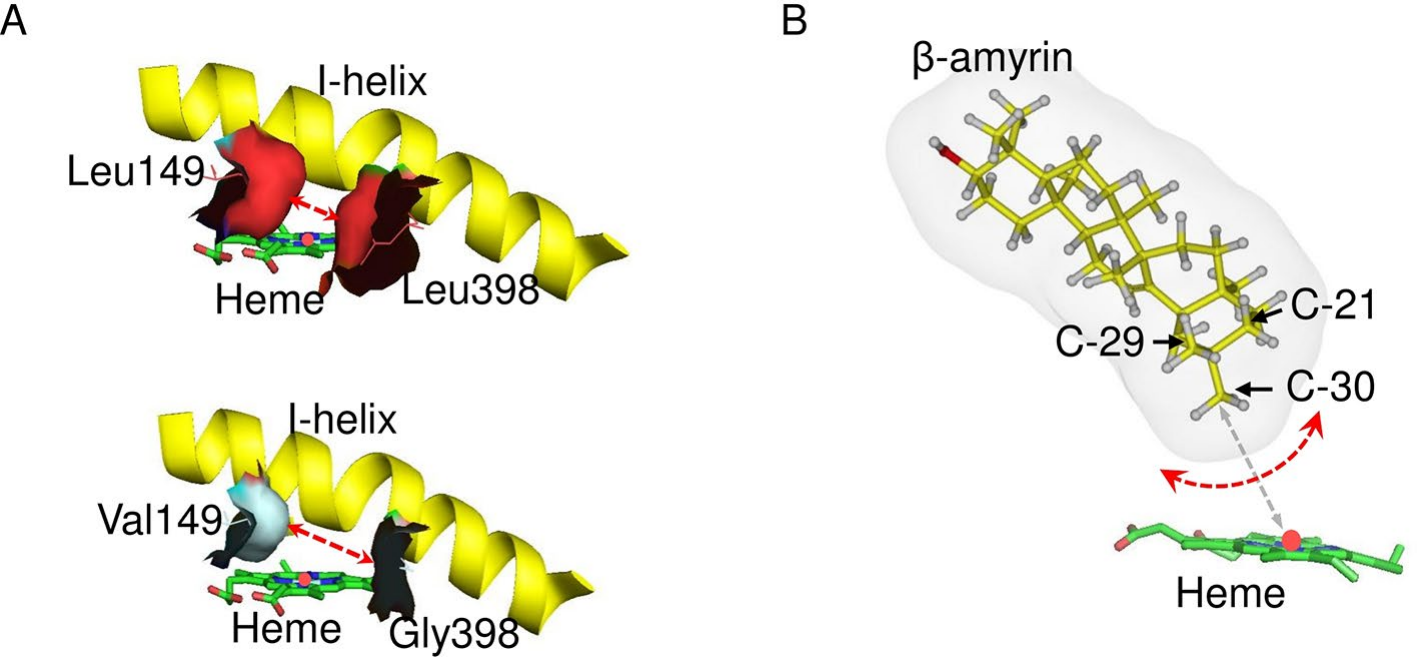
Model of the C-30 product regioselectivity. **(A)** *In silico* mutagenesis of CYP72A63^L149V/L398G^. These models show CYP72A63 and CYP72A63^L149V/L398G^ focusing on amino acid residues #149 and #398. The red dashed line indicates the effects of amino acid substitution on active site volume. All amino acid residues are shown. **(B)** Illustration of potential binding modes of β-amyrin. Carbon atom positions for C-21, C-29, and C-30 in the three-dimensional structure of β-amyrin are indicated. Potential rotation of β-amyrin is indicated by the red dashed line. The distance of C-30 from the heme iron is indicated by gray dashed line.

A substrate–enzyme complex model could not be obtained due to the low quality of the protein model. However, C-30 product regioselectivity was illustrated by *in silico* mutagenesis of CYP72A63 (**Figure 8**). Regioselectivity on C-30 and C-29 positions was controlled by amino acid residues #149 and #398, but the combinations of amino acid residues were different among them. The combination of amino acid residues with long nonpolar side chains (Ile/Leu149 and Leu398) resulted in C-30 product regioselectivity, while the combination of an amino acid residue with a nonpolar short side chain (Val149) and long nonpolar side chain (Ile149) resulted in C-29 product regioselectivity. Other combinations of amino acid residues with nonpolar short side chains (Val, Gly, Ala) at both positions resulted in broad regioselectivity, producing a number of isomers, i.e., C-30, C-29, and 21β. This suggested that C-30 product regioselectivity required amino acid residues with a nonpolar long side chain (Leu/Ile149 and Leu398) for specific placement of the methyl-30 group in the favorable position for enzyme reaction (**Figure 8**). Shortening the side chains of amino acid residues #149 and #398 increased the volume of the active site cavity, which allowed positioning of the substrate in multiple orientations (**Figure 8A**). Thus, C-30 product isomers could be produced by nonspecific positioning of methyl-29, methyl-30, and methylene-21 functional groups in proximity to mononuclear iron (**Figure 8B**). Interestingly, amino acid residues #149 and #398 were nonpolar amino acids (Ile, Leu, Val, Ala), except for Gly and Thr. This suggested that hydrophobic interactions may be important for positioning of the substrate in the most favorable orientation for enzyme catalysis. To explain the effects of 20 possible amino acid residues and the binding mode of the enzyme and substrate, mutagenesis studies and crystal structure analysis of CYP72A63 are required.

A recent study showed that CYP72A69 from soybean, *G. max*, catalyzed oxidation at the C-21β position in soyasapogenol A biosynthesis (Yano et al., 2017). *G. max* C-21 oxidase has a short side chain amino acid at residues #149 and #398 (i.e., Ala149 and Gly398, respectively; numbering based on CYP72A63). Similarly, *M. truncatula* CYP72A65v2, which catalyzed oxidation at putative C-21 (peak **1**), also has a short side chain amino acid at both residues #149 and #398 (i.e., Ala149 and Val398, respectively). The substitutions Leu149Ala and Leu398Val in CYP72A63 did not alter the product regioselectivity resembling CYP72A65v2 (data not shown), suggesting that C-21β product regioselectivity may be controlled by amino acid residues other than residues #149 and #398. The amino acid residues involved in product regioselectivity of CYP72A65v2 remain to be determined; doing so would lead to a better understanding of the evolutionary diversification of product regioselectivity of tandem array on chromosome 8.

Although we successfully altered the product regioselectivity of CYP72A62v2 from the C-29 position toward the C-30 position, by substituting Val149 to Leu149 and Val398 to Leu398, the mutant CYP72A62v2^V149L/V398L^ produced only trace amounts of C-30 carboxylated product (**Figure 3B**). However, the original enzyme, CYP72A63, produced the carboxylated product, suggesting that additional amino acid residues, other than #149 and #398, may be required for successive oxidation to produce the C-30 carboxylated product. Komori et al. (2013)showed that an amino acid residue located in the loop of SRS6, Ser479, in CYP71V1 is important for successive oxidation of amorpha-4,11-diene to produce carboxylated product (artemisinic acid) in the biosynthesis of the antimalarial sesquiterpenoid, artemisinin, in *Artemisia annua*. This suggested that the amino acid residues involved in C-30 carboxylated product specificity may also be located in SRS regions. Further investigations are required to identify the amino acid residues involved in C-30 successive oxidation to produce the C-30 carboxylated product.

We identified the key amino acid residues, Leu149 and Leu398, controlling C-30 product regioselectivity in CYP72A63. The results reported here will enable us to improve the product specificity and produce desired compounds more efficiently. Rational engineering of C-30 oxidase by site saturation mutagenesis amino acid residues #149 and #398 may be useful for fine tuning of the methyl-30 functional group to a favorable position for enzyme catalysis, to improve product specificity and production yield. Alternatively, the results presented here suggested that it may be possible to redirect the product regioselectivity of *Va*CYP72A694, a high carboxylated product producer (up to 70% accumulation), toward the C-30 position by rational protein engineering. The application of protein engineering in combination with metabolic engineering has been shown to significantly improve the production of natural products. Our findings will provide opportunities to further enhance the production of the valuable triterpene glycyrrhizin through rational protein engineering of C-30 oxidase.

## Data Availability Statement

The nucleotide sequences isolated in this study have been submitted to the GenBank at NCBI, ncbi.nlm.nih.gov/genbank/.

## Author Contributions

MF, EF, and SS designed experiments. MF, SS, JT, MI, HSu, KO, and HSe performed experiments. MF, EF, SS, KO, and HSe wrote the article. EF, KS, and TM supervised the research. All authors discussed the results and approved the article.

## Funding

This study was supported in part by the Grants-in-Aid for Scientific Research of the Japan Society for the Promotion of Science (JSPS) KAKENHI Grant Number JP19H02921; the Scientific Technique Research Promotion Program for Agriculture, Forestry, Fisheries, and Food Industry, Japan; The Program for Promotion of Basic and Applied Researches for Innovations in Bio-oriented Industry (BRAIN); The Special Fund from the Director of RIKEN Yokohama Institute; The RIKEN Rijicho Fund; and the Monbukagakusho Scholarship.

## Conflict of Interest

Authors HSu and SS were employed by company Tokiwa Phytochemical Co., Ltd.

The remaining authors declare that the research was conducted in the absence of any commercial or financial relationships that could be construed as a potential conflict of interest.
