# Supplementary material for: Molecular Basis of C-30 Product Regioselectivity of Legume Oxidases Involved in High-Value Triterpenoid Biosynthesis
Source: Front Plant Sci. 2019 Nov 26;10:1520. doi: 10.3389/fpls.2019.01520 (PMC6901910; doi:10.3389/fpls.2019.01520)
Supplement: Supplementary file 1 [file DataSheet_1.zip › 11-01-2019_10.3389-fpls.2019.01520/Supplementary Data.PDF]

Supplementary Data

Structural elucidation of compound 2 (peak 2) produced by yeast strain harboring β-amyrin synthase, CPR, and CYP72A63$^{L149V/L398V}$. To obtain compound 2, extract from yeast strain coexpressing β-amyrin synthase, CPR, and CYP72A63$^{L149V/L398V}$ was subjected to silica column chromatography affording about 8 mg of compound 2. NMR spectra were recorded on a Bruker Avance III 600 MHz spectrometer using $CDCl_3$ as the solvent. Complete ^{13}C assignment of purified compound was not achieved due to incomplete removal of impurities. However, the data indicated the presence of 29-hydroxy-β-amyrin. First, the mass spectrum of peak (2) was similar to that of trimethylsilylated 30-hydroxy-β-amyrin. These spectra showed molecular ions at *m*/*z* 586 and fragment ions at *m*/*z* 306 generated by retro Diels-Alder reaction in ring C (Budzikiewicz et al., 1963). These observations suggested that compound 2 should be monohydroxy-β-amyrin and the hydroxylated position should be in ring D or E of β-amyrin like 30-hydroxy-β-amyrin.

^{1}H NMR of compound 2 revealed the presence of seven methyl groups (0.79, 0.85, 0.91, 0.93, 0.97, 0.99, and 1.13 ppm) and two hydroxy groups. These results suggested that one of eight methyl groups of β-amyrin was hydroxylated. The DEPT135 data also indicated the presence of one hydroxymethyl group (δ_C = 79.0). On heteronuclear single quantum coherence (HSQC) spectroscopy, the ^{13}C chemical shift values of the seven methyl groups and two hydroxylated carbons were 15.6, 28.3, 19.0, 15.5, 16.8, 28.1, 26.0, 79.0 (δ_H = 3.22), and 74.8 (δ_H = 3.26) ppm, respectively. Heteronuclear multiple bond correlation (HMBC) experiments indicated that the hydroxymethyl group (δ_H/-δ_C = 3.26/74.8) and a methyl group (δ_H/-δ_C = 0.91/19.0) were correlated with each other, and also individually correlated with three common carbons; two secondary carbons (41.0 and 29.0 ppm) and one quaternary carbon (36.2 ppm). These observations suggested that the hydroxymethyl group and the methyl group should be attached to the same quaternary carbon (δ_C = 36.2). With the exception of C-4 in ring A, β-amyrin has only one quaternary carbon attached to two methyl groups, C-20 attached to Me-29 and Me-30. In conclusion, the hydroxylated position should be C-29 due to exclusion of C-30, based on direct comparison with the authentic sample of 30-hydroxy-β-amyrin. Therefore, compound 2 should be 29-hydroxy-β-amyrin.

Compound 2 (29-hydroxy-β-amyrin): ^{1}H NMR ($CDCl_3$, 600 MHz): δ_H 0.74 (1H, d, *J* = 11.3Hz), 0.79 (3H, s), 0.85 (3H, s), 0.91 (3H, s, Me-30), 0.93 (3H, s), 0.97 (3H, s), 0.99 (3H, s), 1.13 (3H, s), 3.22 (1H, dd, *J* = 11.4, 4.4 Hz, H-3), 3.26 (2H, s, H-29), 5.20 (1H, t, *J* = 3.7 Hz). ^{13}C NMR ($CDCl_3$, 150 MHz): δ_C 15.5, 15.6, 16.8, 18.4, 19.0 (C-30), 23.5, 26.0, 26.1, 26.9, 27.2, 28.1, 28.3, 29.0, 32.6, 32.9, 36.1, 36.2 (C-20), 36.9, 38.6, 38.8, 39.8, 41.0, 41.6, 46.3, 47.6, 55.1, 74.8 (C-29), 79.0 (C-3), 122.1, 144.8.

Structural elucidation of 30-hydroxy-11-oxo-β-amyrin isomers produced by yeast co-expressing β-amyrin synthase, CPR, CYP88D6, and CYP72A154. To purify the 30-hydroxy-11-oxo-β-amyrin isomers, 29-hydroxy-11-oxo-β-amyrin and 21β-hydroxy-11-oxo-β-amyrin, the residues remaining after purification of 30-hydroxy-11-oxo-β-amyrin from yeast co-expressing β-amyrin synthase, CYP88D6, CYP72A154, and CPR (Seki et al., 2011) were repeatedly subjected to reversed-phase high-performance liquid chromatography (HPLC) [column: Senshu Pak ODS (25 cm × 6.0 mm i.d.; Senshu Scientific Co., Ltd., Tokyo, Japan), solvent: 0.1% AcOH containing CH_3CN, flow rate, 1.5 mL/min]. Fractions containing 30-hydroxy-11-oxo-β-amyrin isomers were further purified by preparative silica gel TLC developed twice with hexane:ethyl acetate [1:1 (v/v)]. NMR spectra were recorded on an ECA-500 instrument (JEOL, Tokyo, Japan) in deuterated chloroform ($CDCl_3$) with tetramethylsilane as an internal standard.

29-hydroxy-11-oxo-β-amyrin: ^{1}H NMR ($CDCl_3$ with TMS, 500 MHz): δ_H 0.70 (1H, dd, *J* = 11.9, 1.6 Hz, H-5), 0.81 (3H, s, Me-24), 0.88 (3H, s, Me-28), 0.92 (3H, s, Me-30), 1.00 (3H, s,

Me-23), 1.14 (6H, s, Me-25 and Me-26), 1.36 (3H, s, Me-27), 2.05 (1H, td, *J* = 13.6, 4.4 Hz, 16α), 2.17 (1H, br dd, *J* = 13.3, 3.3 Hz, H-18), 2.33 (1H, s, H-9), 2.79 (1H, dt, *J* = 13.5, 3.6 Hz), 3.23 (1H, ddd, *J* = 10.0, 4.9, 4.9 Hz, H-3α), 3.28 (2H, br d, *J* = 4.6 Hz, H-29), 5.60 (1H, s, H-12). ^{13}C NMR ($CDCl_3$, 125 MHz): δ_C 15.6 (C-24), 16.4 (C-25[a]), 17.5 (C-6), 18.7 (C-26[a]), 19.0 (C-30), 23.5 (C-27), 26.38 (C-15[b]), 26.42 (C-16[b]), 27.3 (C-2), 28.1 (C-23), 28.6 (C-21), 28.7 (C-28), 32.8 (C-7[c] and C-17), 35.6 (C-22), 36.1 (C-20), 37.1 (C-10), 39.1 (C-1[c] and C-4), 39.5 (C-19), 43.3 (C-14), 45.4 (C-8), 46.7 (C-18), 54.9 (C-5), 61.8 (C-9), 74.2 (C-29), 78.8 (C-3), 128.3 (C-12), 170.1 (C-13), 200.2 (C-11). [a,b,c]These assignments may be interchanged.
21β-hydroxy-11-oxo-β-amyrin: ^{1}H NMR ($CDCl_3$ with TMS, 500 MHz): δ_H 0.70 (1H, dd, *J* = 11.9, 1.6 Hz, H-5), 0.81 (3H, s, Me-24), 0.88 (3H, s, Me-30), 0.90 (3H, s, Me-28), 0.99 (3H, s, Me-29), 1.00 (3H, s, Me-23), 1.12 (3H, s, Me-26), 1.13 (3H, s, Me-25), 1.35 (3H, s, Me-27), 2.02 (1H, td, *J* = 13.7, 4.9 Hz, 16α), 2.17 (1H, br dd, *J* = 13.8, 3.4 Hz, H-18), 2.33 (1H, s, H-9), 2.79 (1H, dt, *J* = 13.5, 3.6 Hz, H-1β), 3.22 (1H, dd, *J* = 10.9, 5.4 Hz, H-3α), 3.54 (1H, dd, *J* = 12.0, 4.6 Hz, H-21α), 5.60 (1H, s, H-12). ^{13}C NMR ($CDCl_3$, 125 MHz): δ_C 15.6 (C-24), 16.4 (C-25), 16.8 (C-30), 17.5 (C-6), 18.7 (C-26), 23.3 (C-27), 26.4 (C-15), 27.3 (C-2), 27.7 (C-16), 28.1 (C-23), 28.6 (C-28), 28.8 (C-29), 32.8 (C-7), 34.8 (C-17), 36.3 (C-20), 37.1 (C-10), 39.1 (C-1 and C-4), 43.3 (C-14), 44.7 (C-22), 45.3 (C-8), 45.4 (C-19), 46.9 (C-18), 54.9 (C-5), 61.8 (C-9), 73.6 (C-21), 78.8 (C-3), 128.6 (C-12), 168.9 (C-13), 200.1 (C-11).

References

Budzikiewicz, H., Wilson, J. M., and Djerassi, C. (1963). Mass spectrometry in structural and stereochemical problems. XXXII. Pentacyclic triterpenes. *J. Am. Chem. Soc.* 85, 3688–3699. doi: 10.1021/ja00905a036

Seki, H., Sawai, S., Ohyama, K., Mizutani, M., Ohnishi, T., Sudo, H., et al. (2011). Triterpene functional genomics in licorice for identification of CYP72A154 involved in the biosynthesis of glycyrrhizin. *Plant Cell*. 23, 4112–4123. doi: 10.1105/tpc.110.082685
