# Supplementary material for: Molecular Basis of C-30 Product Regioselectivity of Legume Oxidases Involved in High-Value Triterpenoid Biosynthesis
Source: Front Plant Sci. 2019 Nov 26;10:1520. doi: 10.3389/fpls.2019.01520 (PMC6901910; doi:10.3389/fpls.2019.01520)
Supplement: Supplementary file 1 [file DataSheet_1.zip › 11-01-2019_10.3389-fpls.2019.01520/Supplementary Figure S1.PDF]

(A)

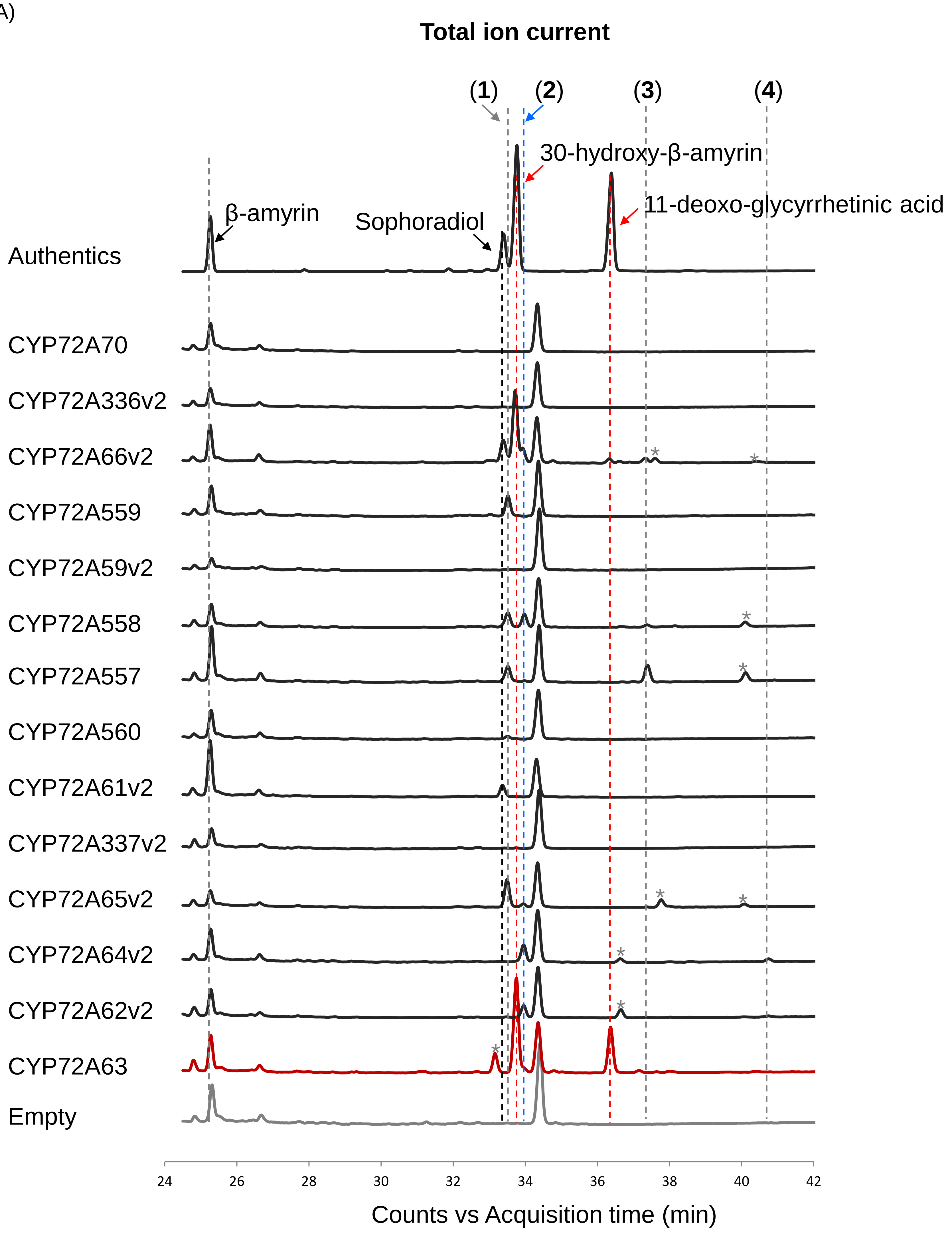

Total ion current
(1)
(2)
(3)
(4)
30-hydroxy-β-amyrin
11-deoxo-glycyrrhetinic acid
β-amyrin
Sophoradiol
Authentics
CYP72A70
CYP72A336v2
CYP72A66v2
CYP72A559
CYP72A59v2
CYP72A558
CYP72A557
CYP72A560
CYP72A61v2
CYP72A337v2
CYP72A65v2
CYP72A64v2
CYP72A62v2
CYP72A63
Empty
24
26
28
30
32
34
36
38
40
42
Counts vs Acquisition time (min)

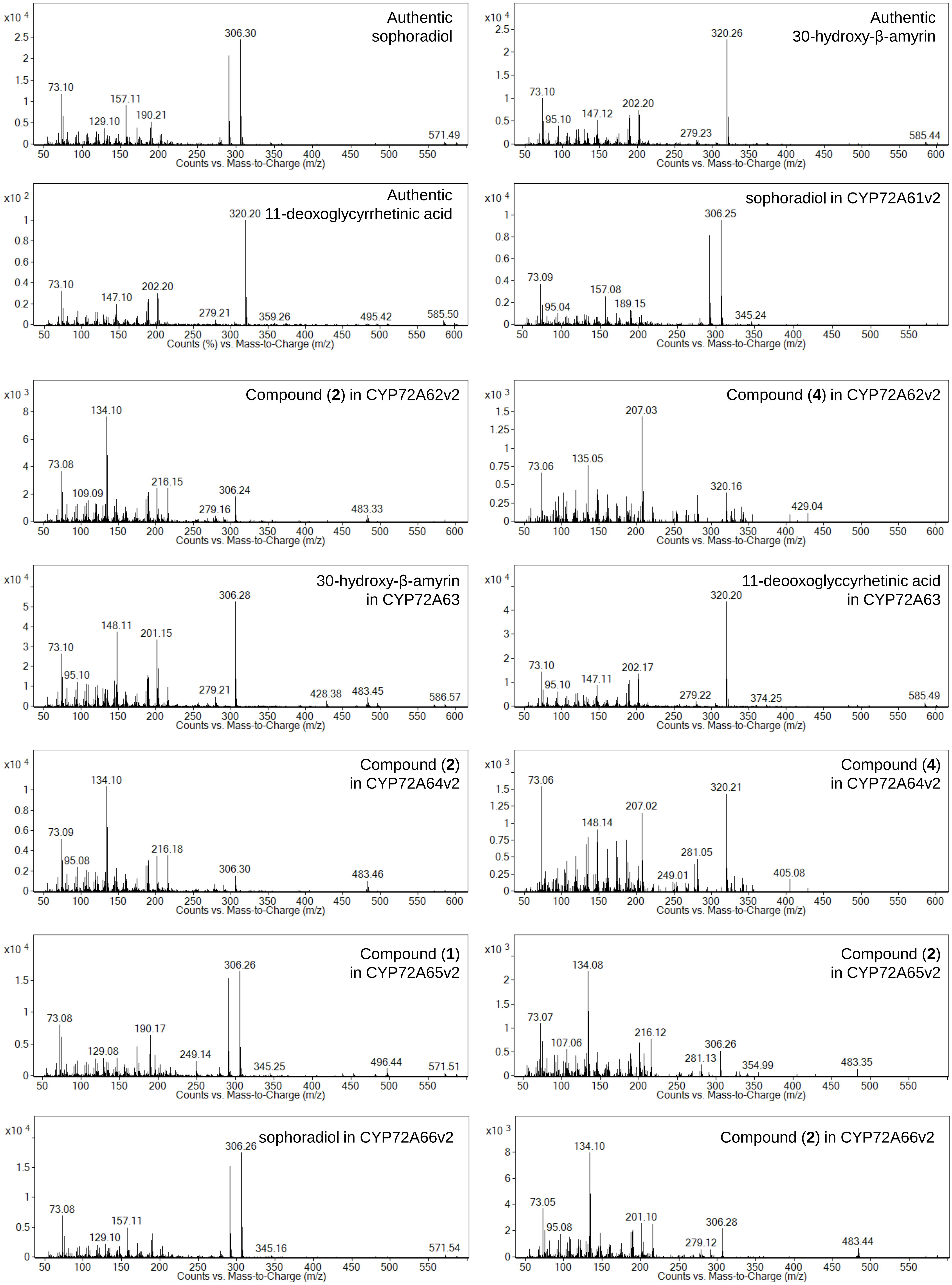
Authentic sophoradiol
x10 4
306.30
73.10
157.11
129.10
190.21
571.49
Counts vs. Mass-to-Charge (m/z)
Authentic 30-hydroxy-β-amyrin
x10 4
320.26
73.10
95.10
147.12
202.20
279.23
585.44
Counts vs. Mass-to-Charge (m/z)
Authentic 11-deoxoglycyrrhetinic acid
x10 2
320.20
73.10
147.10
202.20
279.21
359.26
495.42
585.50
Counts (%) vs. Mass-to-Charge (m/z)
sophoradiol in CYP72A61v2
x10 4
306.25
73.09
95.04
157.08
189.15
345.24
Counts vs. Mass-to-Charge (m/z)
Compound (2) in CYP72A62v2
x10 3
134.10
73.08
109.09
216.15
306.24
279.16
483.33
Counts vs. Mass-to-Charge (m/z)
Compound (4) in CYP72A62v2
x10 3
207.03
73.06
135.05
320.16
429.04
Counts vs. Mass-to-Charge (m/z)
30-hydroxy-β-amyrin in CYP72A63
x10 4
306.28
148.11
201.15
73.10
95.10
279.21
428.38
483.45
586.57
Counts vs. Mass-to-Charge (m/z)
11-deooxoglyccyrhetinic acid in CYP72A63
x10 4
320.20
73.10
95.10
147.11
202.17
279.22
374.25
585.49
Counts vs. Mass-to-Charge (m/z)
Compound (2) in CYP72A64v2
x10 4
134.10
73.09
95.08
216.18
306.30
483.46
Counts vs. Mass-to-Charge (m/z)
Compound (4) in CYP72A64v2
x10 3
73.06
320.21
207.02
148.14
281.05
249.01
405.08
Counts vs. Mass-to-Charge (m/z)
Compound (1) in CYP72A65v2
x10 4
306.26
73.08
190.17
129.08
249.14
345.25
496.44
571.51
Counts vs. Mass-to-Charge (m/z)
Compound (2) in CYP72A65v2
x10 3
134.08
73.07
107.06
216.12
306.26
281.13
354.99
483.35
Counts vs. Mass-to-Charge (m/z)
sophoradiol in CYP72A66v2
x10 4
306.26
73.08
157.11
129.10
345.16
571.54
Counts vs. Mass-to-Charge (m/z)
Compound (2) in CYP72A66v2
x10 3
134.10
73.05
95.08
201.10
306.28
279.12
483.44
Counts vs. Mass-to-Charge (m/z)

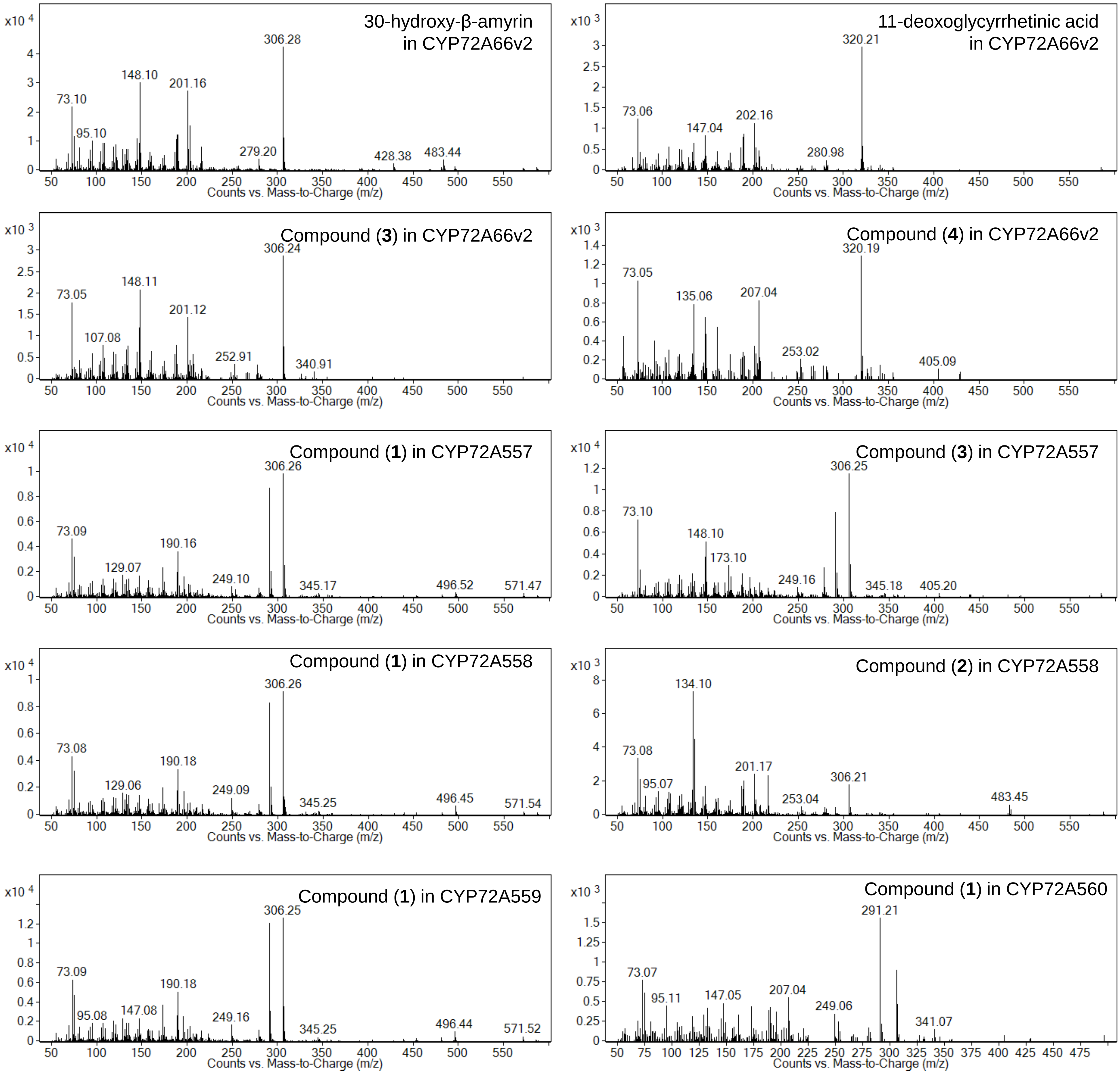
30-hydroxy-β-amyrin in CYP72A66v2
x10 4
73.10
95.10
148.10
201.16
279.20
306.28
428.38
483.44
11-deoxoglycyrrhetinic acid in CYP72A66v2
x10 3
73.06
147.04
202.16
280.98
320.21
Compound (3) in CYP72A66v2
x10 3
73.05
107.08
148.11
201.12
252.91
306.24
340.91
Compound (4) in CYP72A66v2
x10 3
73.05
135.06
207.04
253.02
320.19
405.09
Compound (1) in CYP72A557
x10 4
73.09
129.07
190.16
249.10
306.26
345.17
496.52
571.47
Compound (3) in CYP72A557
x10 4
73.10
148.10
173.10
249.16
306.25
345.18
405.20
Compound (1) in CYP72A558
x10 4
73.08
129.06
190.18
249.09
306.26
345.25
496.45
571.54
Compound (2) in CYP72A558
x10 3
73.08
95.07
134.10
201.17
253.04
306.21
483.45
Compound (1) in CYP72A559
x10 4
73.09
95.08
147.08
190.18
249.16
306.25
345.25
496.44
571.52
Compound (1) in CYP72A560
x10 3
73.07
95.11
147.05
207.04
249.06
291.21
341.07
Counts vs. Mass-to-Charge (m/z)

(B)

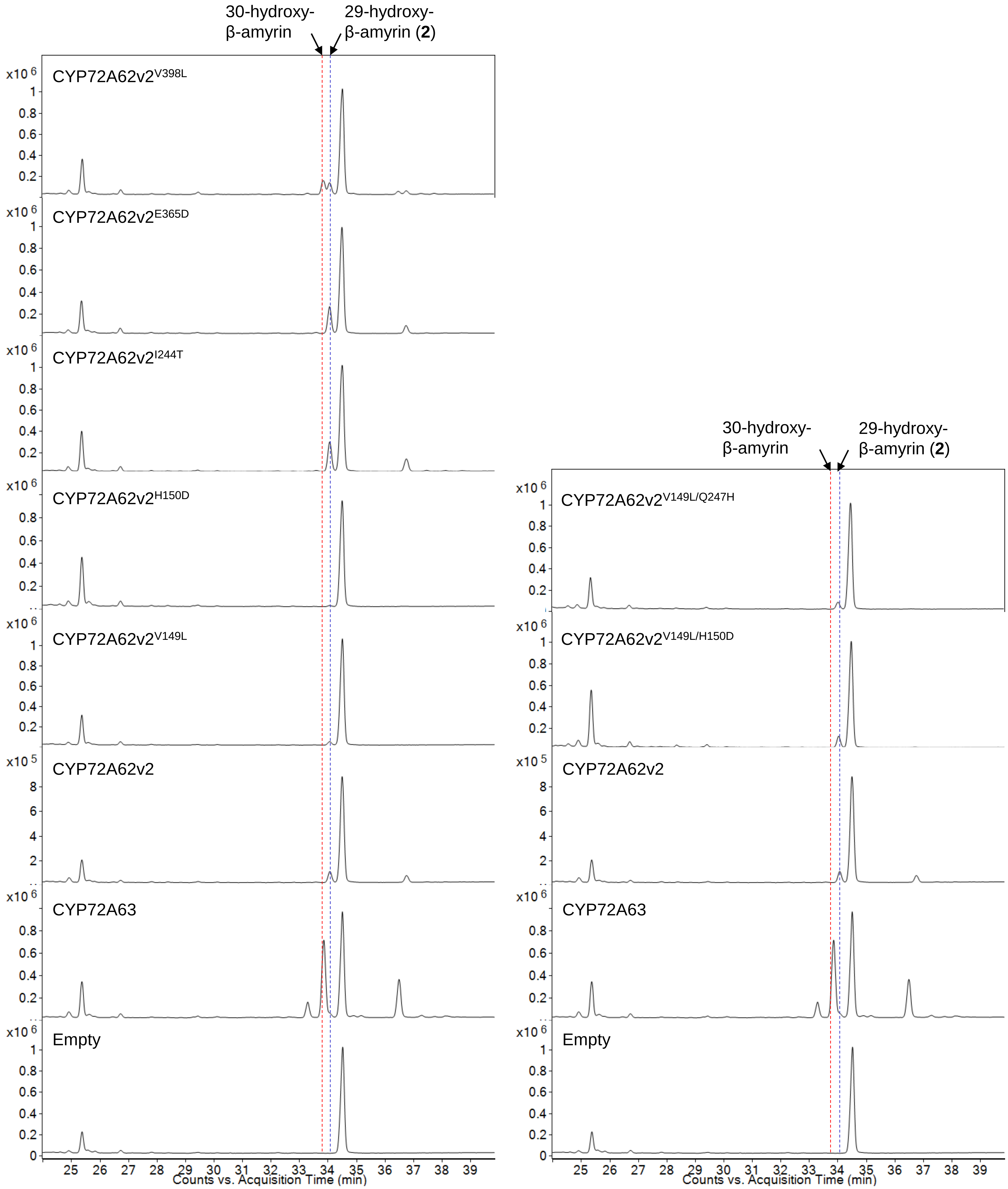

30-hydroxy-β-amyrin
29-hydroxy-β-amyrin (2)
CYP72A62v2V398L
CYP72A62v2E365D
CYP72A62v2I244T
CYP72A62v2H150D
CYP72A62v2V149L
CYP72A62v2
CYP72A63
Empty
CYP72A62v2V149L/Q247H
CYP72A62v2V149L/H150D
Counts vs. Acquisition Time (min)

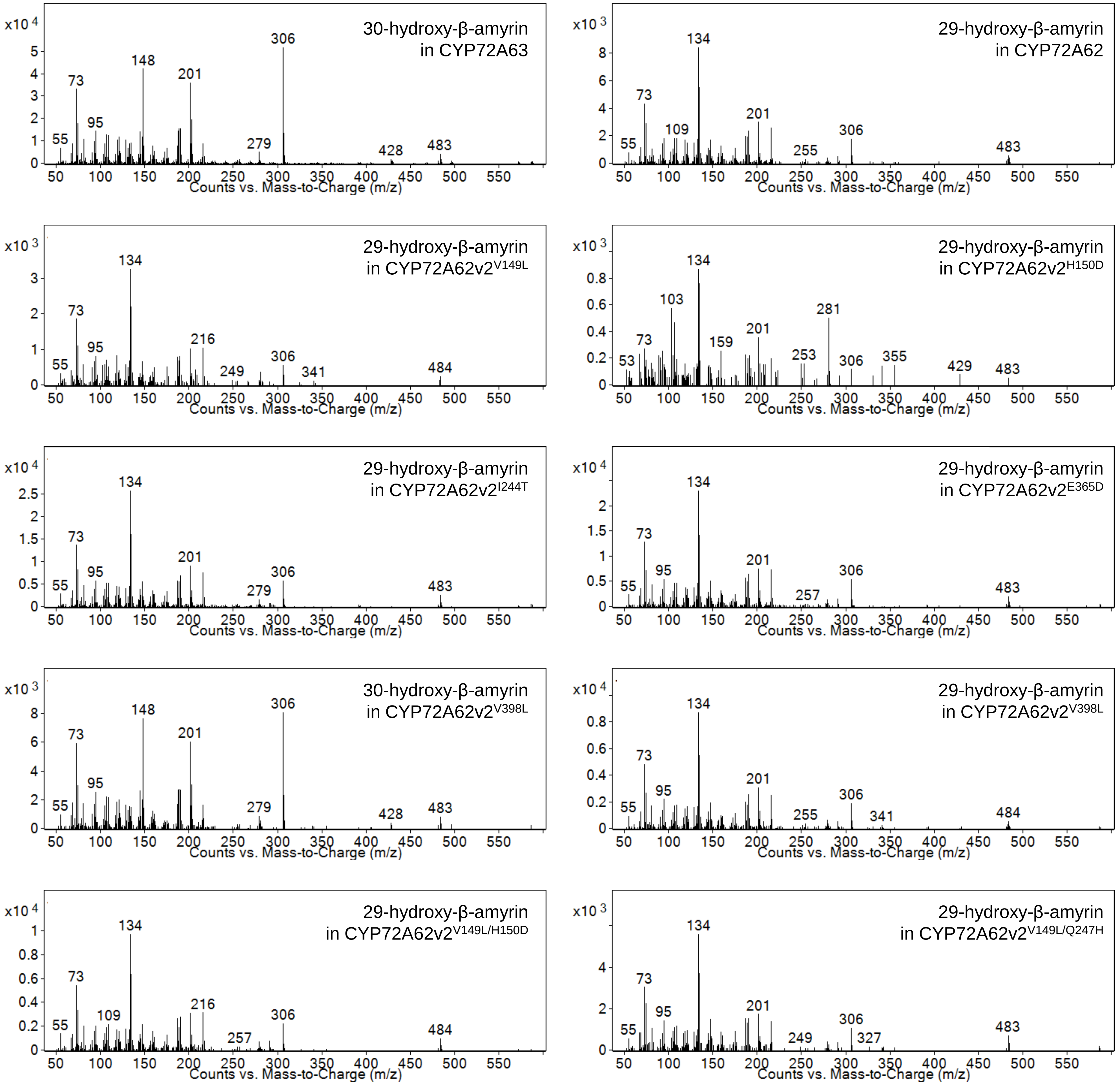
x10 4
30-hydroxy-β-amyrin in CYP72A63
55 73 95 148 201 279 306 428 483
Counts vs. Mass-to-Charge (m/z)
x10 3
29-hydroxy-β-amyrin in CYP72A62
55 73 109 134 201 255 306 483
Counts vs. Mass-to-Charge (m/z)
x10 3
29-hydroxy-β-amyrin in CYP72A62v2V149L
55 73 95 134 216 249 306 341 484
Counts vs. Mass-to-Charge (m/z)
x10 3
29-hydroxy-β-amyrin in CYP72A62v2H150D
53 73 103 134 159 201 253 281 306 355 429 483
Counts vs. Mass-to-Charge (m/z)
x10 4
29-hydroxy-β-amyrin in CYP72A62v2I244T
55 73 95 134 201 279 306 483
Counts vs. Mass-to-Charge (m/z)
x10 4
29-hydroxy-β-amyrin in CYP72A62v2E365D
55 73 95 134 201 257 306 483
Counts vs. Mass-to-Charge (m/z)
x10 3
30-hydroxy-β-amyrin in CYP72A62v2V398L
55 73 95 148 201 279 306 428 483
Counts vs. Mass-to-Charge (m/z)
x10 4
29-hydroxy-β-amyrin in CYP72A62v2V398L
55 73 95 134 201 255 306 341 484
Counts vs. Mass-to-Charge (m/z)
x10 4
29-hydroxy-β-amyrin in CYP72A62v2V149L/H150D
55 73 109 134 216 257 306 484
Counts vs. Mass-to-Charge (m/z)
x10 3
29-hydroxy-β-amyrin in CYP72A62v2V149L/Q247H
55 73 95 134 201 249 306 327 483
Counts vs. Mass-to-Charge (m/z)

(C)

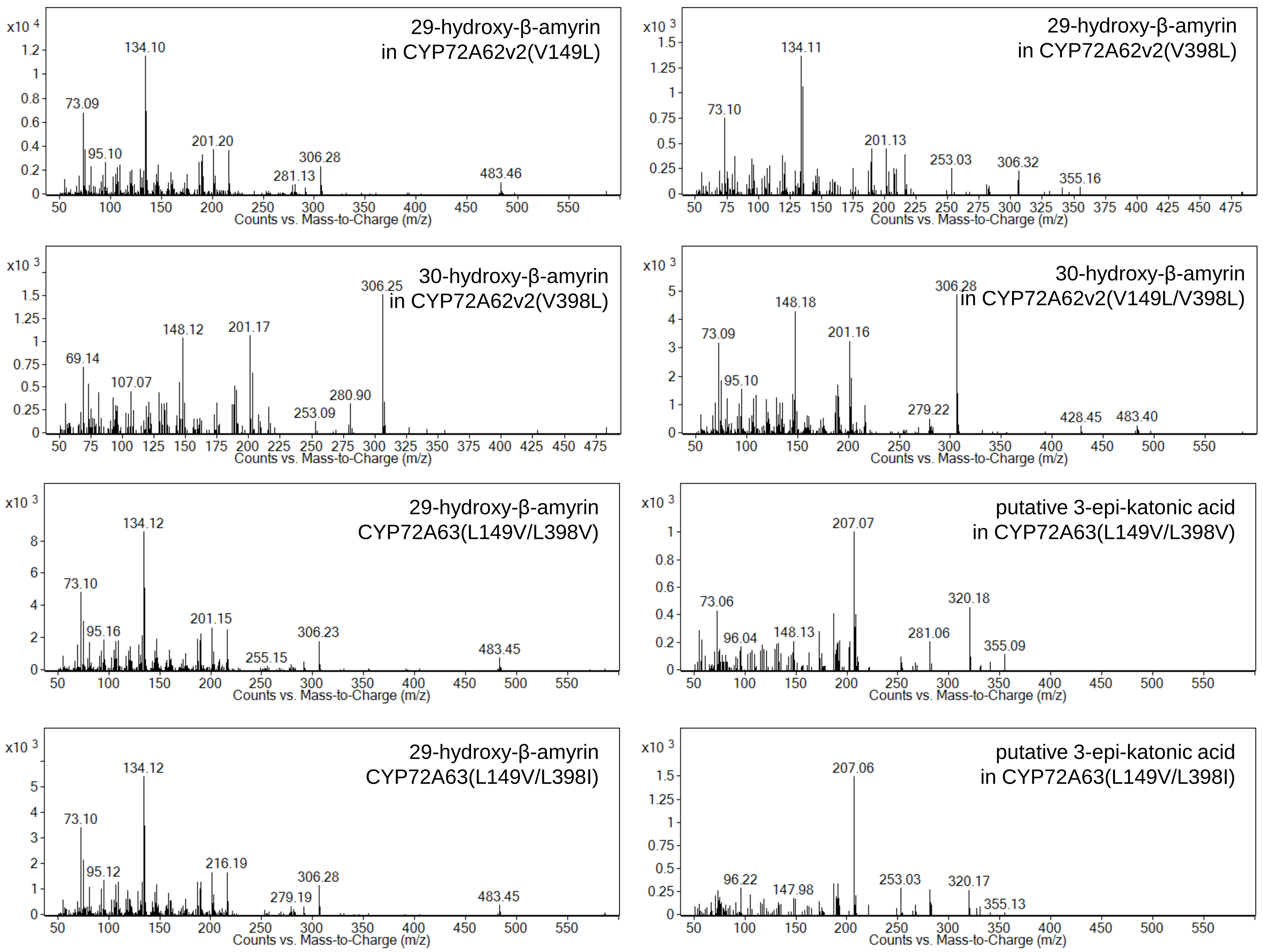
x10 4
29-hydroxy-β-amyrin
in CYP72A62v2(V149L)
134.10
73.09
95.10
201.20
306.28
281.13
483.46
Counts vs. Mass-to-Charge (m/z)
x10 3
29-hydroxy-β-amyrin
in CYP72A62v2(V398L)
134.11
73.10
201.13
253.03
306.32
355.16
Counts vs. Mass-to-Charge (m/z)
x10 3
30-hydroxy-β-amyrin
in CYP72A62v2(V398L)
306.25
148.12
201.17
69.14
107.07
280.90
253.09
Counts vs. Mass-to-Charge (m/z)
x10 3
30-hydroxy-β-amyrin
in CYP72A62v2(V149L/V398L)
306.28
148.18
73.09
201.16
95.10
279.22
428.45
483.40
Counts vs. Mass-to-Charge (m/z)
x10 3
29-hydroxy-β-amyrin
CYP72A63(L149V/L398V)
134.12
73.10
201.15
95.16
306.23
255.15
483.45
Counts vs. Mass-to-Charge (m/z)
x10 3
putative 3-epi-katonic acid
in CYP72A63(L149V/L398V)
207.07
73.06
320.18
96.04
148.13
281.06
355.09
Counts vs. Mass-to-Charge (m/z)
x10 3
29-hydroxy-β-amyrin
CYP72A63(L149V/L398I)
134.12
73.10
216.19
95.12
306.28
279.19
483.45
Counts vs. Mass-to-Charge (m/z)
x10 3
putative 3-epi-katonic acid
in CYP72A63(L149V/L398I)
207.06
96.22
147.98
253.03
320.17
355.13
Counts vs. Mass-to-Charge (m/z)

(D)

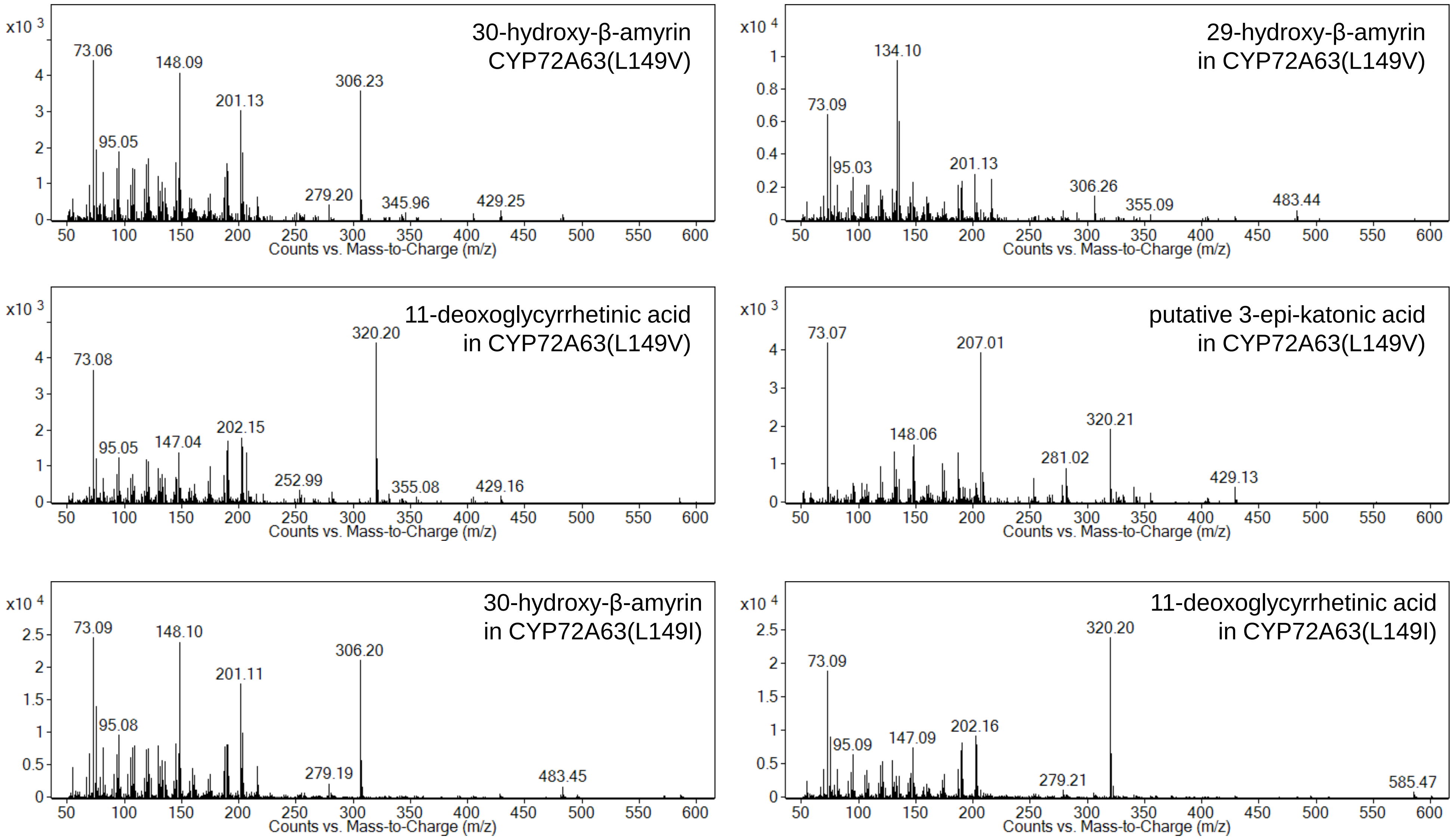
30-hydroxy-β-amyrin
CYP72A63(L149V)
x10 3
73.06
148.09
306.23
201.13
95.05
279.20
345.96
429.25
29-hydroxy-β-amyrin
in CYP72A63(L149V)
x10 4
134.10
73.09
95.03
201.13
306.26
355.09
483.44
11-deoxoglycyrrhetinic acid
in CYP72A63(L149V)
x10 3
320.20
73.08
95.05
147.04
202.15
252.99
355.08
429.16
putative 3-epi-katonic acid
in CYP72A63(L149V)
x10 3
73.07
207.01
320.21
148.06
281.02
429.13
30-hydroxy-β-amyrin
in CYP72A63(L149I)
x10 4
73.09
148.10
306.20
201.11
95.08
279.19
483.45
11-deoxoglycyrrhetinic acid
in CYP72A63(L149I)
x10 4
320.20
73.09
202.16
147.09
95.09
279.21
585.47
Counts vs. Mass-to-Charge (m/z)

(E)

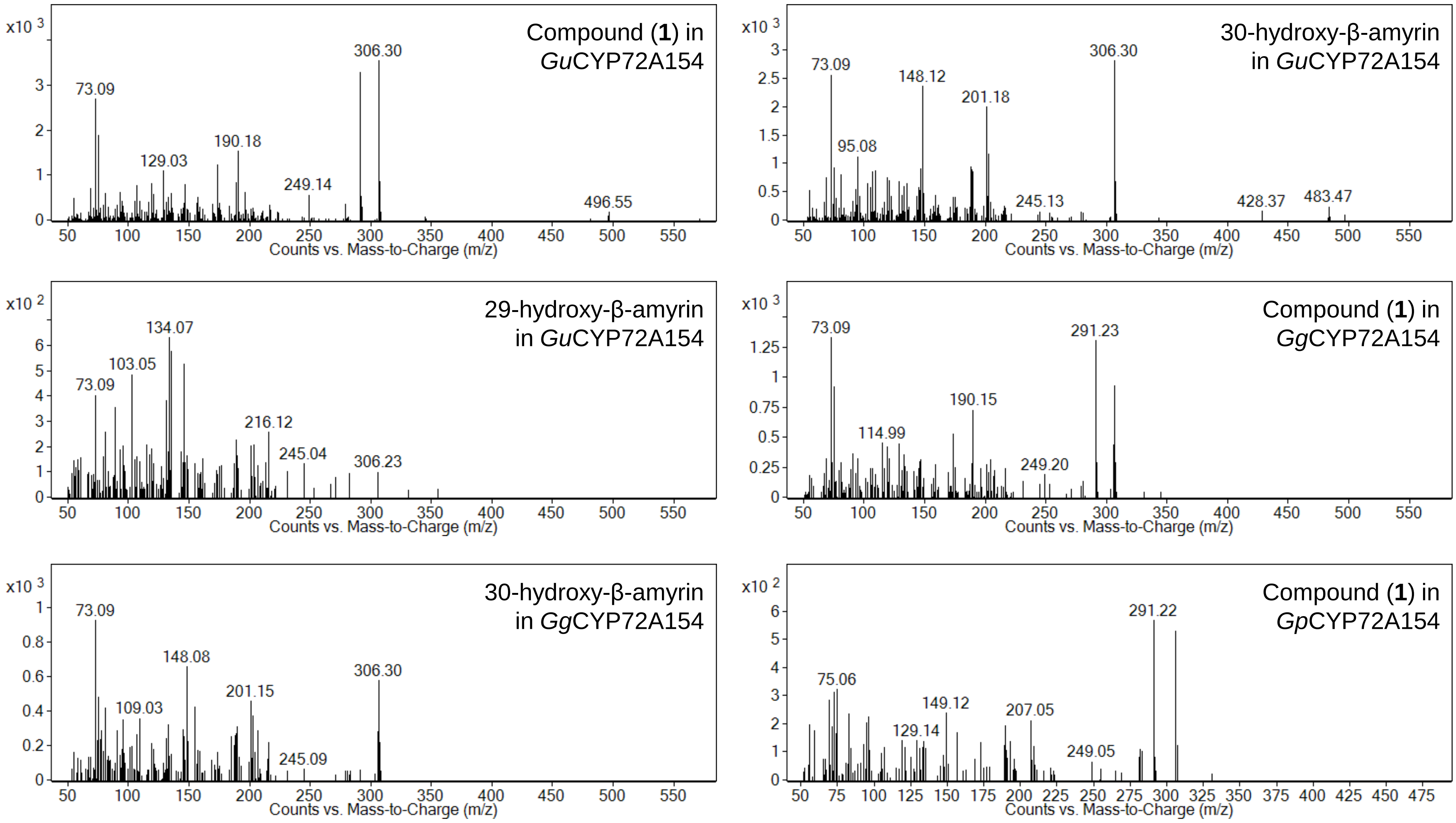
Compound (1) in
GuCYP72A154
x10 3
306.30
73.09
190.18
129.03
249.14
496.55
30-hydroxy-β-amyrin
in GuCYP72A154
x10 3
306.30
73.09
148.12
201.18
95.08
245.13
428.37
483.47
29-hydroxy-β-amyrin
in GuCYP72A154
x10 2
134.07
103.05
73.09
216.12
245.04
306.23
Compound (1) in
GgCYP72A154
x10 3
73.09
291.23
190.15
114.99
249.20
30-hydroxy-β-amyrin
in GgCYP72A154
x10 3
73.09
148.08
201.15
109.03
306.30
245.09
Compound (1) in
GpCYP72A154
x10 2
291.22
75.06
149.12
129.14
207.05
249.05
Counts vs. Mass-to-Charge (m/z)

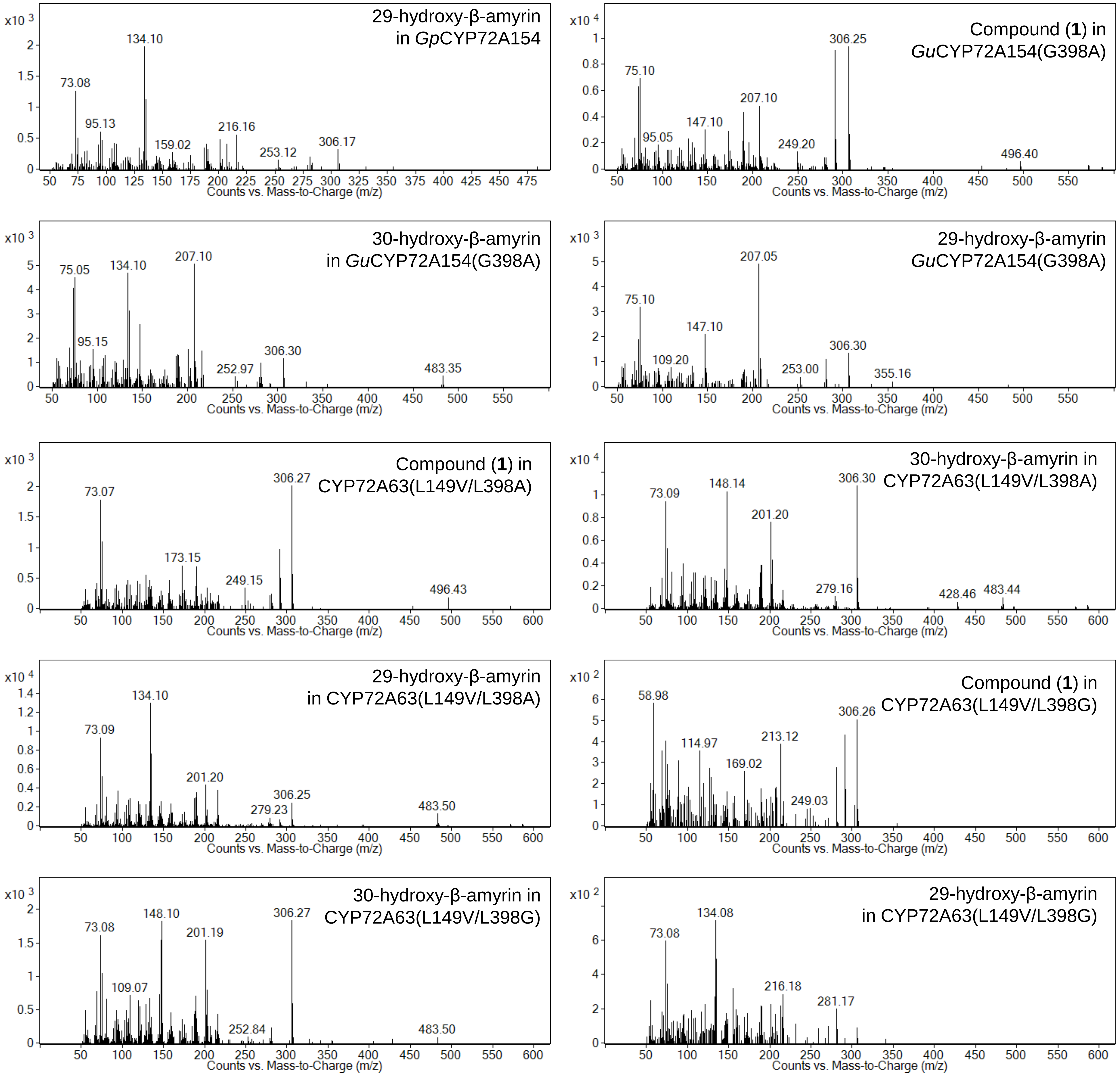
29-hydroxy-β-amyrin in GpCYP72A154
x10 3
134.10
73.08
95.13
159.02
216.16
253.12
306.17
Counts vs. Mass-to-Charge (m/z)
Compound (1) in GuCYP72A154(G398A)
x10 4
306.25
75.10
207.10
147.10
95.05
249.20
496.40
Counts vs. Mass-to-Charge (m/z)
30-hydroxy-β-amyrin in GuCYP72A154(G398A)
x10 3
75.05
134.10
207.10
95.15
306.30
252.97
483.35
Counts vs. Mass-to-Charge (m/z)
29-hydroxy-β-amyrin GuCYP72A154(G398A)
x10 3
207.05
75.10
147.10
109.20
306.30
253.00
355.16
Counts vs. Mass-to-Charge (m/z)
Compound (1) in CYP72A63(L149V/L398A)
x10 3
306.27
73.07
173.15
249.15
496.43
Counts vs. Mass-to-Charge (m/z)
30-hydroxy-β-amyrin in CYP72A63(L149V/L398A)
x10 4
73.09
148.14
306.30
201.20
279.16
428.46
483.44
Counts vs. Mass-to-Charge (m/z)
29-hydroxy-β-amyrin in CYP72A63(L149V/L398A)
x10 4
134.10
73.09
201.20
306.25
279.23
483.50
Counts vs. Mass-to-Charge (m/z)
Compound (1) in CYP72A63(L149V/L398G)
x10 2
58.98
306.26
114.97
213.12
169.02
249.03
Counts vs. Mass-to-Charge (m/z)
30-hydroxy-β-amyrin in CYP72A63(L149V/L398G)
x10 3
148.10
73.08
201.19
306.27
109.07
252.84
483.50
Counts vs. Mass-to-Charge (m/z)
29-hydroxy-β-amyrin in CYP72A63(L149V/L398G)
x10 2
134.08
73.08
216.18
281.17
Counts vs. Mass-to-Charge (m/z)

(F)

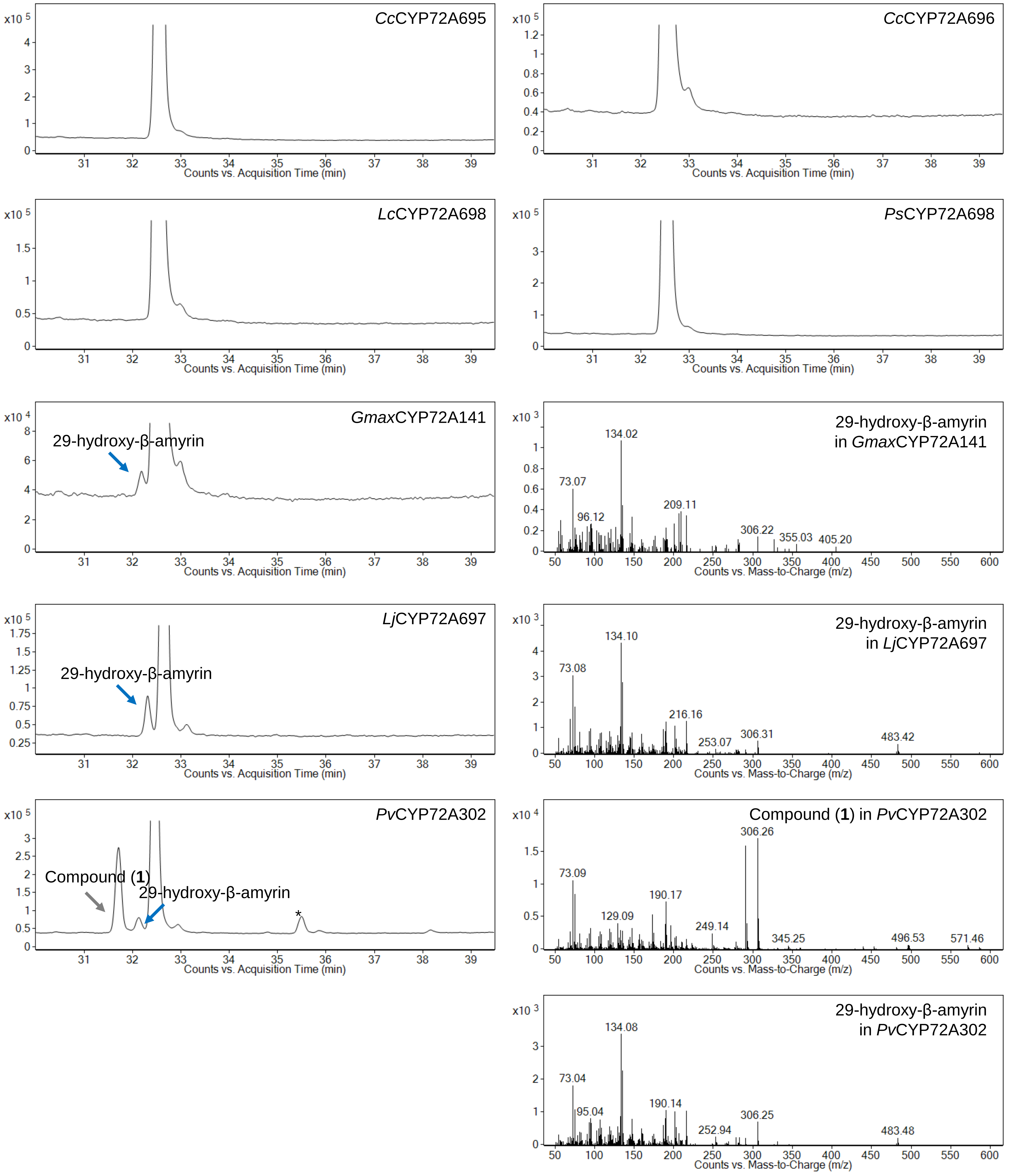

CcCYP72A695
CcCYP72A696
LcCYP72A698
PsCYP72A698
GmaxCYP72A141
29-hydroxy-β-amyrin
29-hydroxy-β-amyrin in GmaxCYP72A141
134.02
73.07
96.12
209.11
306.22
355.03
405.20
LjCYP72A697
29-hydroxy-β-amyrin
29-hydroxy-β-amyrin in LjCYP72A697
134.10
73.08
216.16
253.07
306.31
483.42
PvCYP72A302
Compound (1)
29-hydroxy-β-amyrin
*
Compound (1) in PvCYP72A302
306.26
73.09
190.17
129.09
249.14
345.25
496.53
571.46
29-hydroxy-β-amyrin in PvCYP72A302
134.08
73.04
95.04
190.14
306.25
252.94
483.48
Counts vs. Acquisition Time (min)
Counts vs. Mass-to-Charge (m/z)
x10 5
x10 4
x10 3

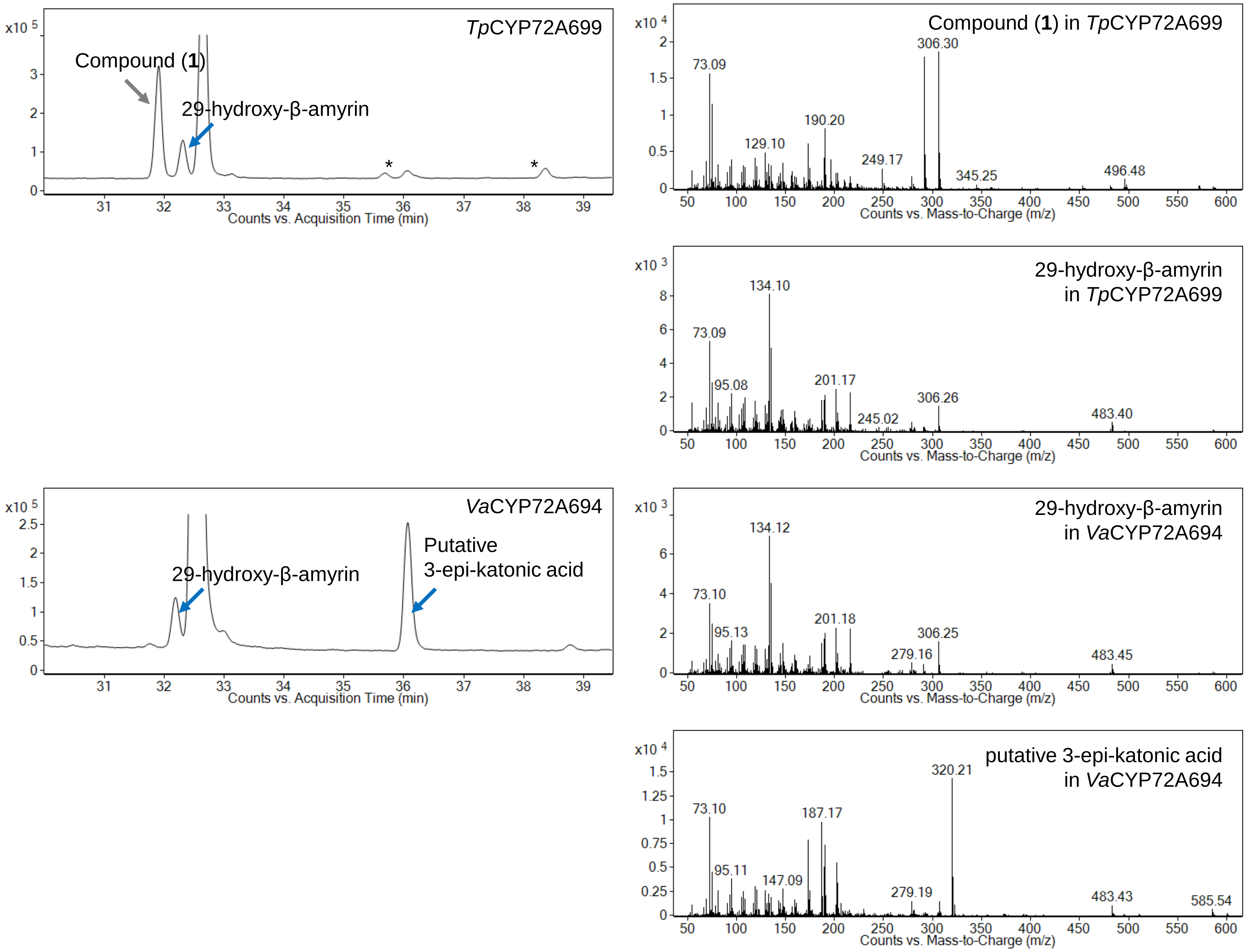

Supplementary Figure 1. Gas chromatogram and mass spectra. **(A)** Total ion current (TIC) chromatogram and mass spectra of compounds from Figure 2. **(B)** TIC chromatogram and mass spectra of CYP72A62 mutants. **(C)** Mass spectra of compounds from Figure 3C. **(D)** Mass spectra of compounds from Figure 4B. **(E)** Mass spectra of compounds from Figure 5. **(F)**. TIC Chromatogram and mass spectra of *in vivo* enzymatic assay from Figure 6.
