# Supplementary material for: Molecular Basis of C-30 Product Regioselectivity of Legume Oxidases Involved in High-Value Triterpenoid Biosynthesis
Source: Front Plant Sci. 2019 Nov 26;10:1520. doi: 10.3389/fpls.2019.01520 (PMC6901910; doi:10.3389/fpls.2019.01520)
Supplement: Supplementary file 1 [file DataSheet_1.zip › 11-01-2019_10.3389-fpls.2019.01520/Supplementary Figure S2.PDF]

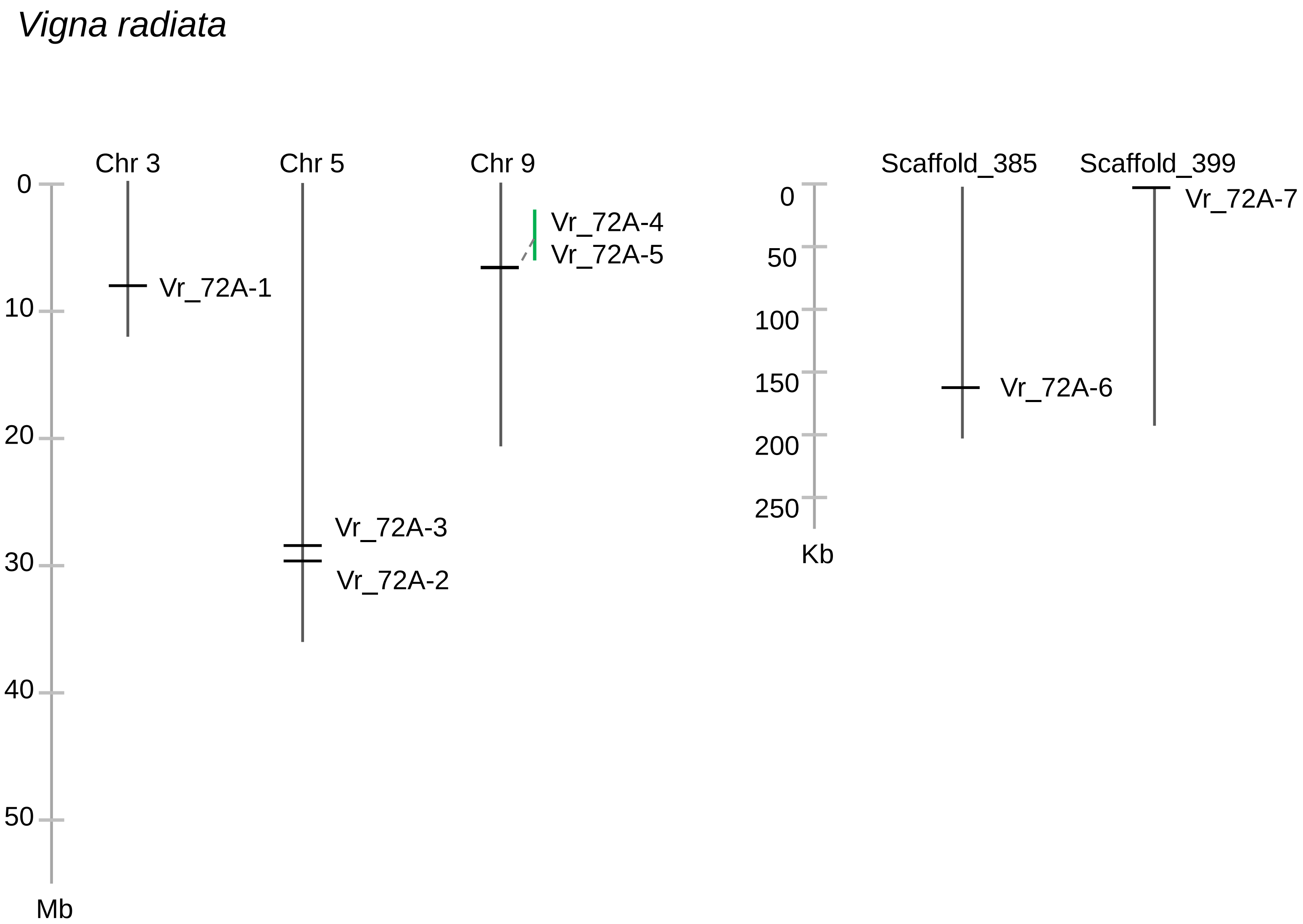
Vigna radiata
Chr 3
Chr 5
Chr 9
Scaffold_385
Scaffold_399
0
10
20
30
40
50
Mb
Vr_72A-1
Vr_72A-3
Vr_72A-2
Vr_72A-4
Vr_72A-5
0
50
100
150
200
250
Kb
Vr_72A-6
Vr_72A-7

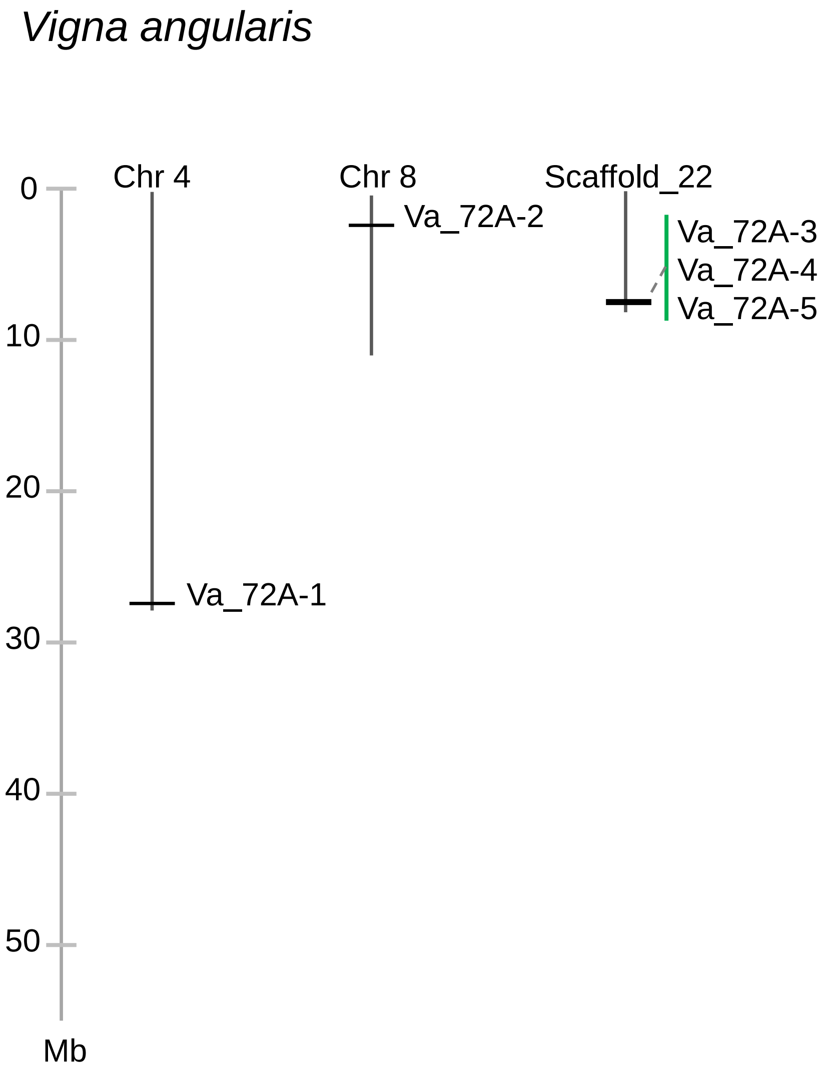
Vigna angularis
Chr 4
Chr 8
Scaffold_22
Va_72A-1
Va_72A-2
Va_72A-3
Va_72A-4
Va_72A-5
0
10
20
30
40
50
Mb

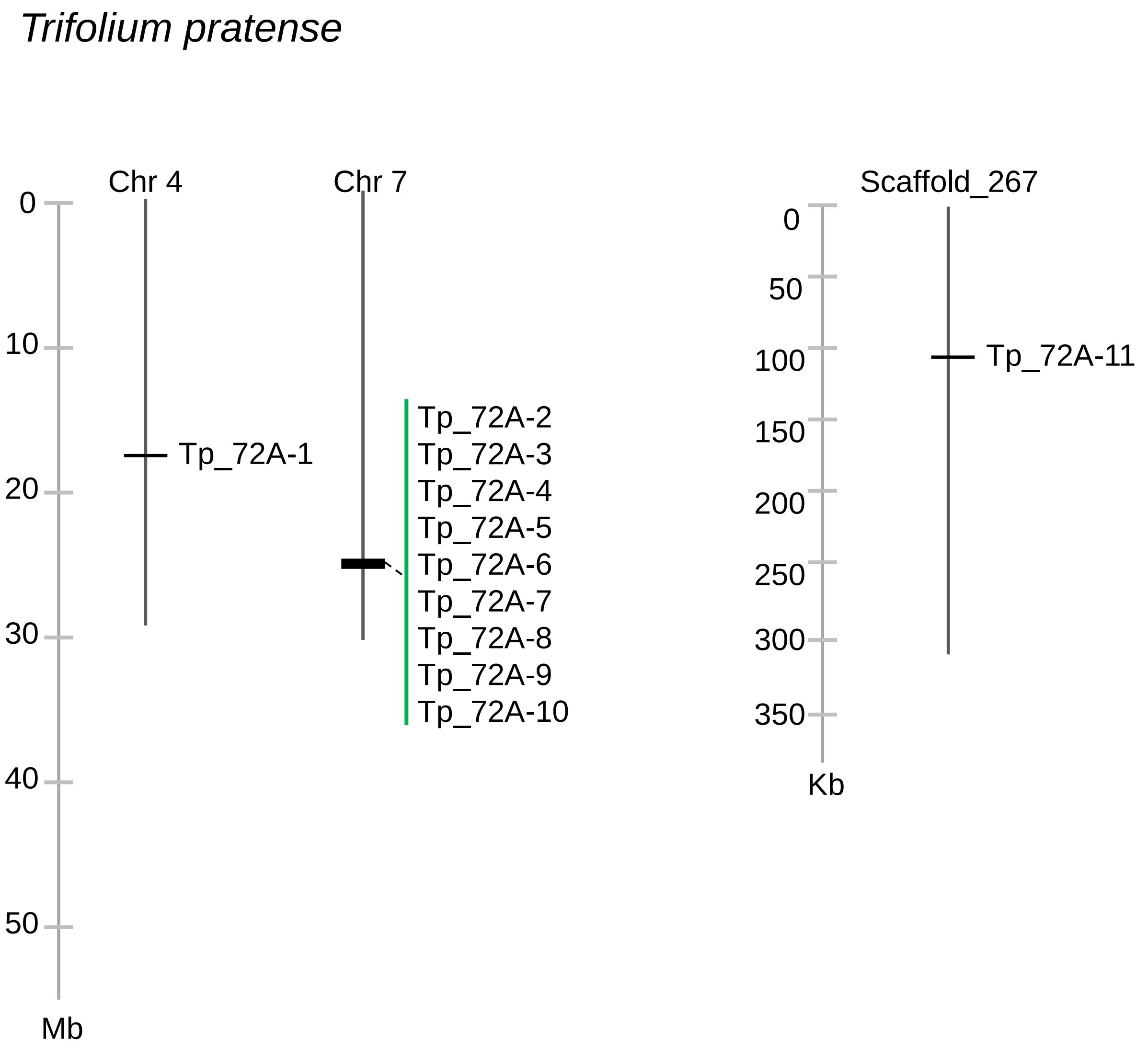
Trifolium pratense
Chr 4
Chr 7
Scaffold_267
0
10
20
30
40
50
Mb
Tp_72A-1
Tp_72A-2
Tp_72A-3
Tp_72A-4
Tp_72A-5
Tp_72A-6
Tp_72A-7
Tp_72A-8
Tp_72A-9
Tp_72A-10
0
50
100
150
200
250
300
350
Kb
Tp_72A-11

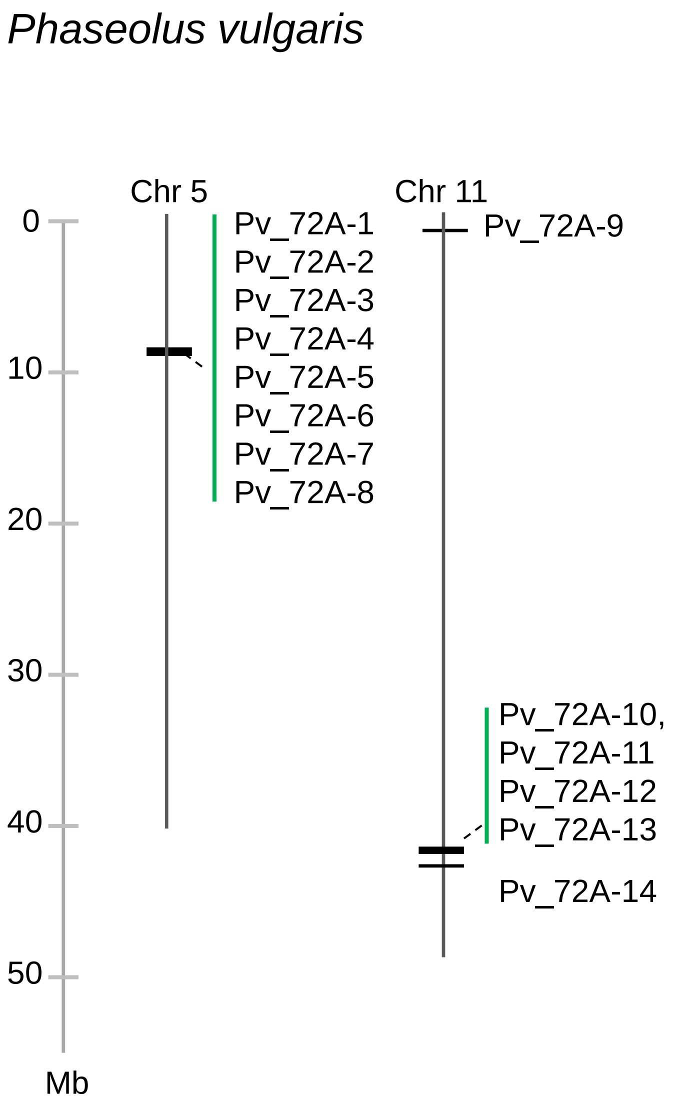
Phaseolus vulgaris
Chr 5
Chr 11
0
10
20
30
40
50
Mb
Pv_72A-1
Pv_72A-2
Pv_72A-3
Pv_72A-4
Pv_72A-5
Pv_72A-6
Pv_72A-7
Pv_72A-8
Pv_72A-9
Pv_72A-10,
Pv_72A-11
Pv_72A-12
Pv_72A-13
Pv_72A-14

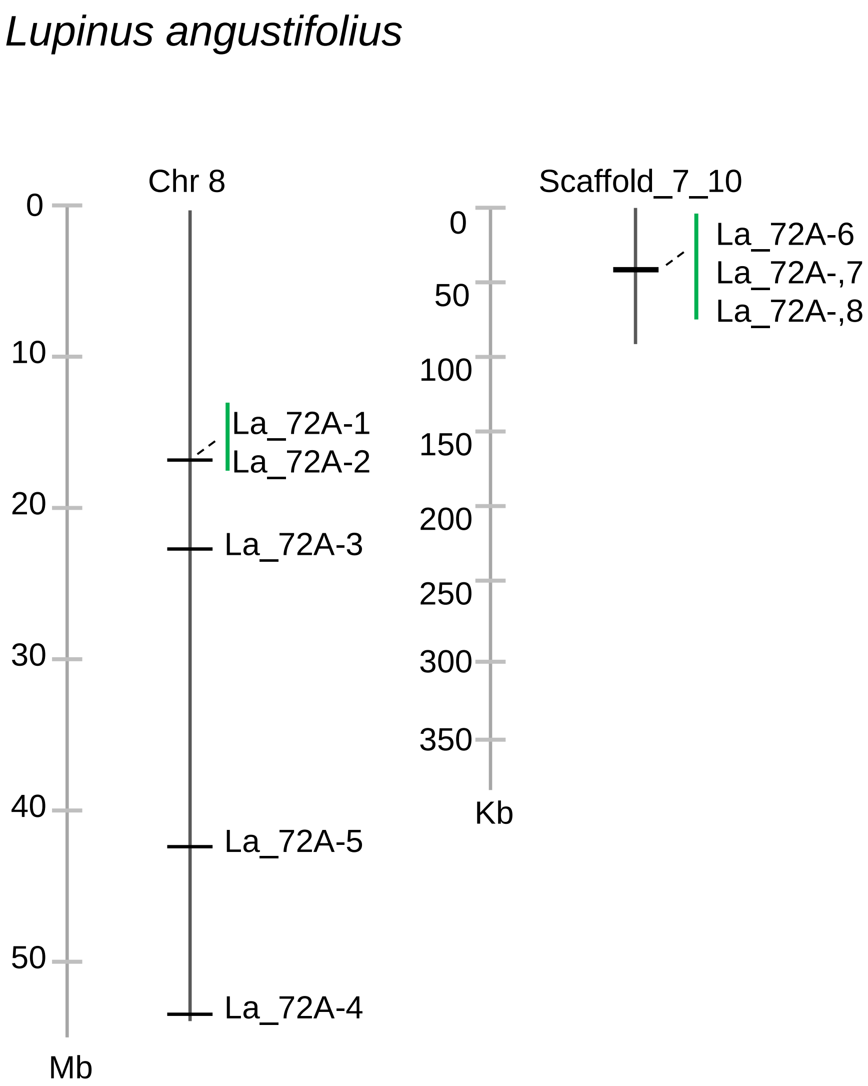

Lupinus angustifolius
Chr 8
0
10
20
30
40
50
Mb
La_72A-1
La_72A-2
La_72A-3
La_72A-5
La_72A-4
Scaffold_7_10
0
50
100
150
200
250
300
350
Kb
La_72A-6
La_72A-,7
La_72A-,8

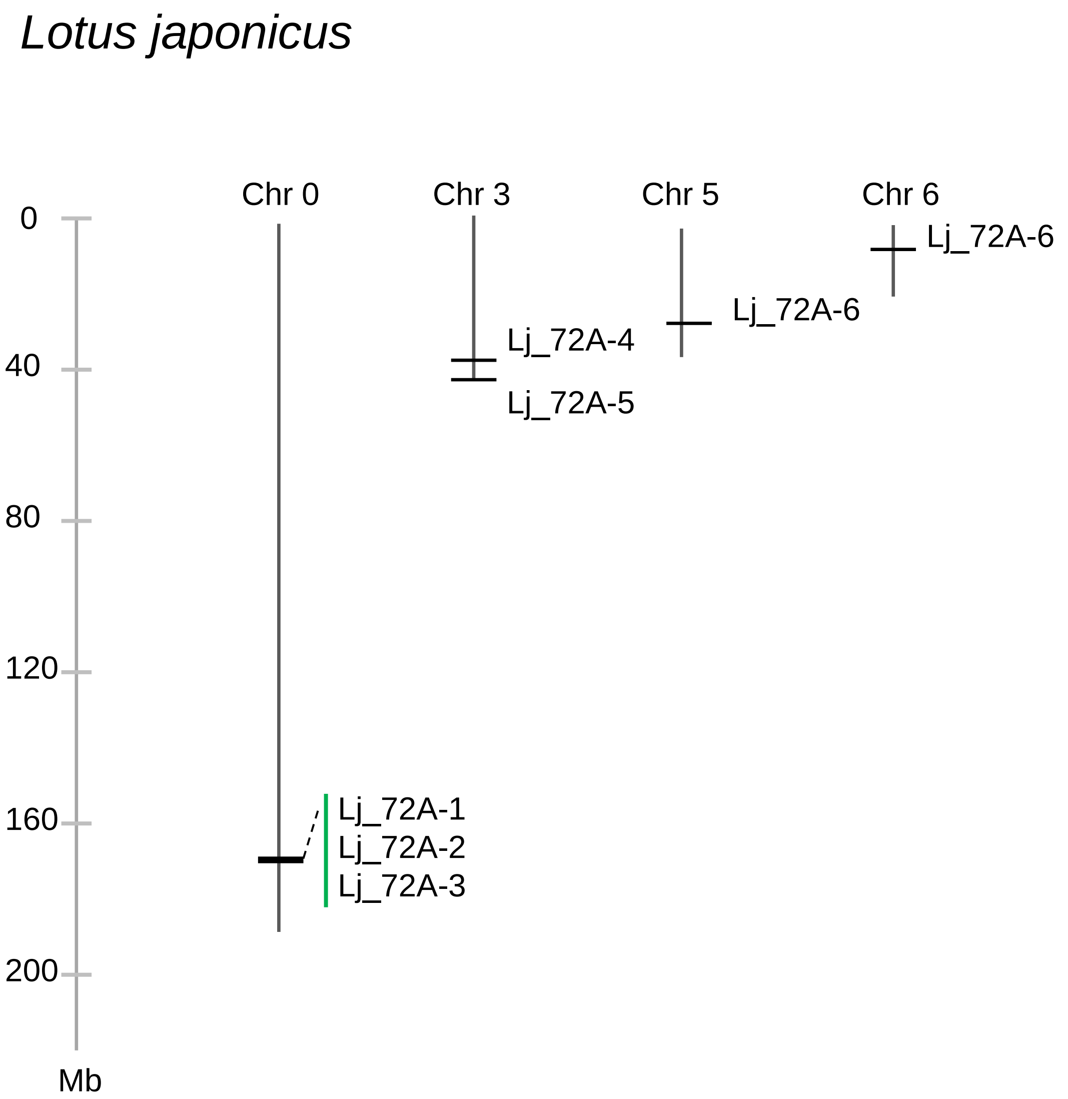
Lotus japonicus
Chr 0
Chr 3
Chr 5
Chr 6
0
40
80
120
160
200
Mb
Lj_72A-1
Lj_72A-2
Lj_72A-3
Lj_72A-4
Lj_72A-5
Lj_72A-6
Lj_72A-6

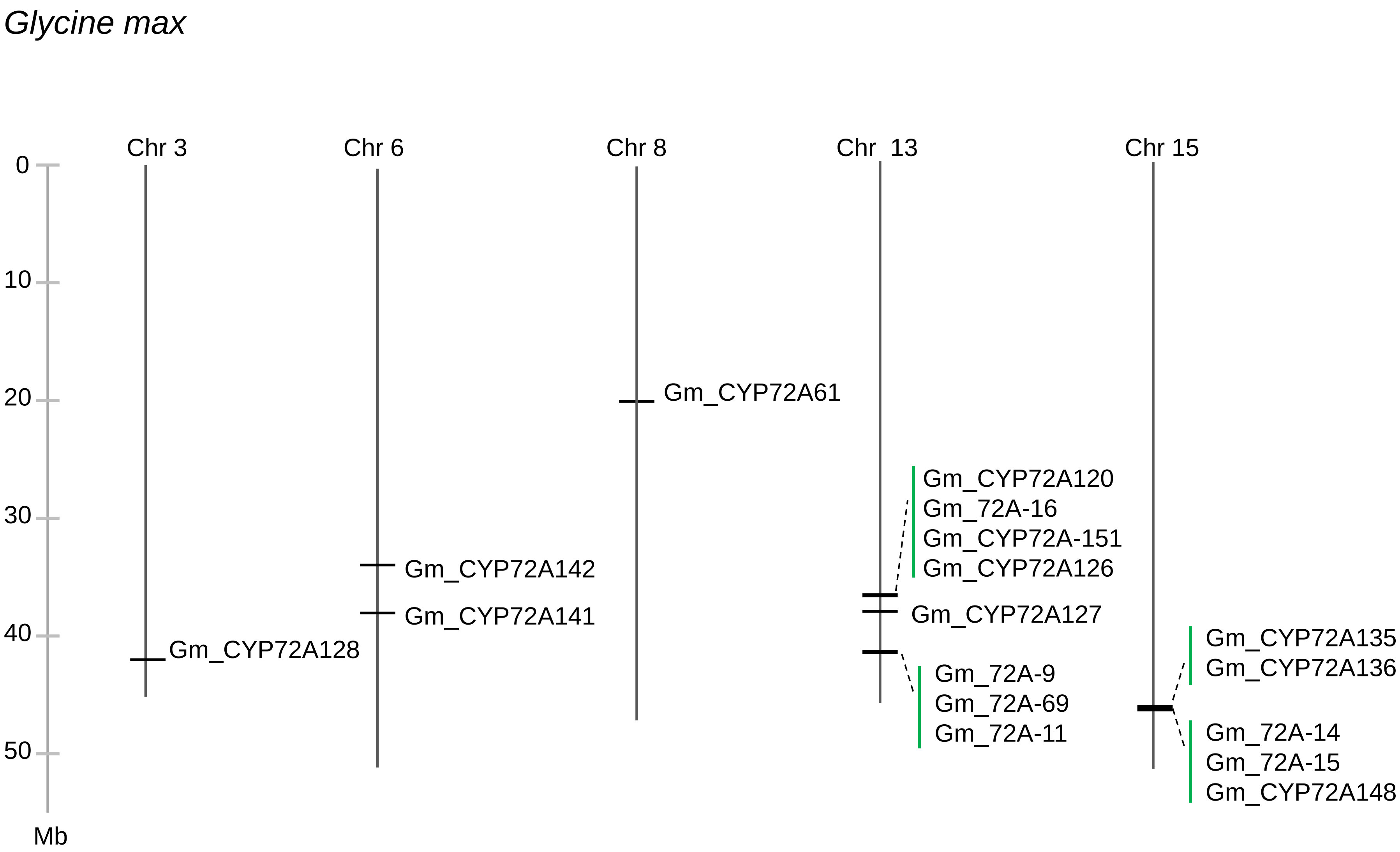

Glycine max
Chr 3
Chr 6
Chr 8
Chr 13
Chr 15
0
10
20
30
40
50
Mb
Gm_CYP72A128
Gm_CYP72A142
Gm_CYP72A141
Gm_CYP72A61
Gm_CYP72A120
Gm_72A-16
Gm_CYP72A-151
Gm_CYP72A126
Gm_CYP72A127
Gm_72A-9
Gm_72A-69
Gm_72A-11
Gm_CYP72A135
Gm_CYP72A136
Gm_72A-14
Gm_72A-15
Gm_CYP72A148

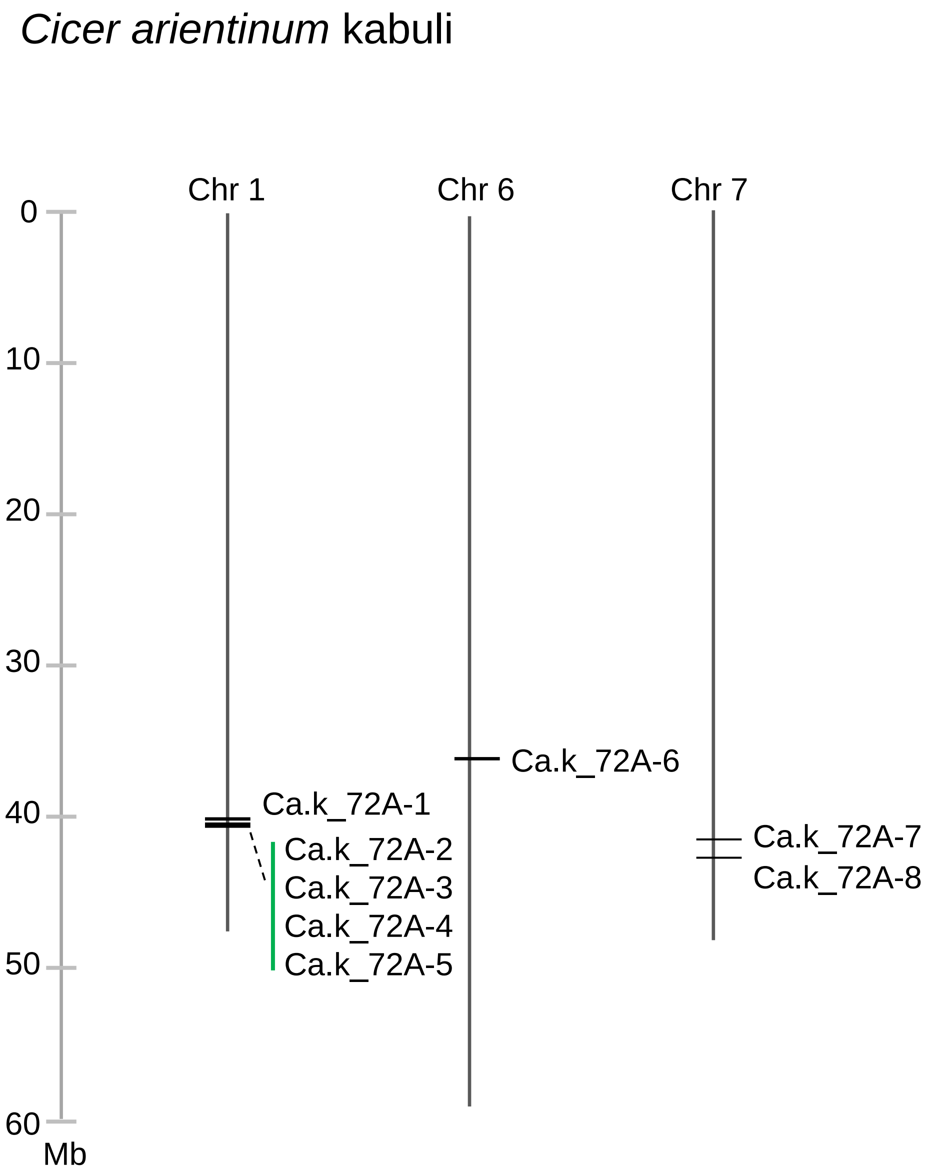

Cicer arientinum kabuli
Chr 1
Chr 6
Chr 7
0
10
20
30
40
50
60
Mb
Ca.k_72A-1
Ca.k_72A-2
Ca.k_72A-3
Ca.k_72A-4
Ca.k_72A-5
Ca.k_72A-6
Ca.k_72A-7
Ca.k_72A-8

Cicer arientinum desi

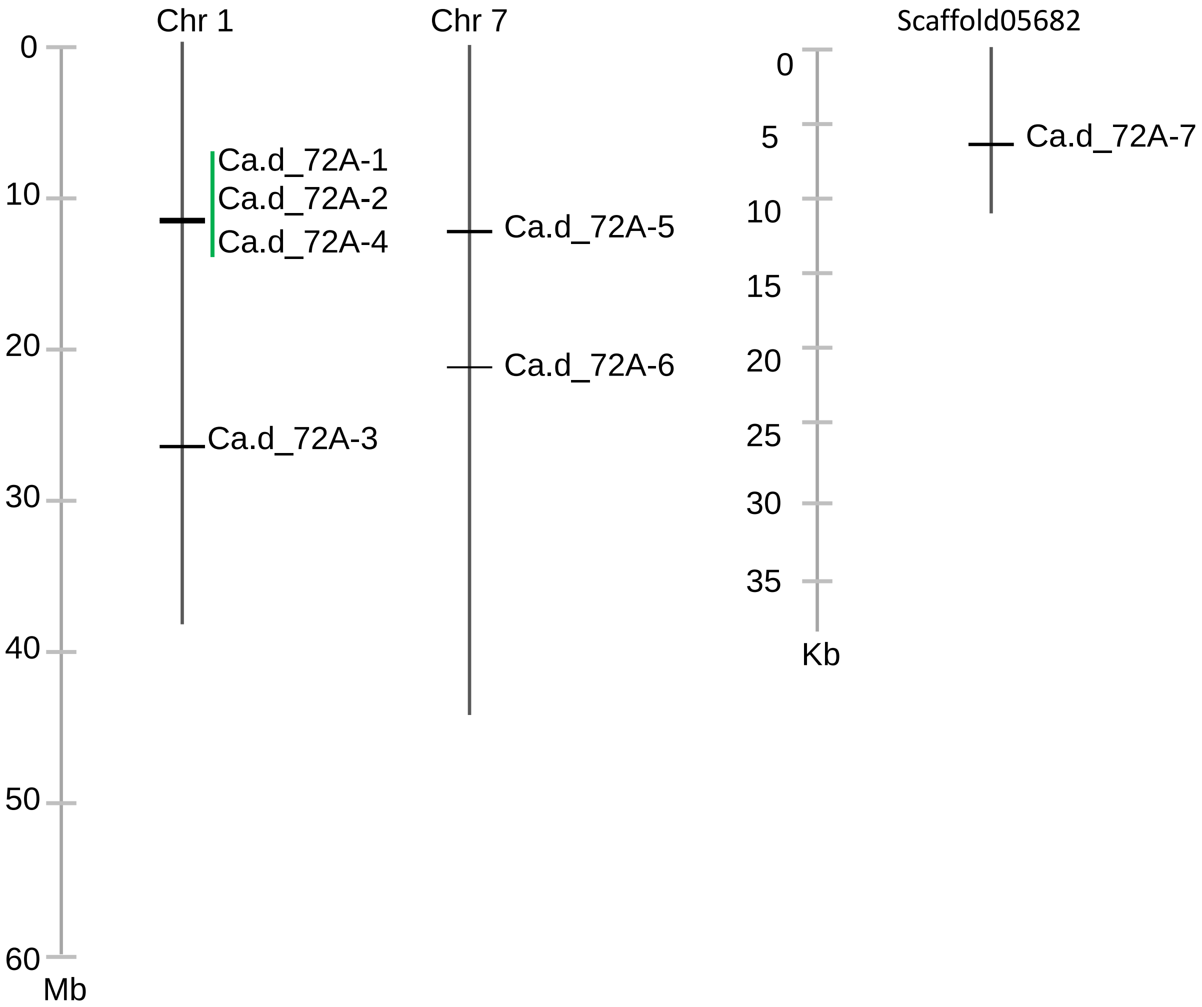

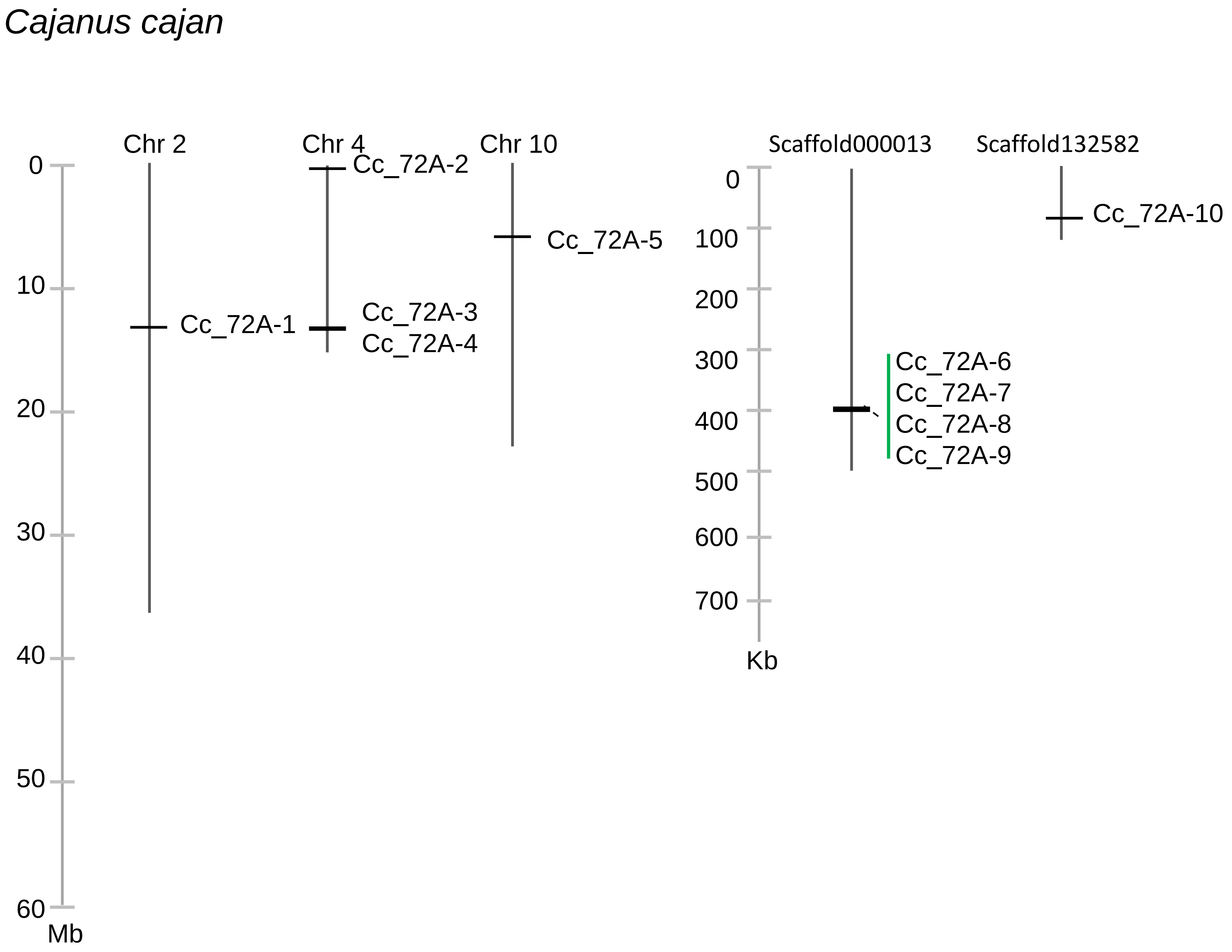
Cajanus cajan
Chr 2
Chr 4
Chr 10
Scaffold000013
Scaffold132582
0
10
20
30
40
50
60
Mb
Cc_72A-1
Cc_72A-2
Cc_72A-3
Cc_72A-4
Cc_72A-5
0
100
200
300
400
500
600
700
Kb
Cc_72A-6
Cc_72A-7
Cc_72A-8
Cc_72A-9
Cc_72A-10

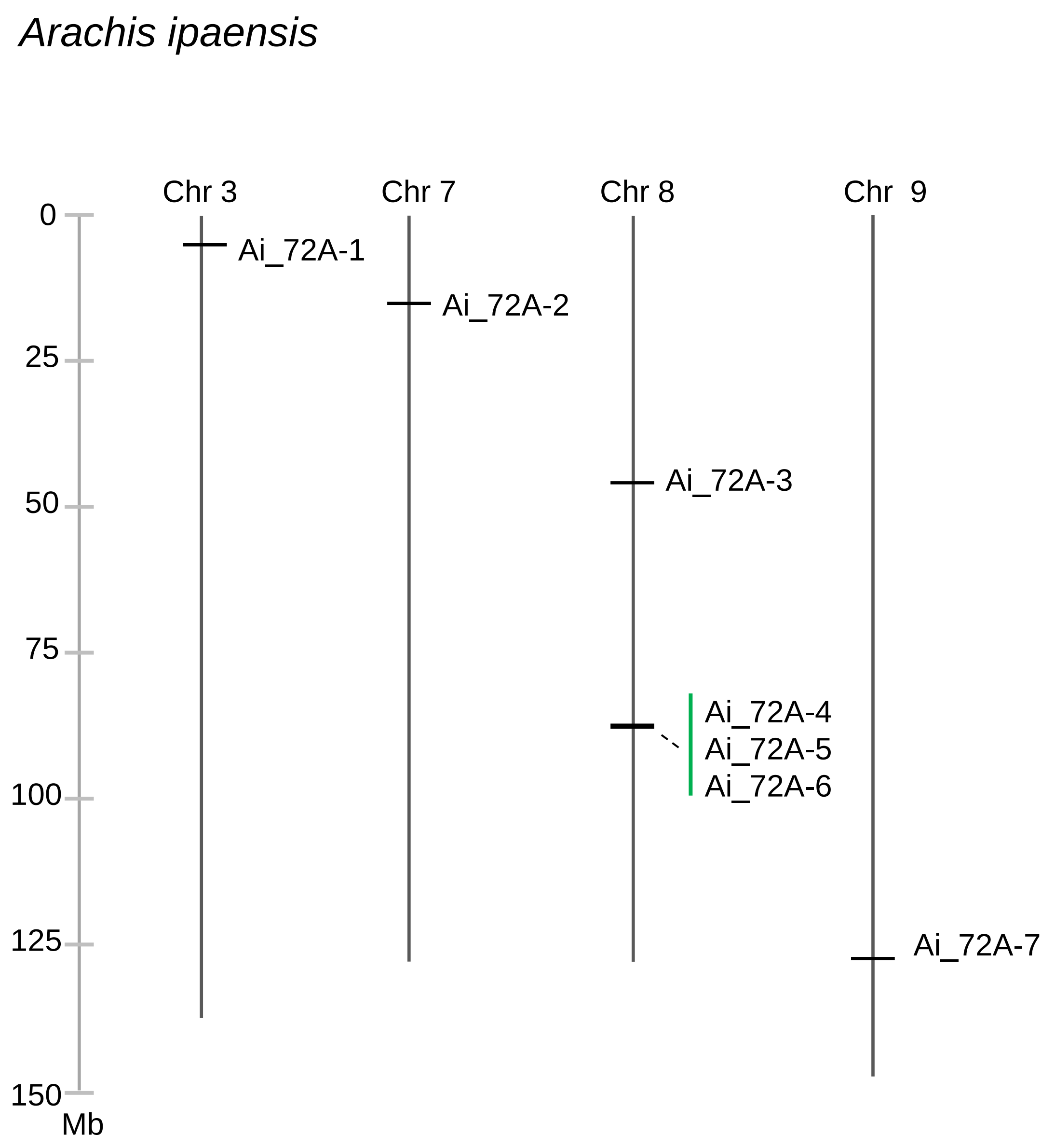
Arachis ipaensis
Chr 3
Chr 7
Chr 8
Chr 9
0
25
50
75
100
125
150
Mb
Ai_72A-1
Ai_72A-2
Ai_72A-3
Ai_72A-4
Ai_72A-5
Ai_72A-6
Ai_72A-7

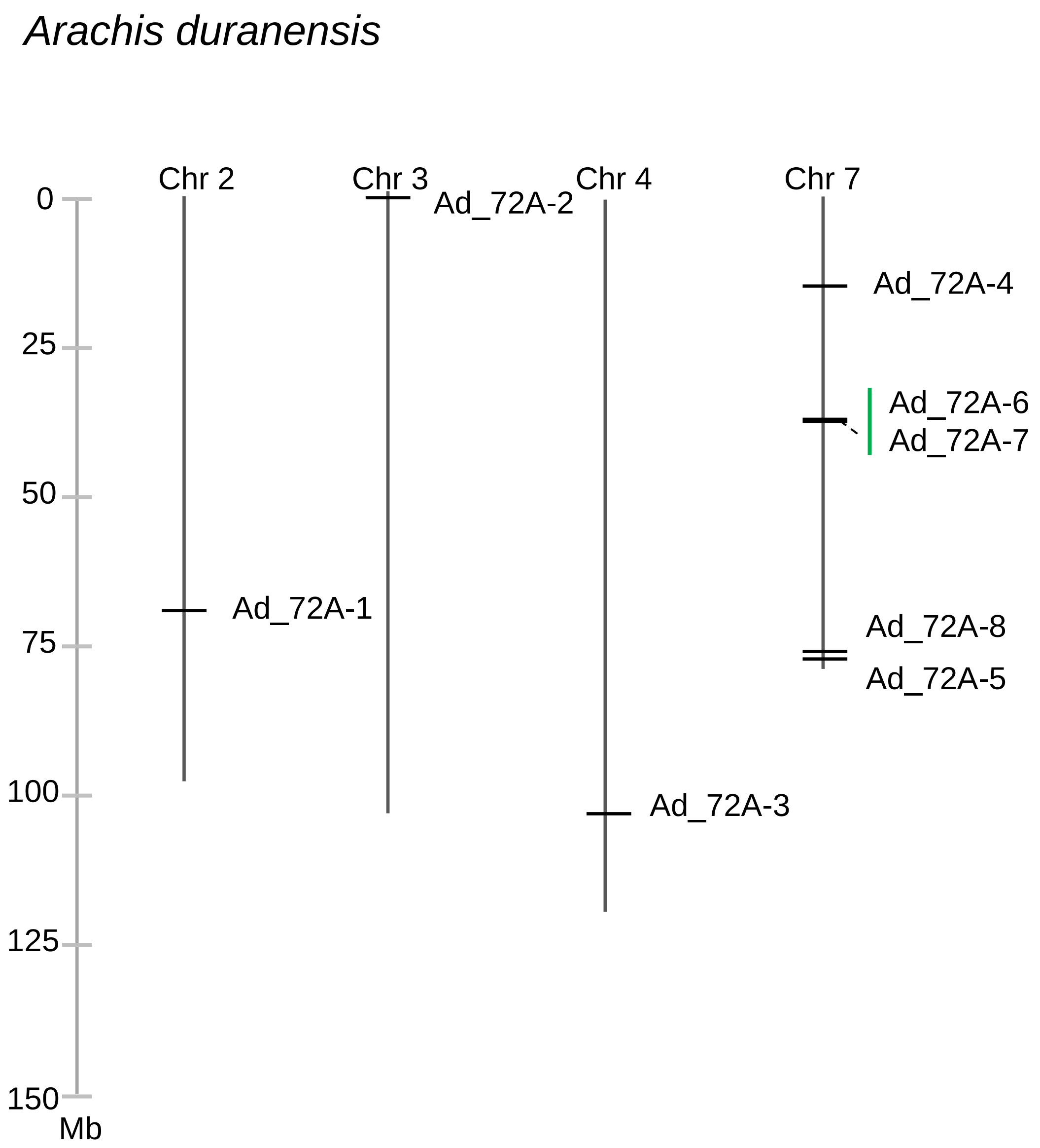

Supplementary Figure 2. Chromosomal localization of the CYP72A subfamily in legumes. Gene positions on the map were constructed based on the JBrowser feature in Legume Information System (LIS) (Dash et al., 2016), with the scale as indicated.
