# Supplementary material for: Molecular Basis of C-30 Product Regioselectivity of Legume Oxidases Involved in High-Value Triterpenoid Biosynthesis
Source: Front Plant Sci. 2019 Nov 26;10:1520. doi: 10.3389/fpls.2019.01520 (PMC6901910; doi:10.3389/fpls.2019.01520)
Supplement: Supplementary file 1 [file DataSheet_1.zip › 11-01-2019_10.3389-fpls.2019.01520/Supplementary Figure S3.PDF]

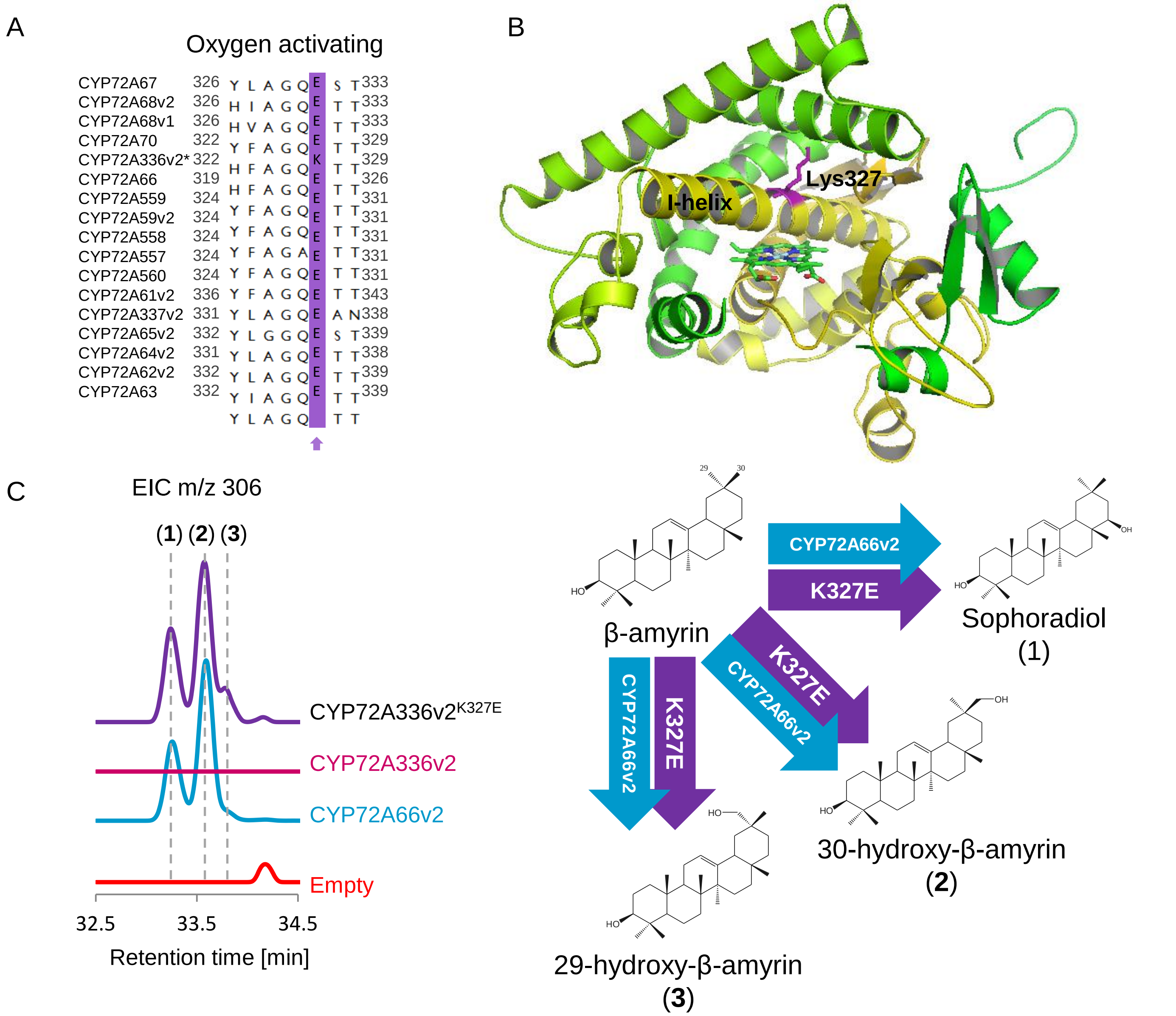

Supplementary Figure 3. Gain-of-function experiment in CYP72A336v2. (A) Sequence alignment of oxygen-activating region. Important amino acid residues are shown in purple. **(B)** Location of Lys327 in the CYP72A336v2 model. Residue Lys327 is shown in purple. **(C)** *In vivo* enzymatic assay of CYP72A336v2^{K327E}. CYP72A336v2^{K327E} was generated using a PrimeSTAR Mutagenesis Basal Kit (TaKaRa Bio, Kyoto, Japan). Enzymatic activity was tested by yeast *in vivo* enzyme assay and enzymatic product was analyzed by chromatography–mass spectrometry (GC-MS) analysis. β-Amyrin-oxidized products were detected in CYP72A336v2^{K327E} by yeast *in vivo* assay. These gain-of-function experimental results indicated that mutation in amino acid residue #327 abolished the enzymatic activity of CYP72A336v2.
