# Supplementary material for: Molecular Basis of C-30 Product Regioselectivity of Legume Oxidases Involved in High-Value Triterpenoid Biosynthesis
Source: Front Plant Sci. 2019 Nov 26;10:1520. doi: 10.3389/fpls.2019.01520 (PMC6901910; doi:10.3389/fpls.2019.01520)
Supplement: Supplementary file 1 [file DataSheet_1.zip › 11-01-2019_10.3389-fpls.2019.01520/Supplementary Figure S4.PDF]

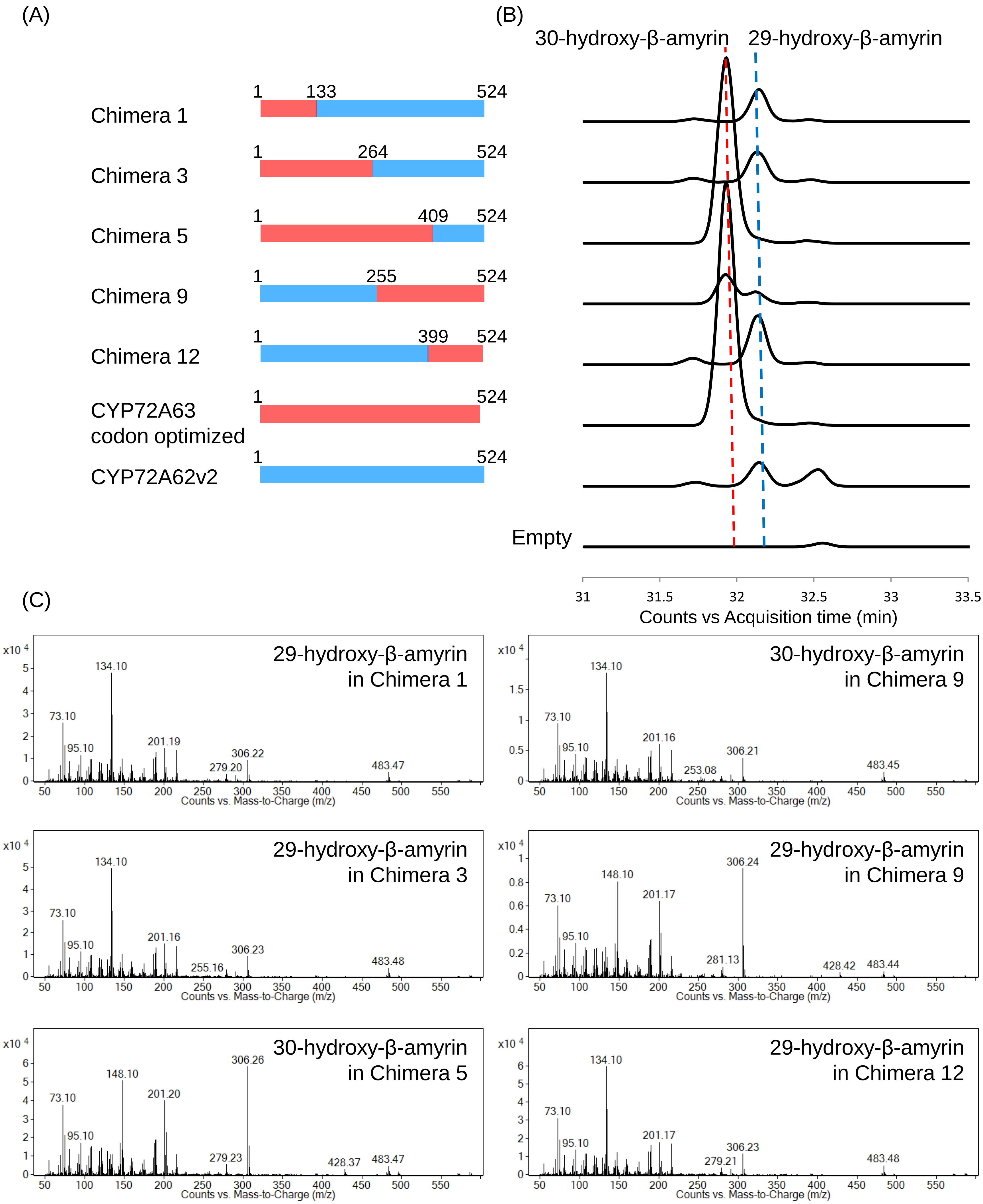

Supplementary Figure 4. Mapping of amino acid residues involved in C-30 product regioselectivity by segment swapping. **(A)** Segment map of chimeras. Chimeras were constructed by polymerase chain reaction (PCR), cloned into pELC-Leu2d, and confirmed by sequencing. pELC-CPR-GW, pYES2-DEST52, and pESC-HIS were generated by LR reaction. **(B)** *In vivo* enzymatic assay of chimeras. *In vivo* enzymatic assay was performed by co-expressing each chimeric enzyme with β-amyrin synthase and CPR in *S. cerevisiae* INVSc1. **(C)** Mass spectra of peaks detected on GC analysis. *In vivo* enzymatic assay of chimeras indicated that the amino acid residues important for C-30 product regioselectivity are located between positions #133 and #409.
