# Supplementary material for: Molecular Basis of C-30 Product Regioselectivity of Legume Oxidases Involved in High-Value Triterpenoid Biosynthesis
Source: Front Plant Sci. 2019 Nov 26;10:1520. doi: 10.3389/fpls.2019.01520 (PMC6901910; doi:10.3389/fpls.2019.01520)
Supplement: Supplementary file 1 [file DataSheet_1.zip › 11-01-2019_10.3389-fpls.2019.01520/Supplementary Methods.PDF]

Supplementary Methods

Total RNA of legumes, except for *G. pallidflora* and *G. glabra*, was isolated using PureLink® Plant RNA reagent (Thermo Fisher Scientific, USA) and treated with recombinant DNaseI (RNase-free) (Takara Bio, Japan). Then, sample was purified using RNeasy® Plant Mini Kit (Qiagen, Germany). cDNA was synthesized using PrimeScriptTM RT Master Mix kit (Takara Bio, Japan). Selected candidates were amplified with gene specific oligonucleotides (Supplementary Table 2) and ENTRy clone were generated by either pENTR™-D-TOPO® (Invitrogen, USA) or NEBuilder® HiFi DNA Assembly Cloning Kit (NEB, USA).

Glycyrrhiza pallidiflora was harvested in the medicinal plant garden of Chiba University (Chiba, Japan). The total RNA extraction from the under ground parts and the preparation of cDNA template were carried out as previously described (Seki et al., 2008). GpCYP72A154 cDNA fragment was PCR amplified with a primer set (primers 1 and 2) based on the GuCYP72A154 cDNA sequence. 5'- and 3'-RACE PCR were performed using primers 3 and 4. The full length coding sequence of GpCYP72A154 cDNA was PCR amplified with a couple of primers 5 and 6, and cloned into a pENTR/D-TOPO vector (Invitrogen).

Glycyrrhiza glabra underground part was obtained from the Health Sciences University of Hokkaido, Japan. The full length CDS of GgCYP72A154 was PCR amplified with primers 7 and 8 that were used to amplify the full length CDS of CYP72A154 (Seki et al., 2011), and cloned into a pENTR/D-TOPO vector (Invitrogen). The both end sequences of GgCYP72A154 cDNA were confirmed by RACE PCR experiments with primers 3 and 4.

Primer	Sequence (5' -> 3')
1	ATGGATGCATCTTCCAC
2	TTACAGTTTATGCAGAATG
3	TGGAGAGACCAATGGATTTTGATTGTGC
4	TTGGATGGGGTCCTAGAATATGTGTTGG
5	CACCATGGATGCATCTTCCACACCTG
6	TTACAGTTTATGCAGAATGATGGGTGCC
7 (Seki et al., 2011)	CACCATGGATGCATCTTCCACACCAG
8 (Seki et al., 2011)	TTACAGTTTATGCAGAATGATGGGTGCC
