# Supplementary material for: Molecular Basis of C-30 Product Regioselectivity of Legume Oxidases Involved in High-Value Triterpenoid Biosynthesis
Source: Front Plant Sci. 2019 Nov 26;10:1520. doi: 10.3389/fpls.2019.01520 (PMC6901910; doi:10.3389/fpls.2019.01520)
Supplement: Supplementary file 1 [file DataSheet_1.zip › 11-01-2019_10.3389-fpls.2019.01520/Supplementary Table S1.PDF]

Supplementary Table 1. Plant seeds used in the experiments

SName	Ecotype/Cultivar	Source	Detail
Medicago truncatula	R108	Laboratory collection	
Cajanus cajan	COL/INDONESIA/1991/JIRCAS/1	NARO, Japan	
Glycine max	SHIROSAYA	NARO, Japan	
Glycine soja	COL/GIFU/1983/OGURI	NARO, Japan	
Lotus japonicus	Gifu	Laboratory collection	
Lens culinaris subsp. culinaris	Araucana	USDA, USA	
Trifolium pratense	n.a	Takii Seed Co., Ltd, Japan	https://shop.takii.co.jp/shop/
Pisum sativum	Midoriusui	Takayama Seed Co., Ltd, Japan	http://www.takayama-seed.co.jp/index.html
Phaseolus vulgaris	YUKIWARIMAME	NARO, Japan	
Vigna angularis	cv. Erimoshouzu	NARO, Japan	
