# Supplementary material for: Molecular Basis of C-30 Product Regioselectivity of Legume Oxidases Involved in High-Value Triterpenoid Biosynthesis
Source: Front Plant Sci. 2019 Nov 26;10:1520. doi: 10.3389/fpls.2019.01520 (PMC6901910; doi:10.3389/fpls.2019.01520)
Supplement: Supplementary file 1 [file DataSheet_1.zip › 11-01-2019_10.3389-fpls.2019.01520/Supplementary Table S2.PDF]

Supplementary Table 2. List primer used in the experiments

Target	Forward	Reverse	Detail
CYP72A64	CACCATGGAGGTGTTTGTGTTTCCCACAAC	TTACAGTTTATGCAAAATGATGCTTGCCCCA	Cloning
CYP72A336	CACCATGGAATGGGTTTCTGGG	TTATGTTTCCAGTTTGCGTATAATAATATGAGCG	Cloning
CYP72A66	CACCATGGAATGGGTTTCAGG	TTATGTTTCCACTTTGCGTATAATAATATGAGCAC	Cloning
CYP72A70	CACCATGGAATGGTATTCAAGCATAATTCC	TTATGTTTCCAGTTTACGAAGAATAATATGAGCAC	Cloning
CYP72A337	CACCATGATGATGGGAGTATCTTCAACAAC	CTATAATTTGTGGAAAATGATATGAGCCCCATGTT	Cloning
CYP72A557/560	CACCATGGCTACAATTACATCC	TTACAATTCCACTTTACGTAGAATTATATGAG	Cloning
CYP72A558	CACCATGGCTACAACTACAGCA	TTACAATTCCACTTTACGTAGAATTATATGAG	Cloning
CYP72A559	CACCATGGCTACAACTACAGCAATAGTTC	TTATGTTGCCACTTTACGTAGAATAAGATGAA	Cloning
VaCYP72A694	GCCGCCCCCTTCACCATGATTACTCTGTACC	GGCGCGCCCACCCTTTCATAGGTTTTGCAAAAT	Cloning
PvCYP72A302	GCCGCCCCCTTCACCATGGAGCTATCGTTATCT	GGCGCGCCCACCCTTTCATAGCATTTGCAAAATAAGA	Cloning
GmaxCYP72A141	GCCGCCCCCTTCACCATGGAGCCATTATTTTC	GGCGCGCCCACCCTTTCATAGTTTATGCAAAACGA	Cloning
GsCYP72A141	GCCGCCCCCTTCACCATGGAGCCATTATTTTC	GGCGCGCCCACCCTTTCATAGTTTATGCAAAACGA	Cloning
CcCYP72A695	GCCGCCCCCTTCACCATGGAGGTAGT	GGCGCGCCCACCCTTTTATAGTTGATG	Cloning
CcCYP72A696	GCCGCCCCCTTCACCATGGAGGTACT	GGCGCGCCCACCCTTTTATAGTTGATG	Cloning
LcCYP72A698	GCCGCCCCCTTCACCATGGAGGTGTT	GGCGCGCCCACCCTTTTACAATTTATG	Cloning
PsCYP72A698	GCCGCCCCCTTCACCATGGAGGTGTTTATTTCC	GGCGCGCCCACCCTTTTACAATTTATGCAAAATGATGGTT	Cloning
TpCYP72A699	GCCGCCCCCTTCACCATGGAGGAGTTTGTGTTTC	GGCGCGCCCACCCTTTTACAATTTATGCAAAATGA	Cloning
LjCYP72A697	GCCGCCCCCTTCACCATGGAGCTGCTTTCAATAATC	GGCGCGCCCACCCTTCTACACTTTATGCAAAATGAG	Cloning
CYP72A62v2^{V149L}	GGTATATTACATTATGAGGGTAAGAAA	ATAATGTAATATACCAACGCTCAAATA	Mutagenesis
CYP72A62v2 ^{H150D}	ATAGTAGATTATGAGGGTAAGAAATGG	CTCATAATCTACTATACCAACGCTCAA	Mutagenesis
CYP72A62v2 ^{I244T}	TTGACGACAGGGCGACAAACGAACAAT	TCGCCCTGTCGTCAAAAGAAATCCCTG	Mutagenesis
CYP72A62v2^{Q246H}	GGGCGACATACGAACAATACATTATGG	GTTCGTATGTCGCCCTATCGTCAAAAG	Mutagenesis
CYP72A62v2 ^{E269D}	GAAATTGATAAAGAAATTCATGATTCA	TTCTTTATCAATTTCTTTCATCTTCGT	Mutagenesis
CYP72A62v2^{V398L}	CCACCTCTAATTTACTTCAACCGAGCT	GTAAATTAGAGGTGGGAATAACCTTAG	Mutagenesis
CYP72A336 ^{K327E}	GGTCAAGAGACTACTTCAGTTTTGCTT	AGTAGTCTCTTGACCTGCAAAGTGGAA	Mutagenesis

CYP72A63^{L149V}	GGTATAGTAGATCATGAGGGTAAGAAA	ATGATCTACTATACCAACGTTCAAATA	Mutagenesis
CYP72A63^{L398V}	CCACCTGTAATTTACTTCAACCGAGCT	GTAAATTACAGGTGGGAATAACCTTAG	Mutagenesis
CYP72A63^{L398I}	CCACCTATAATTTACTTCAACCGAGCT	GTAAATTATAGGTGGGAATAACCTTAG	Mutagenesis
CYP72A63^{L149I}	GGTATAATAGATCATGAGGGTAAGAAA	ATGATCTATTATACCAACGTTCAAATA	Mutagenesis
CYP72A63^{L398A}	CCACCTGCAATTTACTTCAACCGAGCT	GTAAATTGCAGGTGGGAATAACCTTAG	Mutagenesis
CYP72A63^{L398G}	CCACCTGGAATTTACTTCAACCGAGCT	GTAAATTCCAGGTGGGAATAACCTTAG	Mutagenesis
GuCYP72A154^{G398A}	CCACCTGCGATTTACCTCACCCGAGCT	GTAAATCGCAGGTGGGTACAGCCTGAG	Mutagenesis
