# Supplementary material for: Molecular Basis of C-30 Product Regioselectivity of Legume Oxidases Involved in High-Value Triterpenoid Biosynthesis
Source: Front Plant Sci. 2019 Nov 26;10:1520. doi: 10.3389/fpls.2019.01520 (PMC6901910; doi:10.3389/fpls.2019.01520)
Supplement: Supplementary file 1 [file DataSheet_1.zip › 11-01-2019_10.3389-fpls.2019.01520/Supplementary Table S3.PDF]

Supplementary Table 3. Gene names and origins

Gene	Plant source	Source
CYP72A59v2	*Medicago truncatula*	Seki et al., 2011
CYP72A62v2	*Medicago truncatula*	Seki et al., 2011
CYP72A63	*Medicago truncatula*	Seki et al., 2011
CYP72A64v2	*Medicago truncatula*	this study
CYP72A65v2	*Medicago truncatula*	Seki et al., 2011
CYP72A66v2	*Medicago truncatula*	this study
CYP72A70	*Medicago truncatula*	this study
CYP72A336v2	*Medicago truncatula*	this study
CYP72A337v2	*Medicago truncatula*	this study
CYP72A557	*Medicago truncatula*	this study
CYP72A558	*Medicago truncatula*	this study
CYP72A559	*Medicago truncatula*	this study
CYP72A560	*Medicago truncatula*	this study
GuCYP72A154	*Glycyrrhiza uralensis*	Seki et al., 2011
GgCYP72A154	*Glycyrrhiza glabra*	this study
GpCYP72A154	*Glycyrrhiza pallidiflora*	this study
GmCYP72A154	*Glycyrrhiza macedonica*	this study
VaCYP72A694	*Vigna angularis*	this study
PvCYP72A302	*Phaseolus vulgaris*	this study
GmaxCYP72A141	*Glycin max*	this study
GsCYP72A141	*Glycin soja*	this study
CcCYP72A695	*Cajanus cajan*	this study
CcCYP72A696	*Cajanus cajan*	this study
LcCYP72A698	*Lens culinaris*	this study
PsCYP72A698	*Pisum sativum*	this study
TpCYP72A699	*Trifolium pratense*	this study
LjCYP72A697	*Lotus japonicus*	this study
