# Supplementary material for: Molecular Basis of C-30 Product Regioselectivity of Legume Oxidases Involved in High-Value Triterpenoid Biosynthesis
Source: Front Plant Sci. 2019 Nov 26;10:1520. doi: 10.3389/fpls.2019.01520 (PMC6901910; doi:10.3389/fpls.2019.01520)
Supplement: Supplementary file 1 [file DataSheet_1.zip › 11-01-2019_10.3389-fpls.2019.01520/Supplementary Table S4.PDF]

Supplementary Table 4. Gene expression analysis of CYP72A subfamily in *M. truncatula*

Name	ID	Tissue (Transcripts per Million)					
		Blade (SRX099059)	Bud (SRX099060)	Flower (SRX099061)	Nodule (SRX099057)	Root (SRX099062)	SeedPod (SRX099058)
CYP72A67	Medtr2g023680	13.3272	56.1615	24.22145	8.33659	13.8269	44.311
CYP72A68-430	Medtr2g055430	0.0863011	0.0739667	0.560863	18.4619	4.83615	0.436871
CYP72A68-470	Medtr2g055470	22.5932	52.9318	44.7721	16.9556	12.1063	69.6618
CYP72A68-like	Medtr2g055530	0	0	0	0.33144	0.200479	0
CYP72A336	Medtr2g072250	32.8718	15.6271	5.94593	0.0516989	1.87628	10.0846
CYP72A66	Medtr2g072260	6.86623	2.73596	11.9665	0.19059	0.80698	1.06656
CYP72A337	Medtr2g072270	2.16148	4.18013	5.09402	0.876886	2.17466	0.560818
CYP72A560	Medtr2g072310	2.55803	0.182701	1.88012	0.0843179	1.02003	94.5054
CYP72A557	Medtr2g072330	1.67248	0.8182423	0.466129	0.476641	1.51563	0.126992
CYP72A558	Medtr2g072340	0.370922	1.80148	11.6511	1.51607	2.54402	1.01653
CYP72A59	Medtr2g072380	2.70014	5.58682	30.1328	3.01599	2.30216	3.94516
CYP72A559	Medtr2g072400	0.356686	0.509513	1.87652	9.54678	12.0612	0.0751934
CYP72A59-like6	Medtr2g072410	0	0	0	0	0	0
CYP72A59-like7	Medtr2g072420	0	0	0.407075	0	0	1.66381
CYP72A61	Medtr4g031820	43.189	56.7668	54.3658	48.7244	50.0614	12.3564
CYP72A70	Medtr5g095230	0	0	0	1.20125	34.8222	0
CYP72A65	Medtr8g042000	11.5698	23.2564	37.5122	5.92834	9.80497	19.4235
CYP72A64	Medtr8g042020	0.345601	1.718	0.192515	0.656163	0.132299	0.524567
CYP72A63	Medtr8g042040	0.0648047	0.333256	2.10578	0.358864	0.124039	0.327877
CYP72A62	Medtr8g042060	1.67165	0.9193	30.101	7.50689	8.13342	3.39344

Data was retrieved from medicmine.jcvi.org/
