# Supplementary material for: Molecular Basis of C-30 Product Regioselectivity of Legume Oxidases Involved in High-Value Triterpenoid Biosynthesis
Source: Front Plant Sci. 2019 Nov 26;10:1520. doi: 10.3389/fpls.2019.01520 (PMC6901910; doi:10.3389/fpls.2019.01520)
Supplement: Supplementary file 1 [file DataSheet_1.zip › 11-01-2019_10.3389-fpls.2019.01520/Supplementary Table S5.PDF]

Supplementary Table 5. BlastP results from gene mining in Mtr4.0

Query	Subject ID	Align Lenght	% Identity	Query Range	Subject Range	E-value	Score
CYP72A67	Medtr2g023680.1	520	100	1...520	1...520	0	1070
	Medtr2g055470.1	520	68.27	1...520	1...520	0	766
	Medtr2g055430.1	520	68.46	1...520	1...520	0	764
	Medtr2g072250.1	511	68.88	10...520	6...516	0	753
	Medtr5g095230.1	513	66.08	8...520	4...516	0	724
	Medtr2g072400.1	519	63.58	1...518	1...516	0	713
	Medtr2g072340.1	517	64.02	5...519	1...517	0	711
	Medtr2g072260.1	499	68.74	22...520	15...513	0	693
	Medtr2g072380.1	505	62.18	16...519	13...492	0	641
	Medtr2g072270.1	512	57.23	13...518	14...523	0	610
	Medtr8g042000.1	513	56.53	11...518	13...524	0	588
	Medtr8g042020.1	513	54.78	11...518	13...523	0	583
	Medtr8g042060.1	513	54.39	11...518	13...524	0	578
	Medtr8g042040.1	513	53.61	11...518	13...507	0	557
	Medtr4g031820.1	512	51.76	10...518	19...528	0	545
	Medtr2g072310.1	352	69.6	168...519	1...352	0	540
	Medtr2g072330.1	519	45.86	5...519	1...415	9.00E-146	429
	Medtr2g055530.1	220	53.64	41...260	6...187	6.00E-72	231
	Medtr2g072420.1	140	67.86	380...519	3...142	1.00E-67	218
CYP72A63	Medtr8g042040.1	524	94.66	1...524	1...507	0	1015
	Medtr8g042060.1	524	93.32	1...524	1...524	0	1007
	Medtr8g042020.1	524	83.97	1...524	1...523	0	900
	Medtr8g042000.1	524	83.4	1...524	1...524	0	896
	Medtr2g072270.1	522	59	8...524	7...523	0	645
	Medtr2g072340.1	520	57.88	8...524	3...516	0	624
	Medtr2g072400.1	520	57.5	8...524	3...516	0	619
	Medtr2g072250.1	520	57.12	8...524	5...514	0	615
	Medtr5g095230.1	516	56.2	13...524	7...514	0	601
	Medtr2g023680.1	513	56.34	13...524	11...518	0	595
	Medtr2g072260.1	482	58.09	44...524	35...511	0	582
	Medtr2g055430.1	521	53.55	5...524	3...518	0	578
	Medtr2g055470.1	515	53.2	11...524	9...518	0	578
	Medtr2g072380.1	502	57.17	26...524	21...491	0	559
	Medtr4g031820.1	523	51.82	7...524	15...528	0	528
	Medtr2g072310.1	351	63.25	175...524	1...351	3.00E-164	474
	Medtr2g072330.1	321	45.79	8...325	3...313	3.00E-88	281
	Medtr2g072420.1	141	64.54	384...524	1...141	5.00E-64	209
	Medtr2g072330.1	170	52.94	355...524	276...414	4.00E-49	177
CYP72A61	Medtr4g031820.1	528	100	1...528	1...528	0	1086
	Medtr2g072400.1	518	58.3	13...528	1...516	0	654
	Medtr2g072340.1	515	58.06	16...528	5...516	0	649
	Medtr2g072250.1	512	55.08	19...528	6...514	0	610
	Medtr5g095230.1	512	52.34	19...528	6...514	0	587
	Medtr2g072380.1	500	56.6	31...528	19...491	0	578
	Medtr2g072270.1	524	50.57	11...528	3...523	0	570
	Medtr2g055470.1	515	51.26	16...528	7...518	0	566
	Medtr2g023680.1	512	51.76	19...528	10...518	0	563
	Medtr2g072260.1	480	55	51...528	35...511	0	561

	Medtr2g055430.1	515	50.87	16...528	7...518	0	558
	Medtr8g042060.1	522	51.92	15...528	7...524	0	549
	Medtr8g042020.1	522	50.38	15...528	7...523	0	527
	Medtr8g042000.1	524	50.76	15...528	7...524	0.00E+00	524
	Medtr8g042040.1	522	50.19	15...528	7...507	7.00E-177	513
	Medtr2g072310.1	352	58.52	179...528	1...351	8.00E-155	451
	Medtr2g072420.1	141	59.57	388...528	1...141	4.00E-59	196
	Medtr4g031820.1	528	100	1...528	1...528	0	1086
