# Supplementary material for: Molecular Basis of C-30 Product Regioselectivity of Legume Oxidases Involved in High-Value Triterpenoid Biosynthesis
Source: Front Plant Sci. 2019 Nov 26;10:1520. doi: 10.3389/fpls.2019.01520 (PMC6901910; doi:10.3389/fpls.2019.01520)
Supplement: Supplementary file 1 [file DataSheet_1.zip › 11-01-2019_10.3389-fpls.2019.01520/Supplementary Table S6.PDF]

Supplementary Table 6. Amino acid sequence identity of CYP72A subfamily in *M. truncatula*

	72A59v2	72A337 v2	72A557	72A558	72A559	72A560	72A61 v2	72A62 v2	72A63	72A64 v2	72A65 v2	72A66v2	72A336 v2	72A67	72A68v1	72A68v2	72A70
72A59v2																	
72A337v2	59																
72A557	88	58															
72A558	90	58	88														
72A559	84	60	83	85													
72A560	89	59	92	86	83												
72A61v2	56	49	55	54	56	54											
72A62v2	59	57	58	57	57	57	50										
72A63	58	58	58	56	57	58	50	93									
72A64v2	58	56	57	56	57	56	48	81	83								
72A65v2	58	58	59	56	57	58	49	83	83	82							
72A66v2	72	58	71	69	71	71	53	56	56	54	55						
72A336v2	72	59	70	70	73	71	53	55	55	53	55	87					
72A67	63	55	64	62	63	65	50	54	55	52	55	67	67				
72A68v1	63	52	63	63	64	64	50	53	52	53	54	69	70	68			
72A68v2	62	52	62	61	63	63	49	53	53	53	54	69	69	68	94		
72A70	70	57	68	68	70	69	50	55	55	53	55	76	79	65	68	68	
